# Supplementary figures and images for: The molecular mechanism and evolutionary divergence of caspase 3/7-regulated gasdermin E activation (part 1 of 2)
Source: eLife. 2024 Mar 15;12:RP89974. doi: 10.7554/eLife.89974 (PMC10942788; doi:10.7554/eLife.89974)

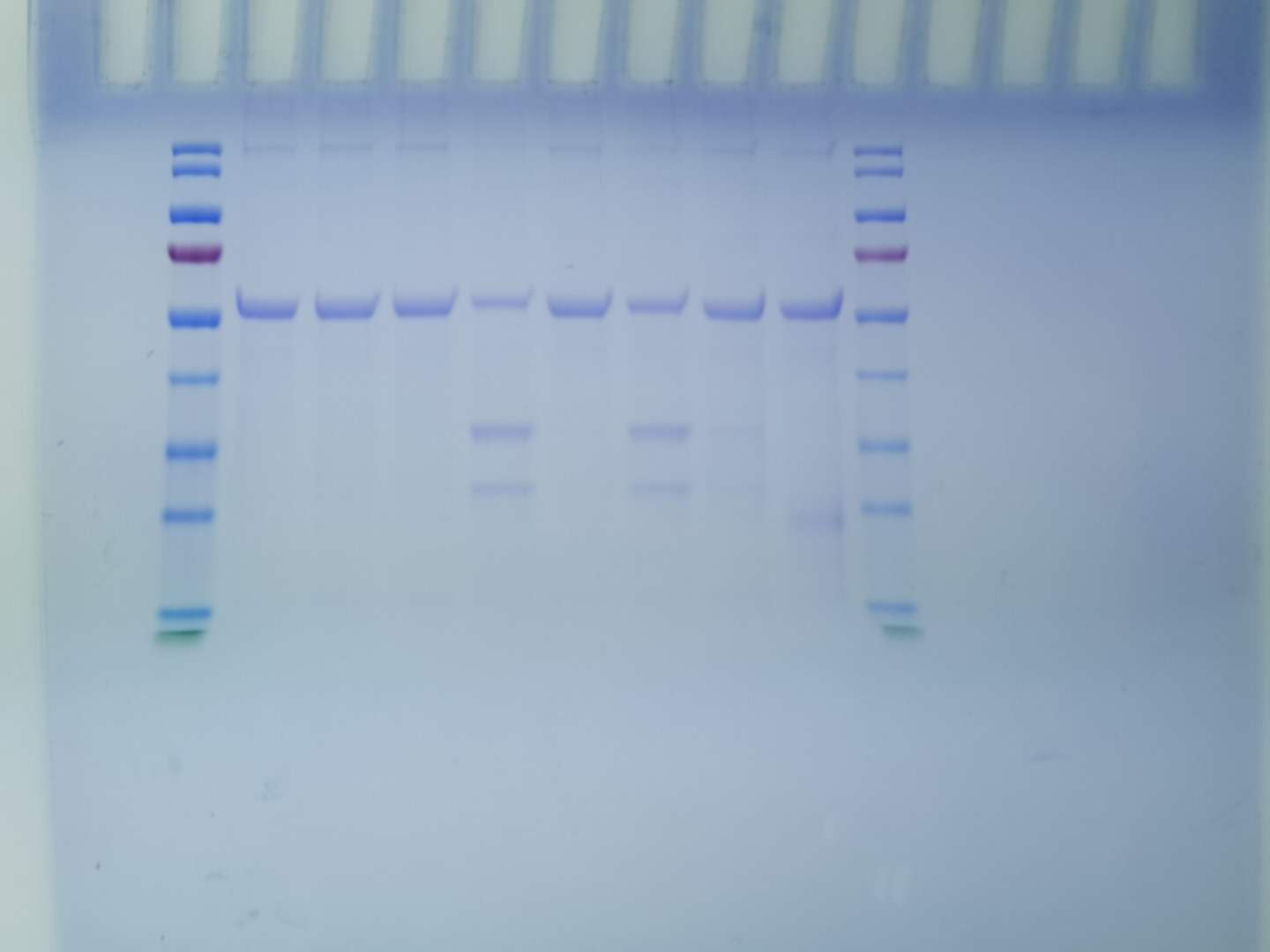

Supplement: Figure 1—source data 1. [file elife-89974-fig1-data1.zip › Figure 1-source data 1/Figure 1A-source data.tif]

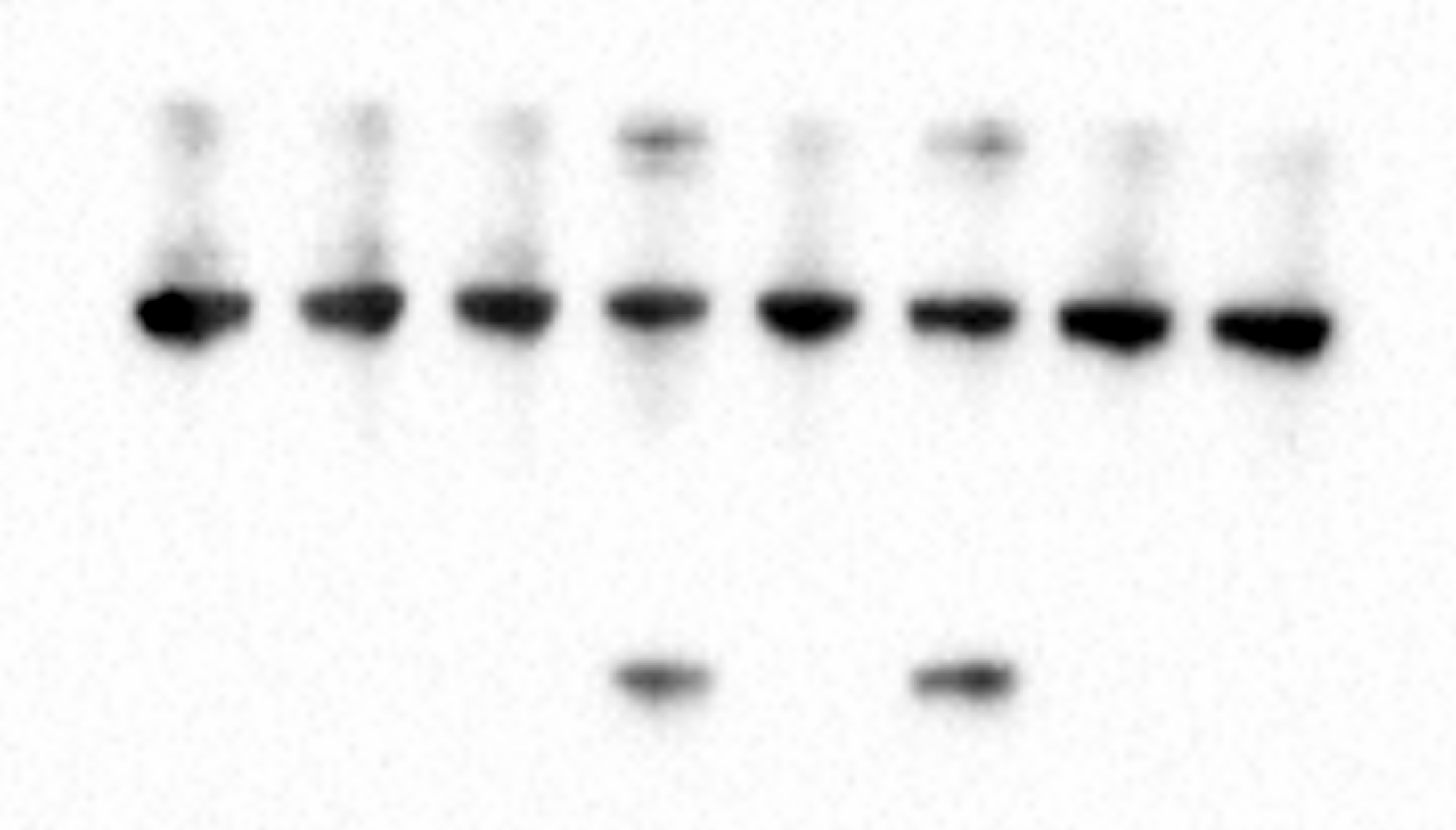

Supplement: Figure 1—source data 1. [file elife-89974-fig1-data1.zip › Figure 1-source data 1/Figure 1B-source data.tif]

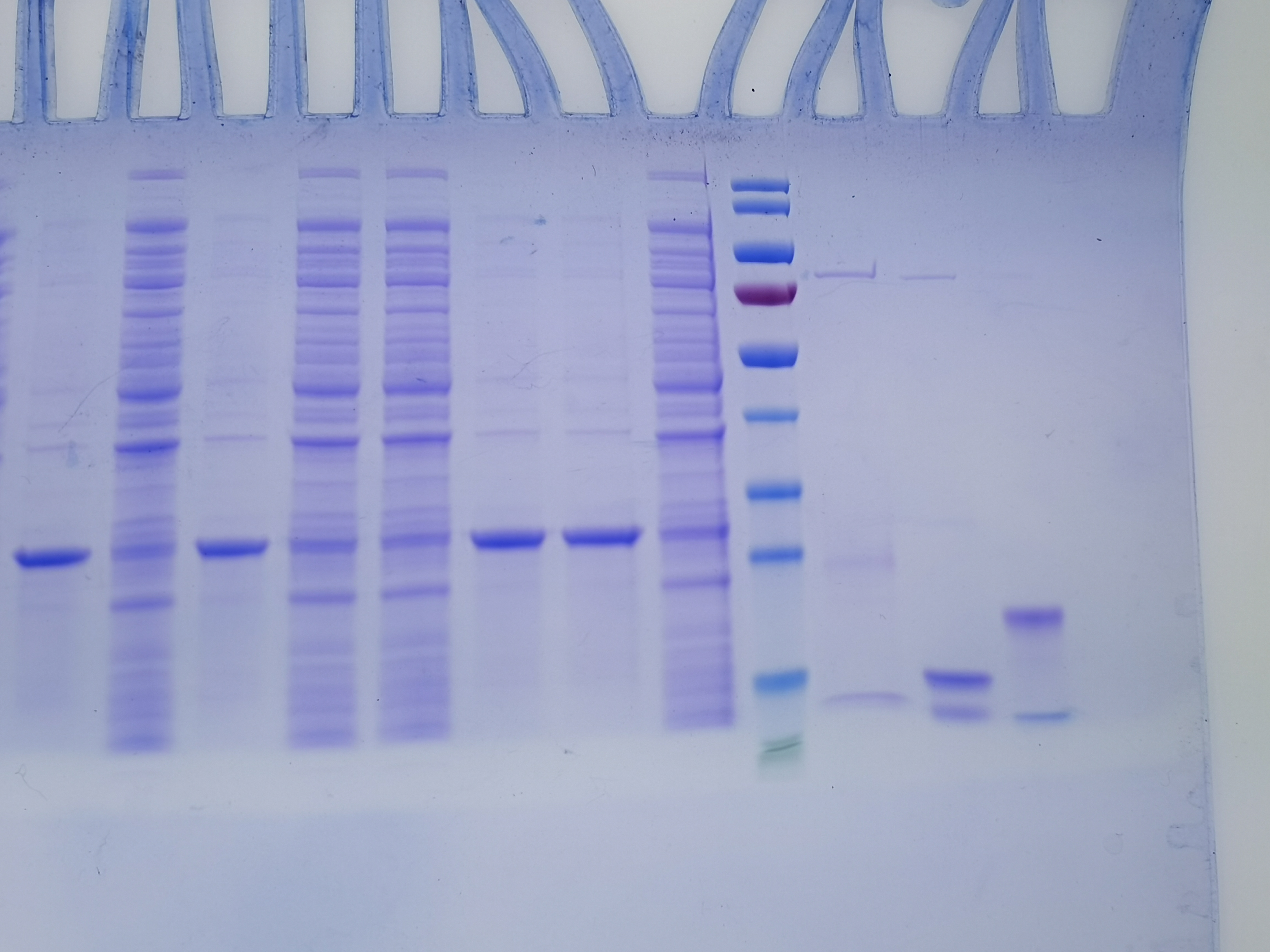

Supplement: Figure 1—source data 1. [file elife-89974-fig1-data1.zip › Figure 1-source data 1/Figure 1C-source data.tif]

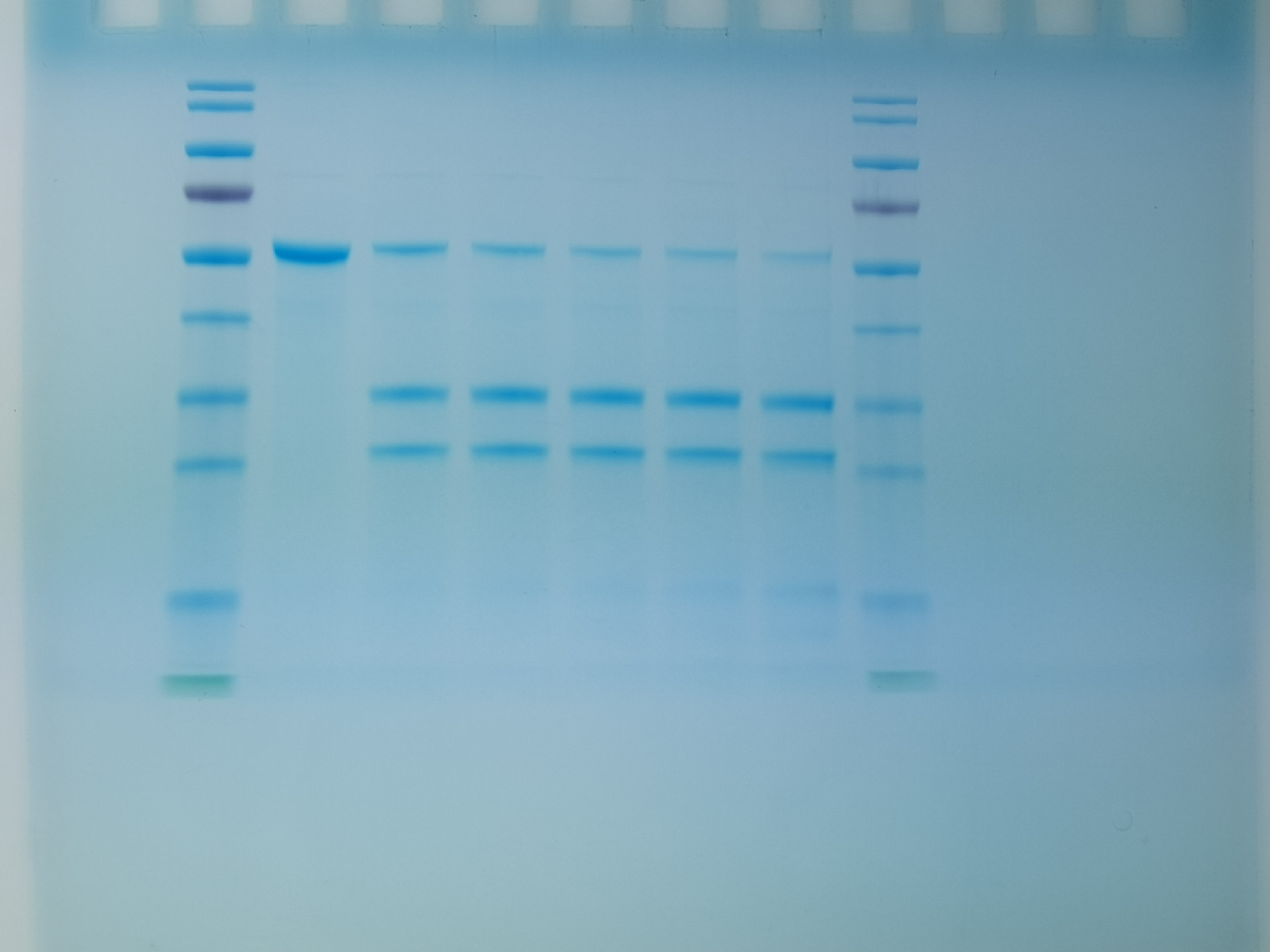

Supplement: Figure 1—source data 1. [file elife-89974-fig1-data1.zip › Figure 1-source data 1/Figure 1F-left-source data.tif]

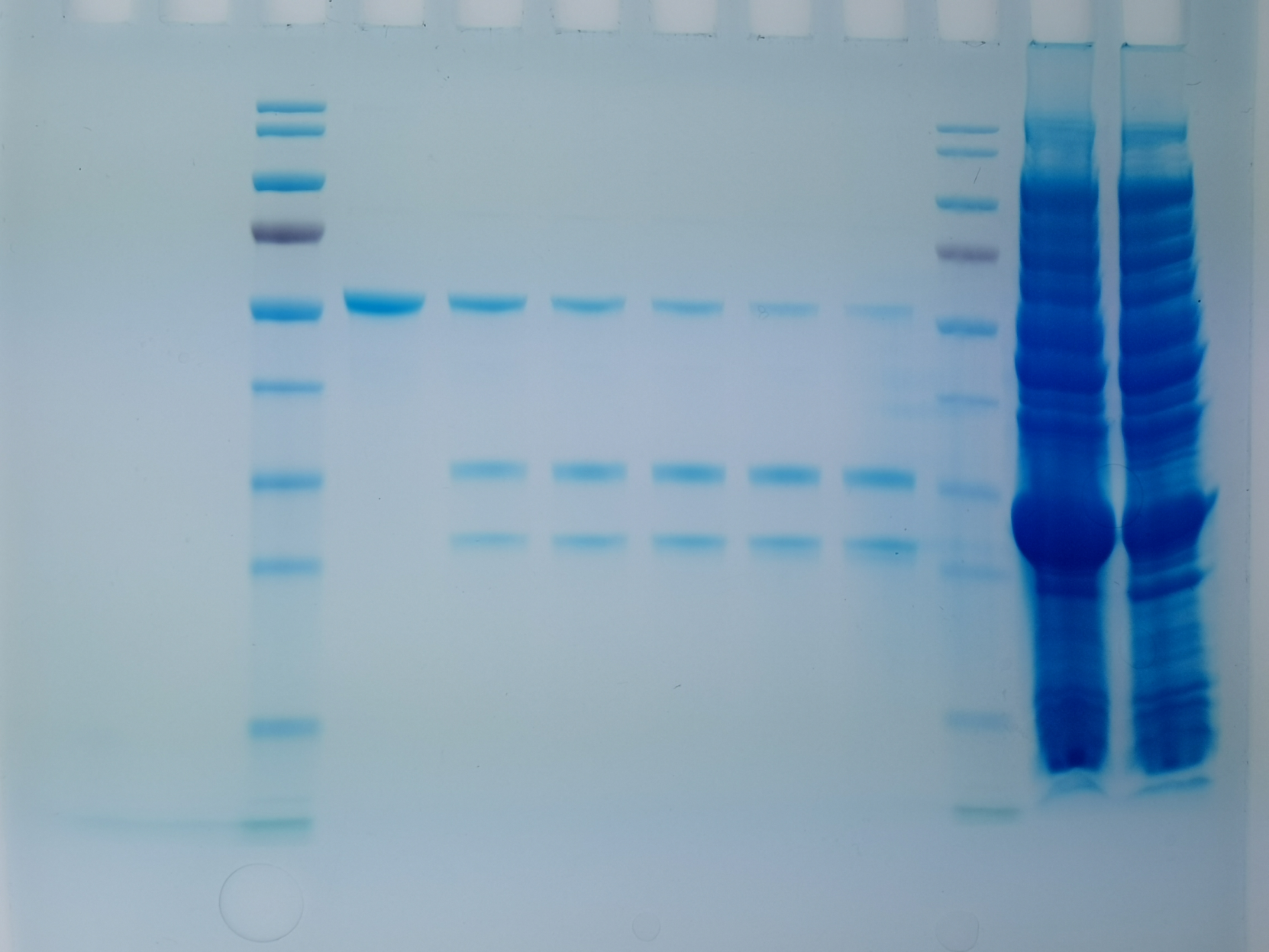

Supplement: Figure 1—source data 1. [file elife-89974-fig1-data1.zip › Figure 1-source data 1/Figure 1F-right-source date.tif]

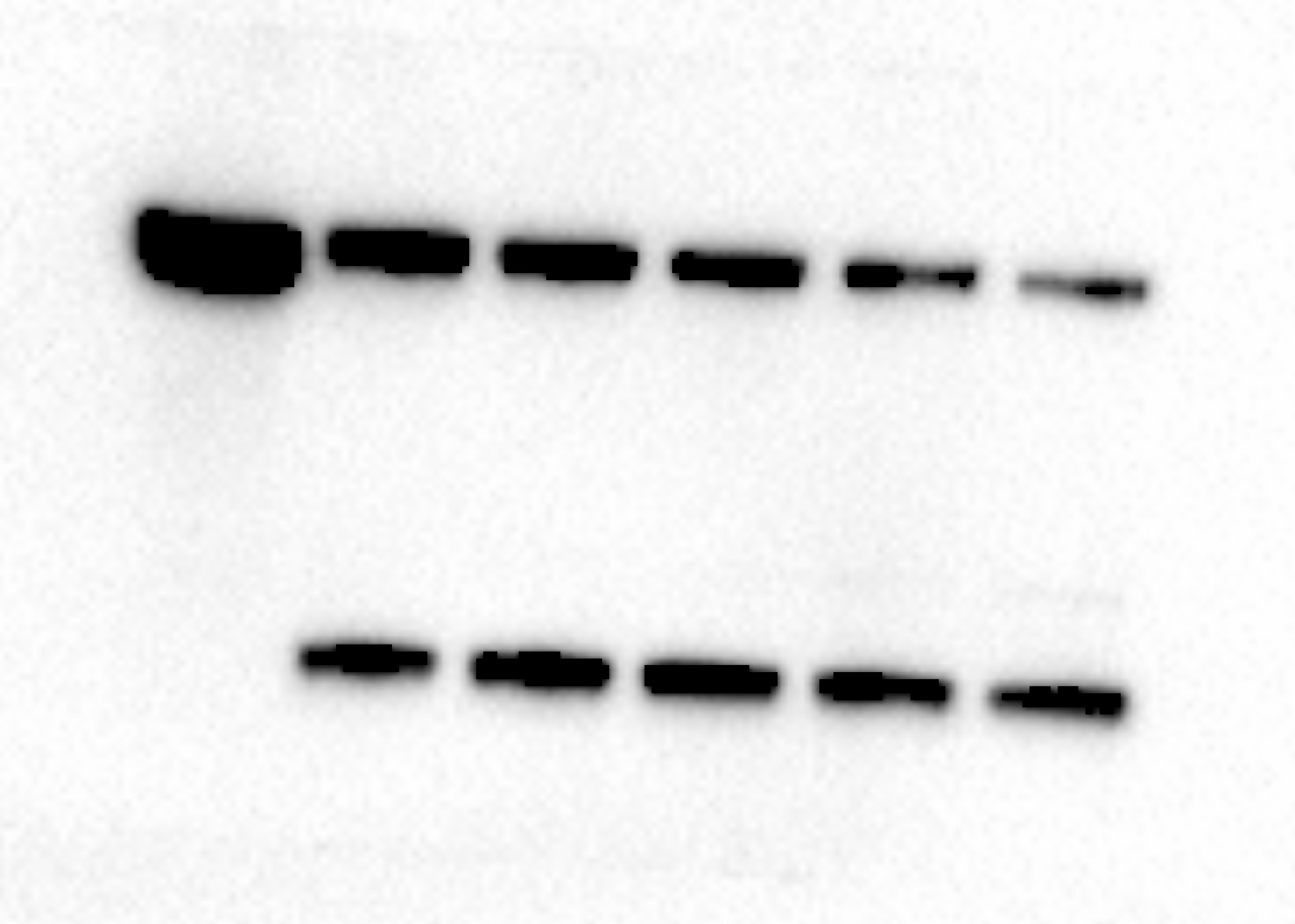

Supplement: Figure 1—source data 1. [file elife-89974-fig1-data1.zip › Figure 1-source data 1/Figure 1G-left-source data.tif]

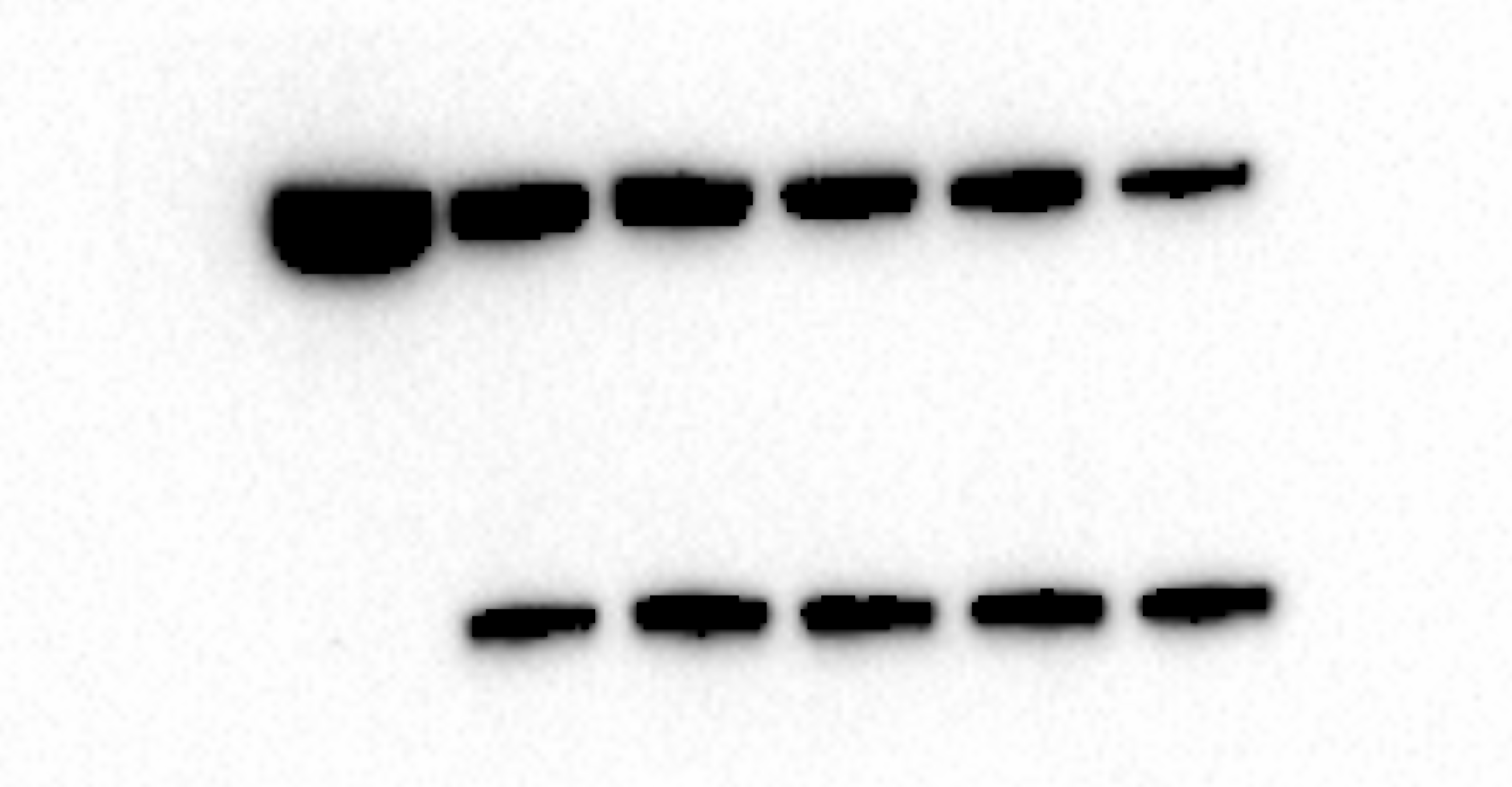

Supplement: Figure 1—source data 1. [file elife-89974-fig1-data1.zip › Figure 1-source data 1/Figure 1G-right-source data.tif]

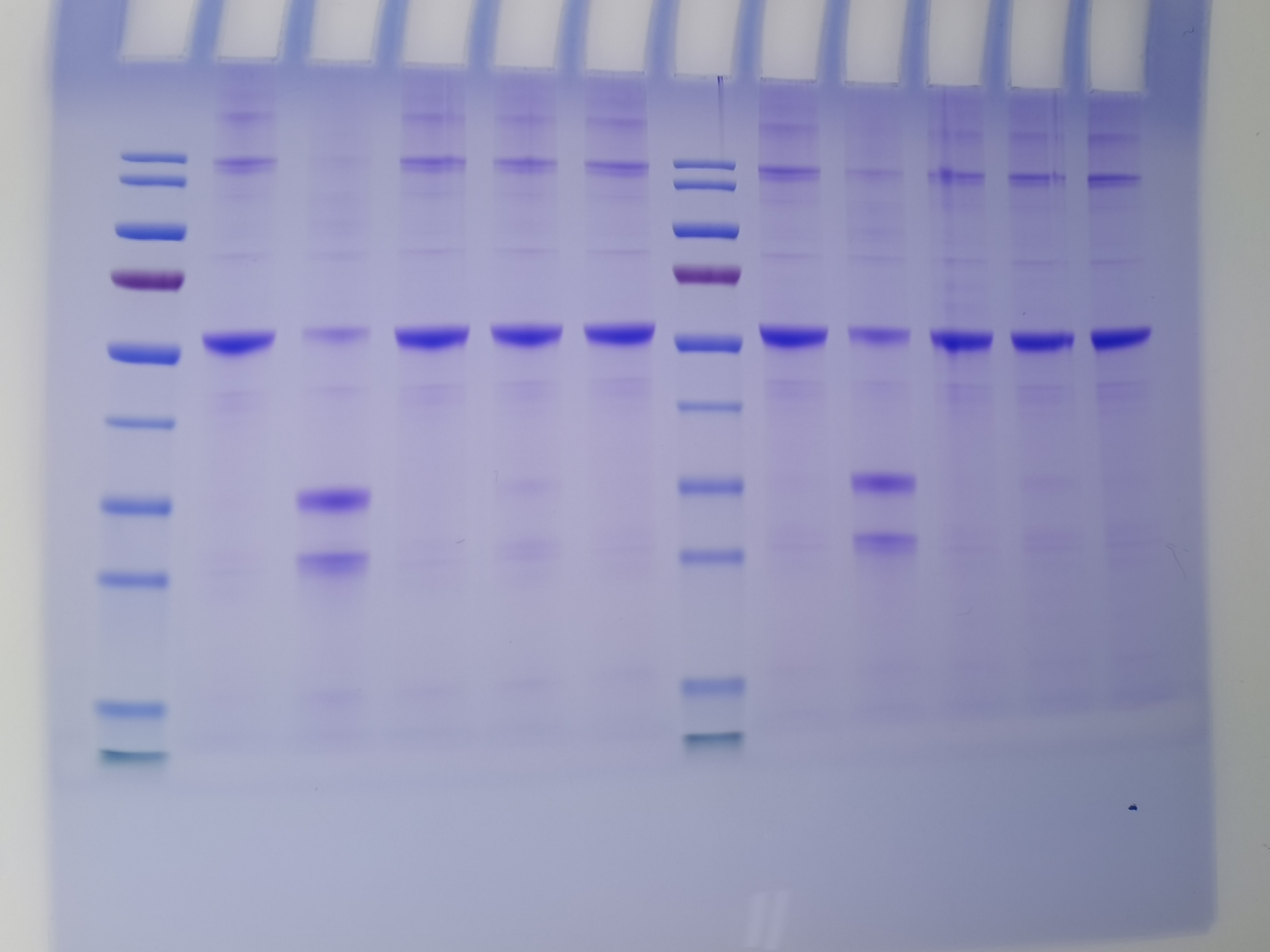

Supplement: Figure 1—source data 1. [file elife-89974-fig1-data1.zip › Figure 1-source data 1/Figure 1H-source data.tif]

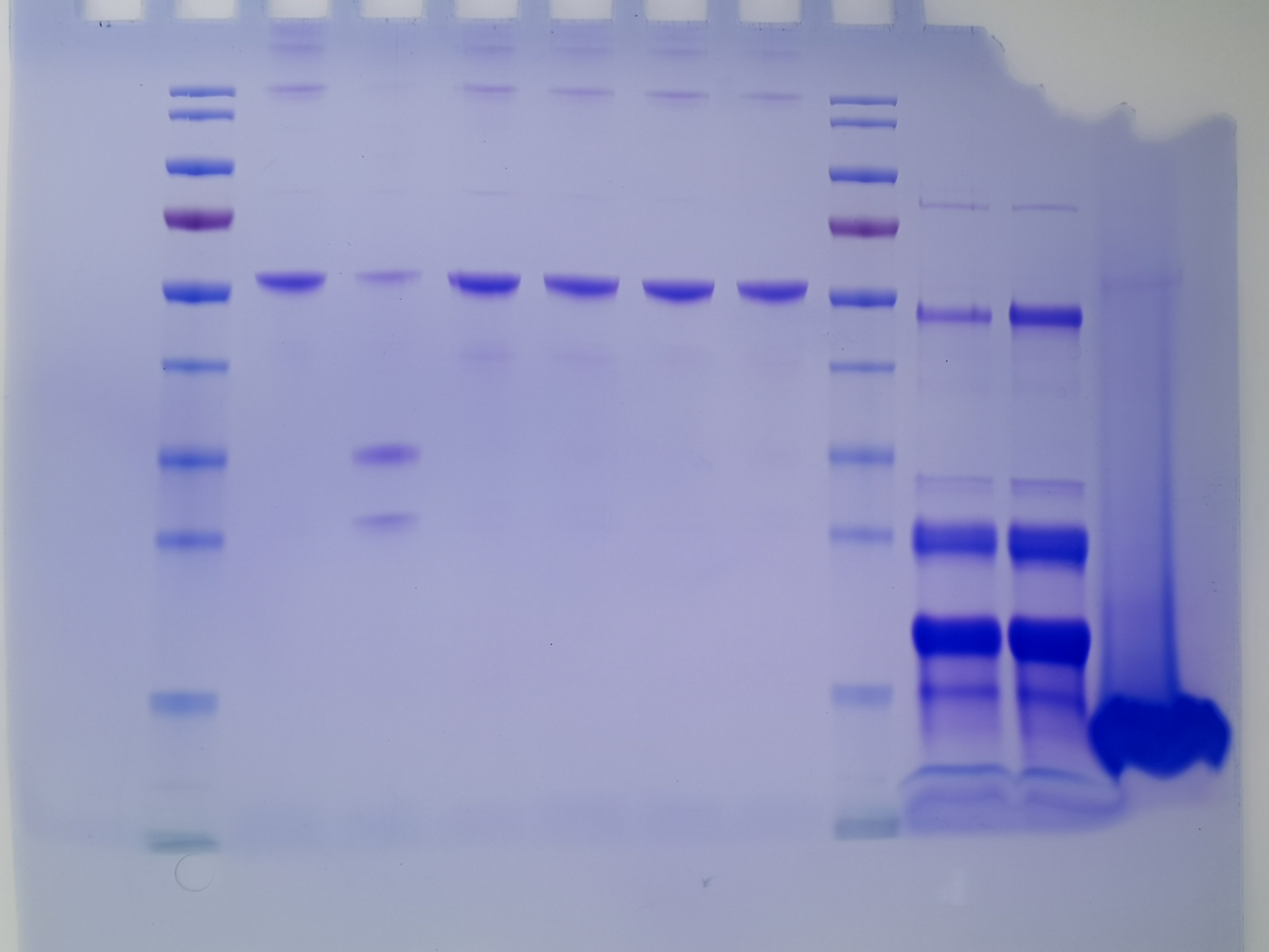

Supplement: Figure 1—source data 1. [file elife-89974-fig1-data1.zip › Figure 1-source data 1/Figure 1I-left-source data.tif]

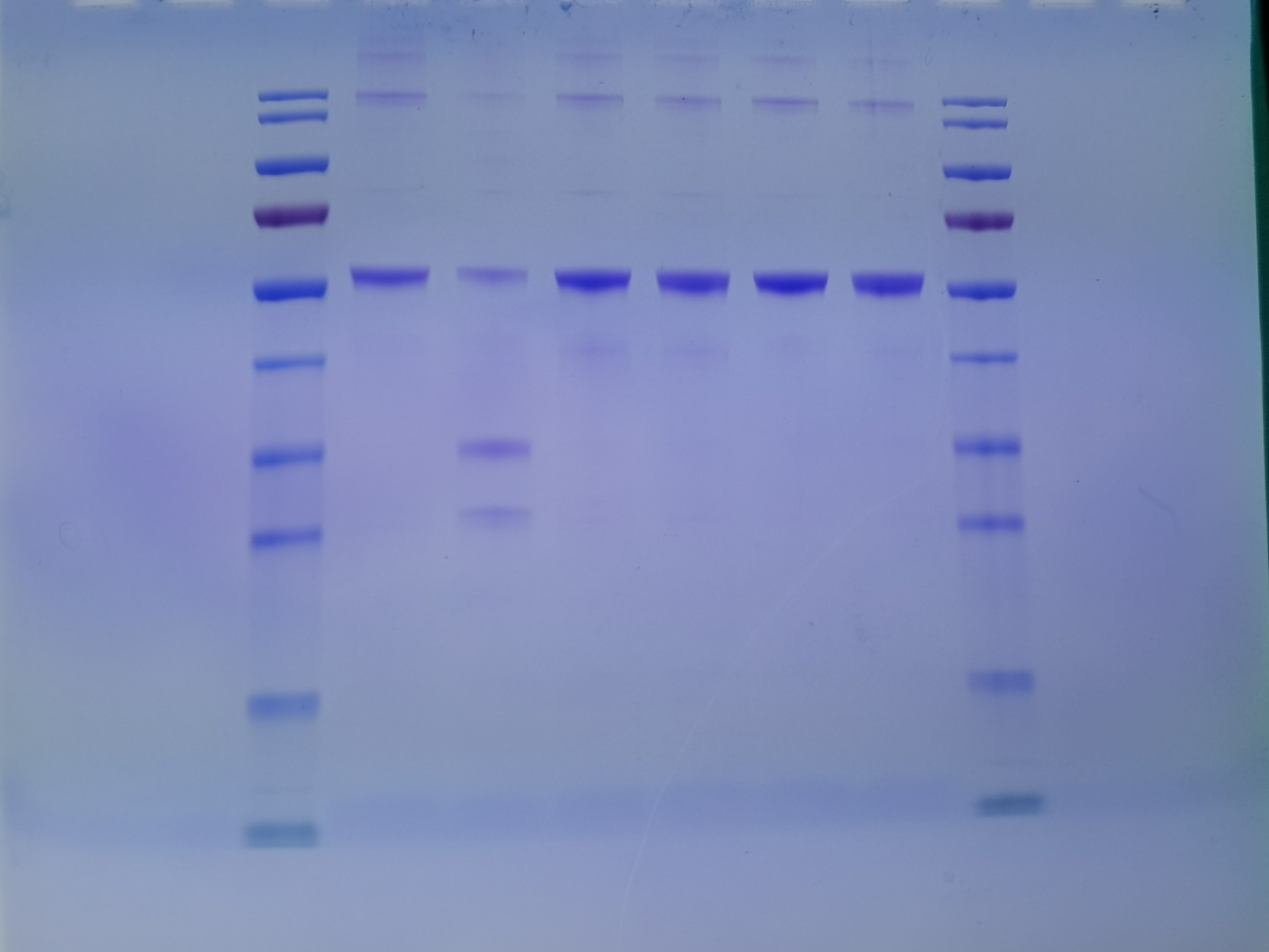

Supplement: Figure 1—source data 1. [file elife-89974-fig1-data1.zip › Figure 1-source data 1/Figure 1I-right-source data.tif]

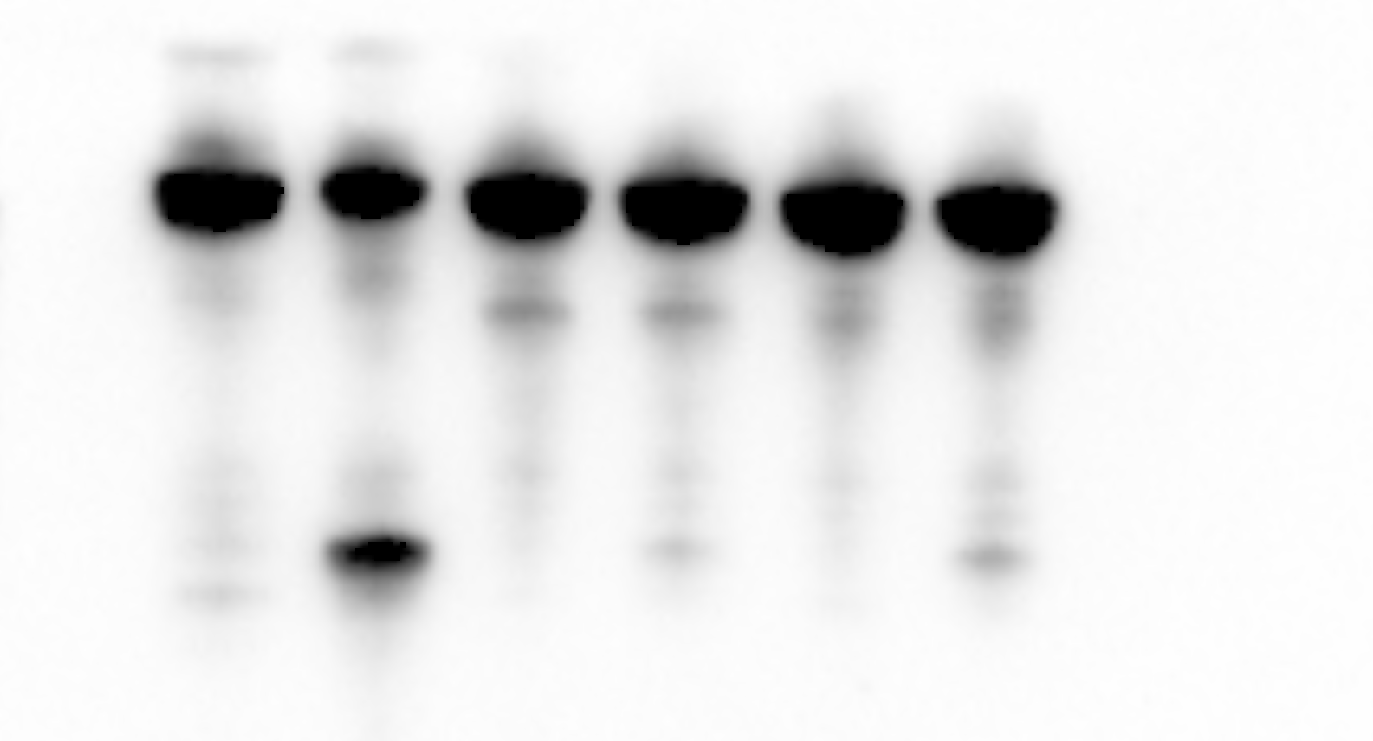

Supplement: Figure 1—source data 1. [file elife-89974-fig1-data1.zip › Figure 1-source data 1/Figure 1J-left-source data.tif]

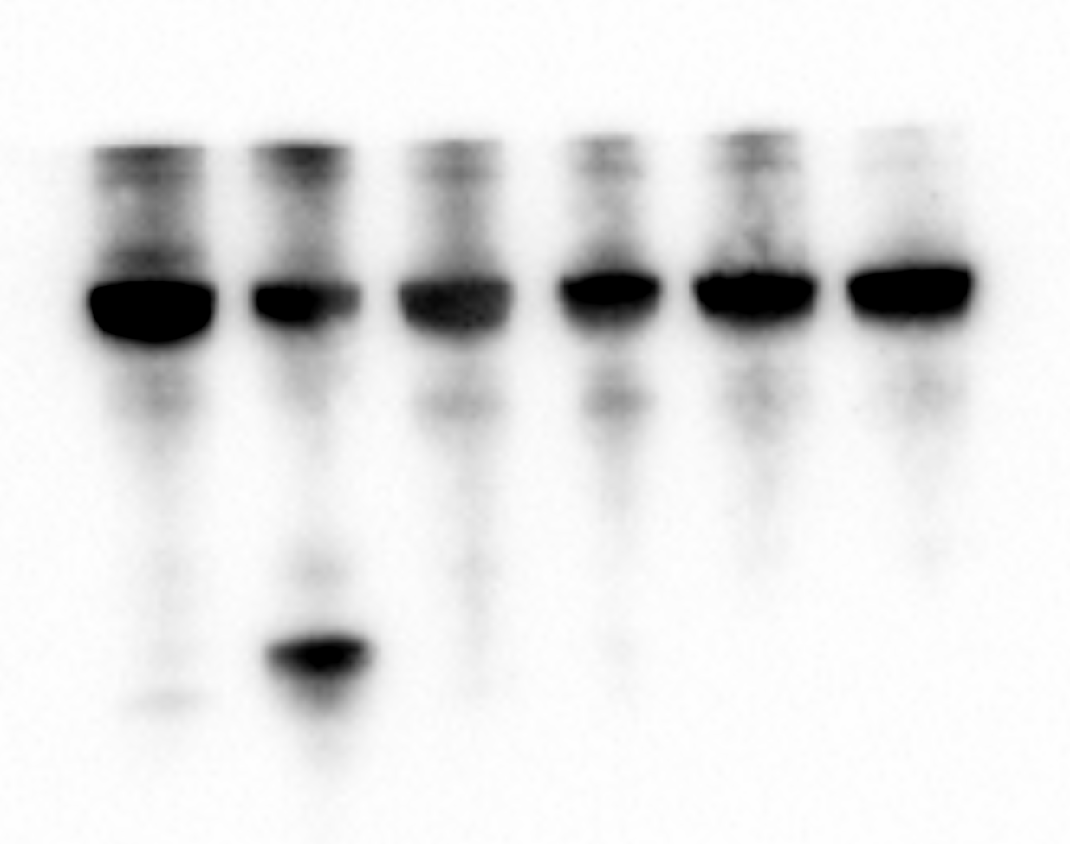

Supplement: Figure 1—source data 1. [file elife-89974-fig1-data1.zip › Figure 1-source data 1/Figure 1J-right-source data.tif]

**Figure 1A**

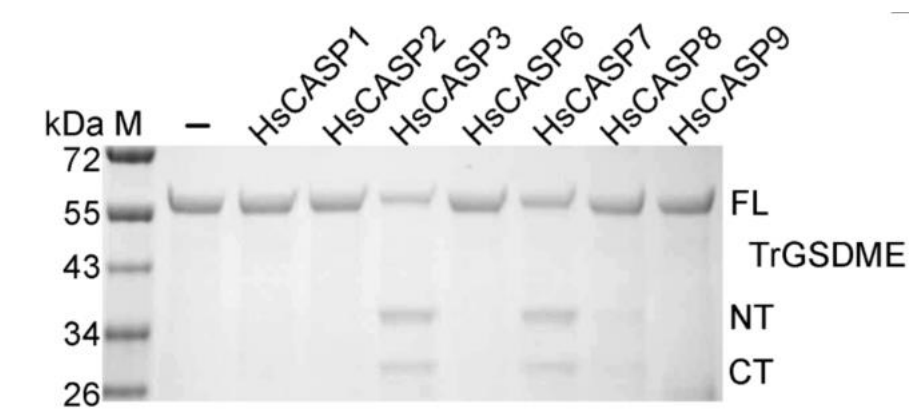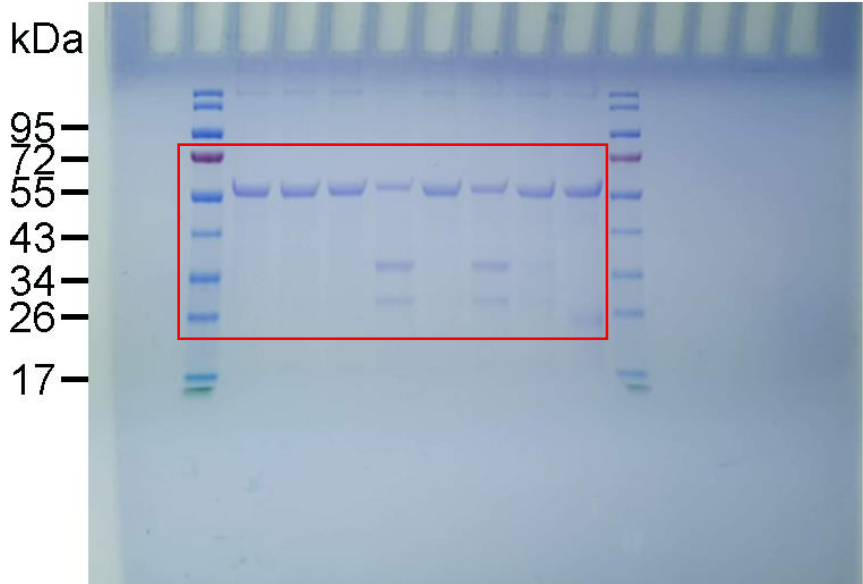

Supplement: Figure 1—source data 2. [file elife-89974-fig1-data2.zip › Figure 1-source data 2/Figure 1A-source data.pdf]

**Figure 1B**

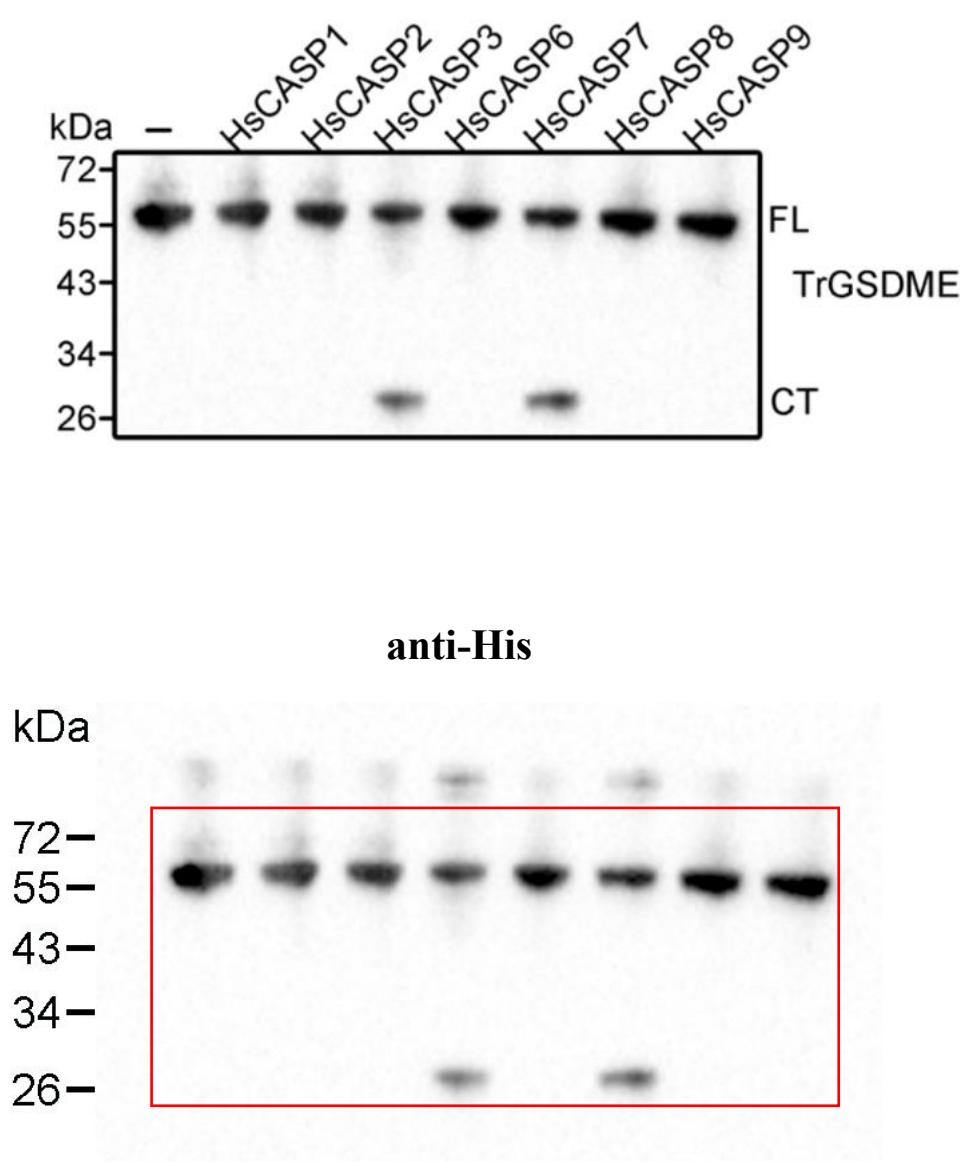

Supplement: Figure 1—source data 2. [file elife-89974-fig1-data2.zip › Figure 1-source data 2/Figure 1B-source data.pdf]

**Figure 1C**

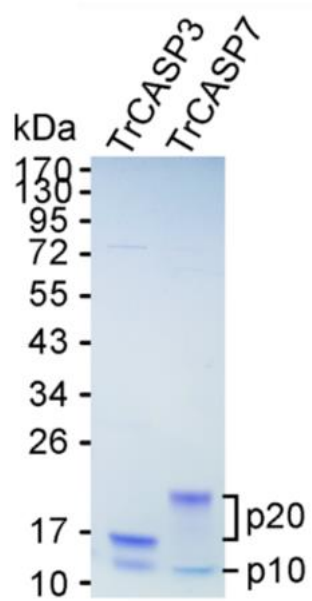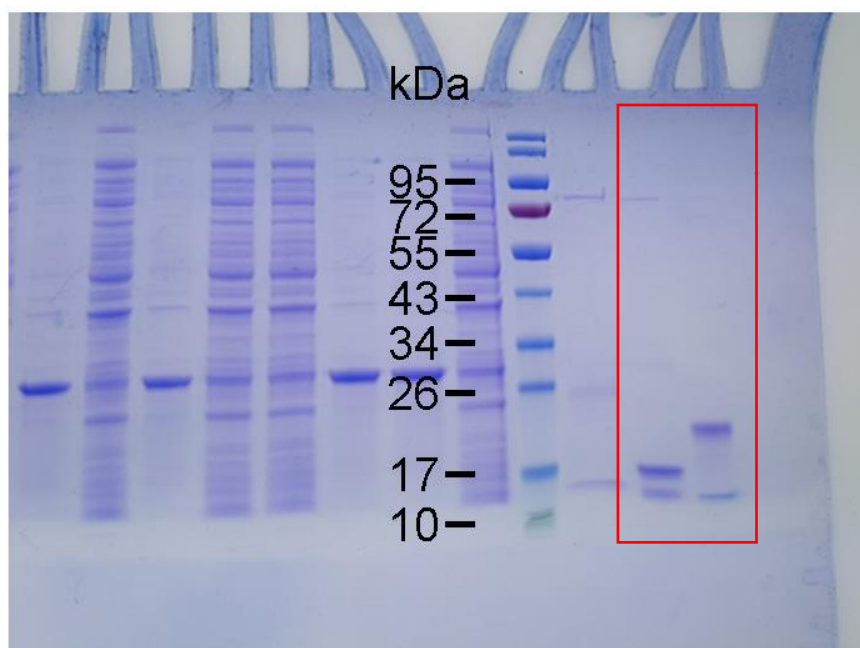

Supplement: Figure 1—source data 2. [file elife-89974-fig1-data2.zip › Figure 1-source data 2/Figure 1C-source data.pdf]

**Figure 1F-left**

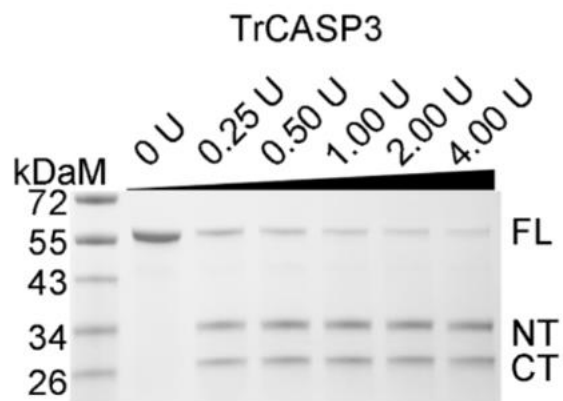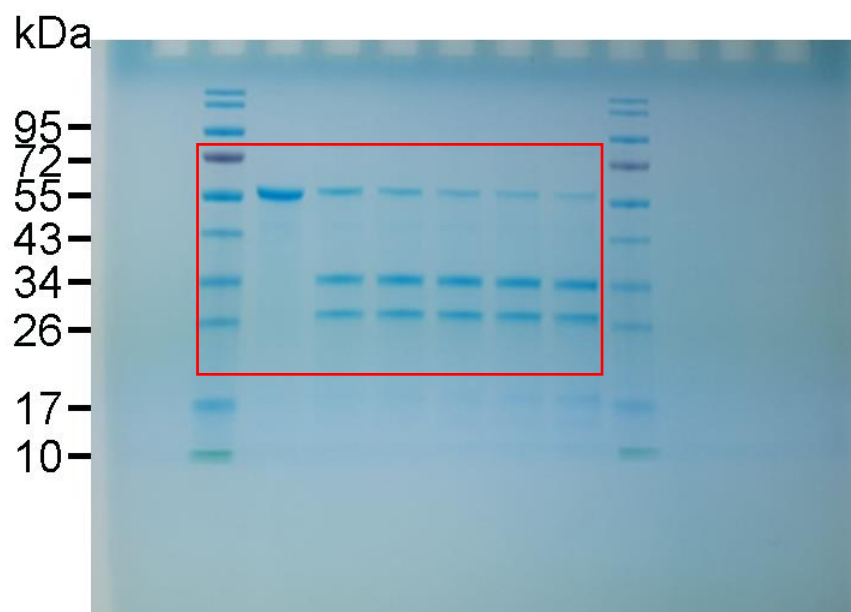

Supplement: Figure 1—source data 2. [file elife-89974-fig1-data2.zip › Figure 1-source data 2/Figure 1F-left-source data.pdf]

**Figure 1F-right**

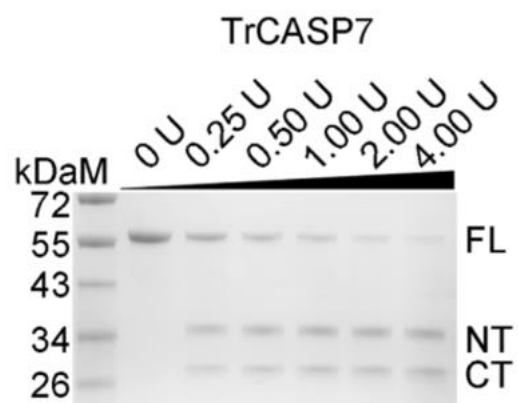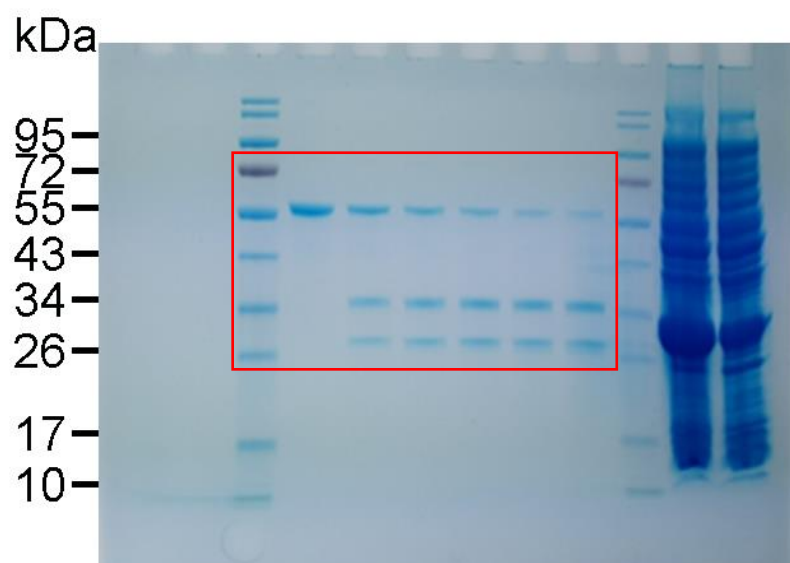

Supplement: Figure 1—source data 2. [file elife-89974-fig1-data2.zip › Figure 1-source data 2/Figure 1F-right-source data.pdf]

**Figure 1G-left**

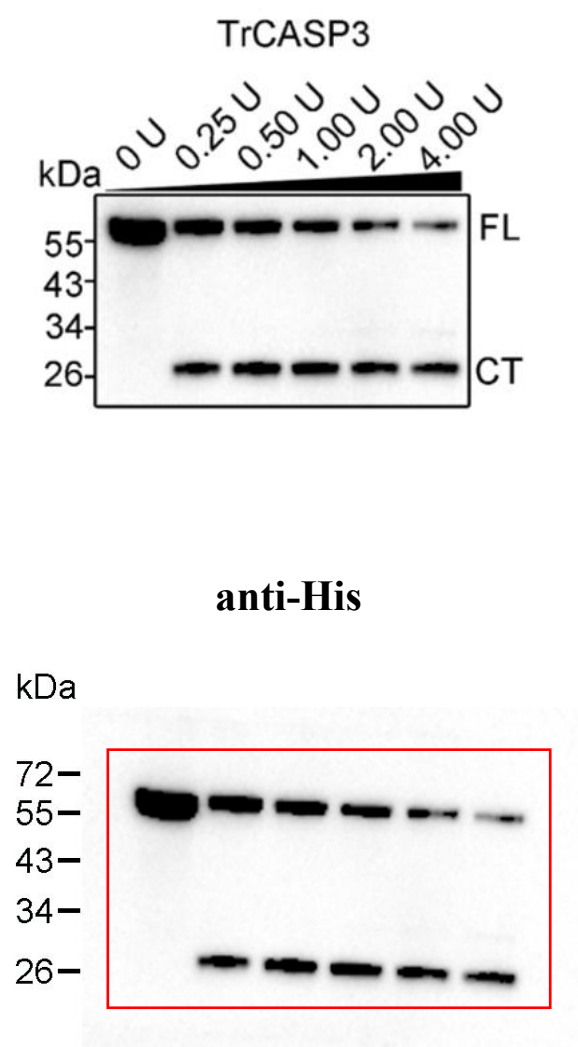

Supplement: Figure 1—source data 2. [file elife-89974-fig1-data2.zip › Figure 1-source data 2/Figure 1G-left-source data.pdf]

**Figure 1G-right**

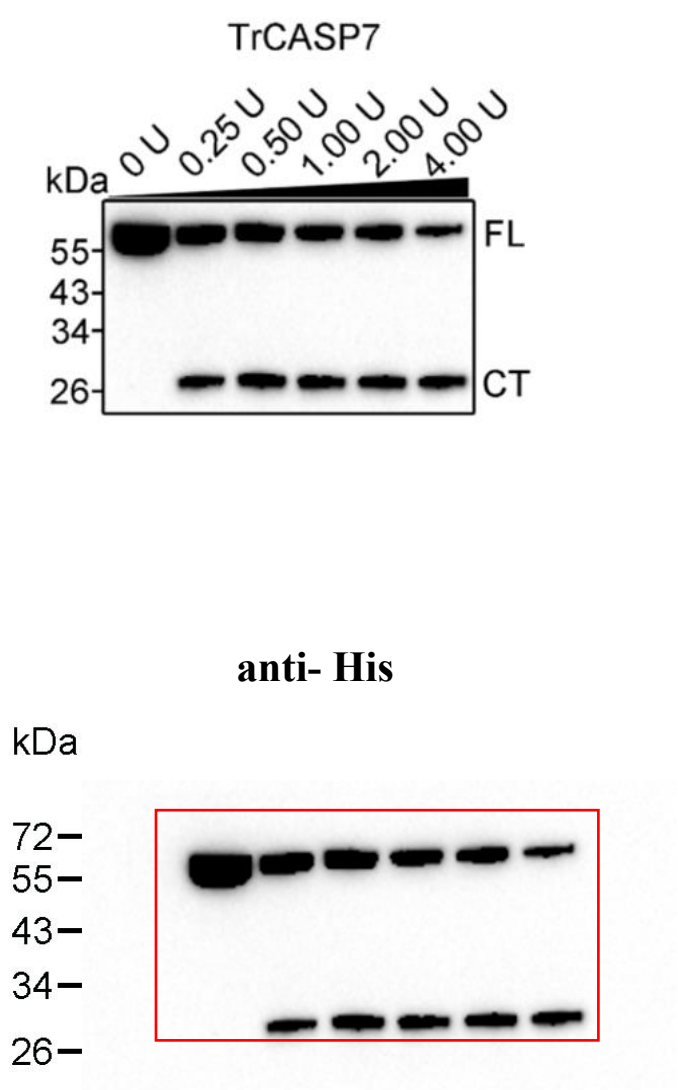

Supplement: Figure 1—source data 2. [file elife-89974-fig1-data2.zip › Figure 1-source data 2/Figure 1G-right-source data.pdf]

Figure 1H

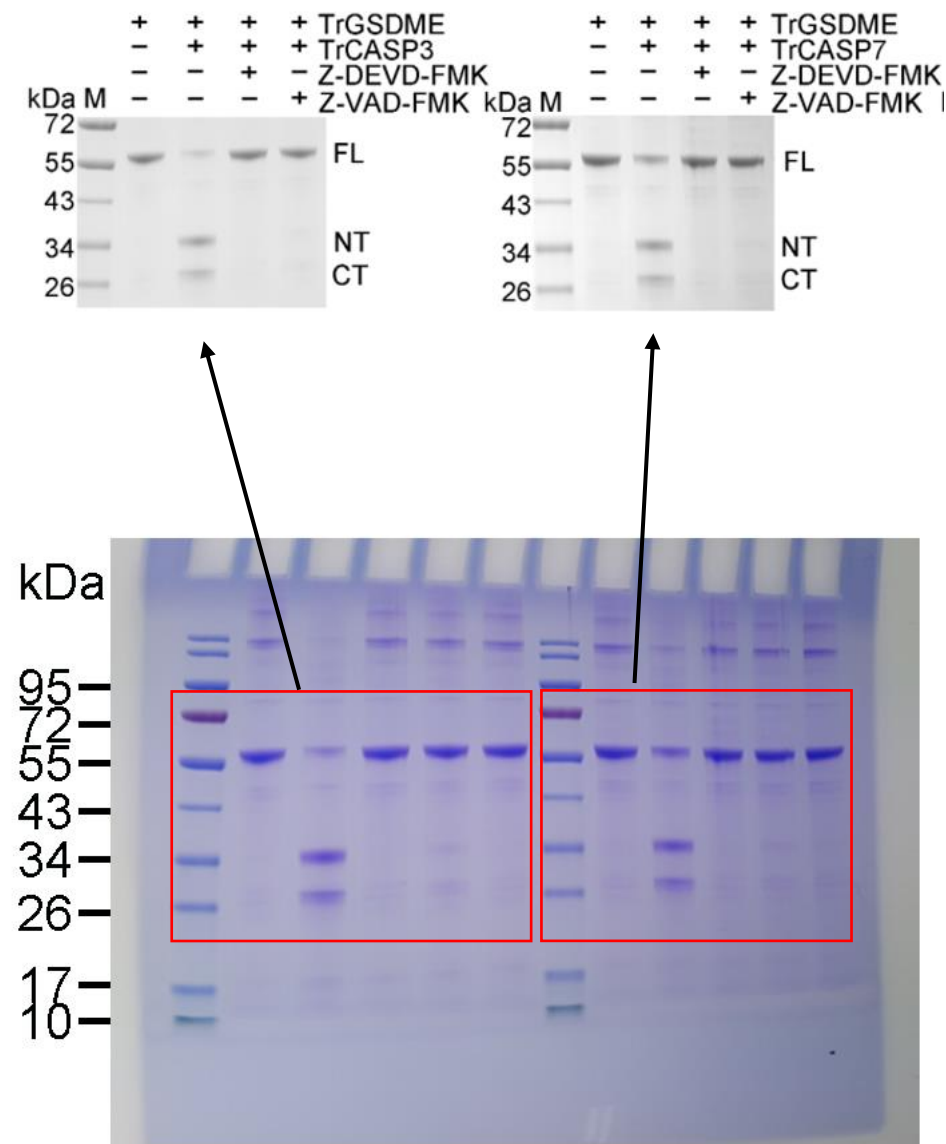

Supplement: Figure 1—source data 2. [file elife-89974-fig1-data2.zip › Figure 1-source data 2/Figure 1H-source data.pdf]

**Figure 1I-left**

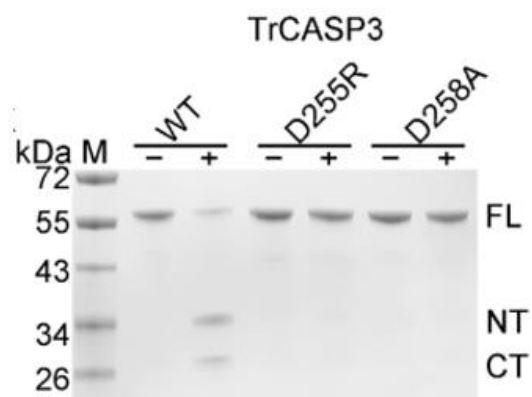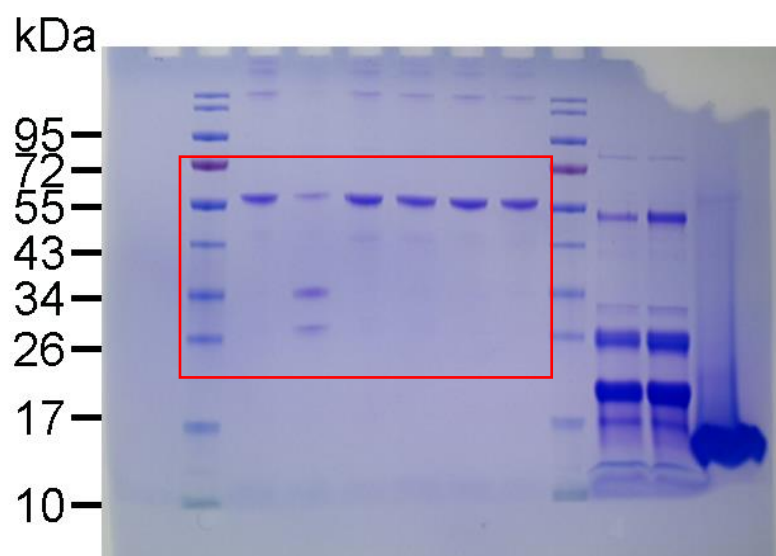

Supplement: Figure 1—source data 2. [file elife-89974-fig1-data2.zip › Figure 1-source data 2/Figure 1I-left-source data.pdf]

Figure 1I-right

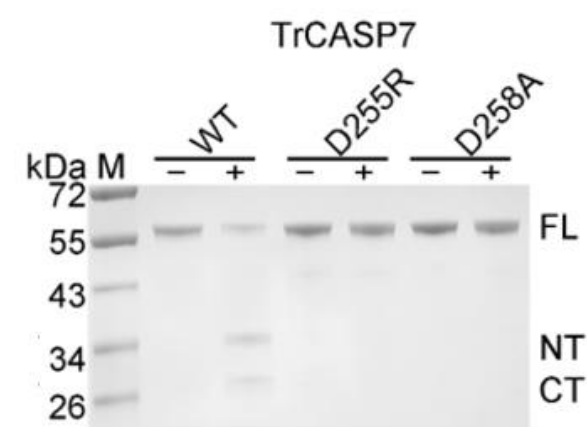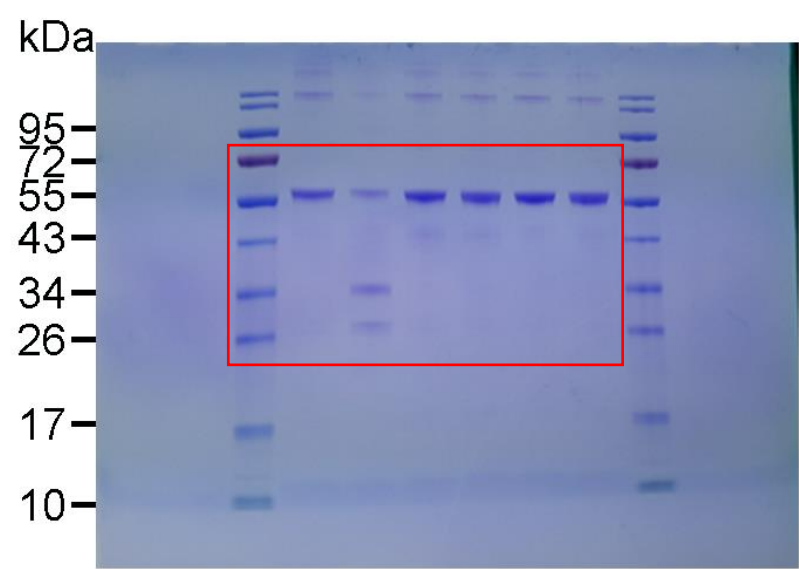

Supplement: Figure 1—source data 2. [file elife-89974-fig1-data2.zip › Figure 1-source data 2/Figure 1I-right-source data.pdf]

**Figure 1J-left**

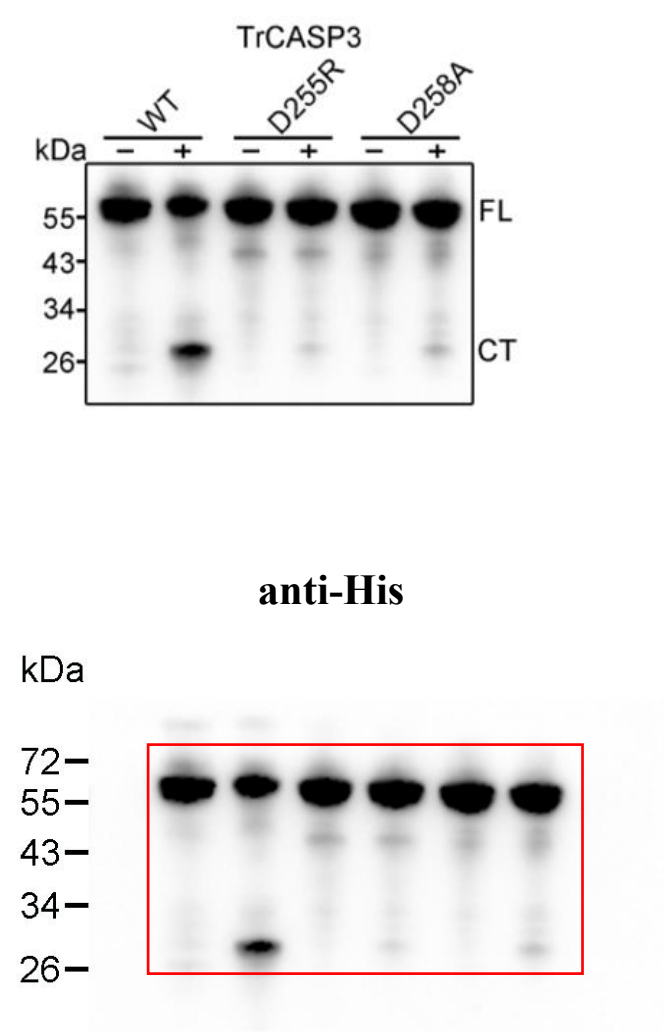

Supplement: Figure 1—source data 2. [file elife-89974-fig1-data2.zip › Figure 1-source data 2/Figure 1J-left-source data.pdf]

**Figure 1J-right**

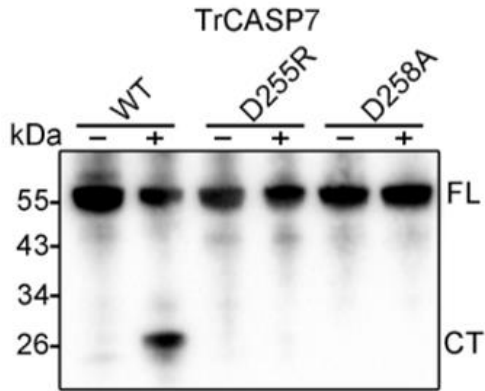

**anti-His**

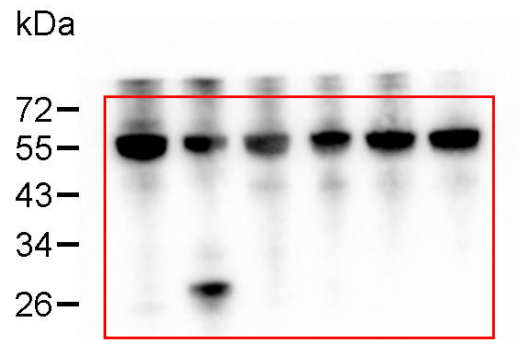

Supplement: Figure 1—source data 2. [file elife-89974-fig1-data2.zip › Figure 1-source data 2/Figure 1J-right-source data.pdf]

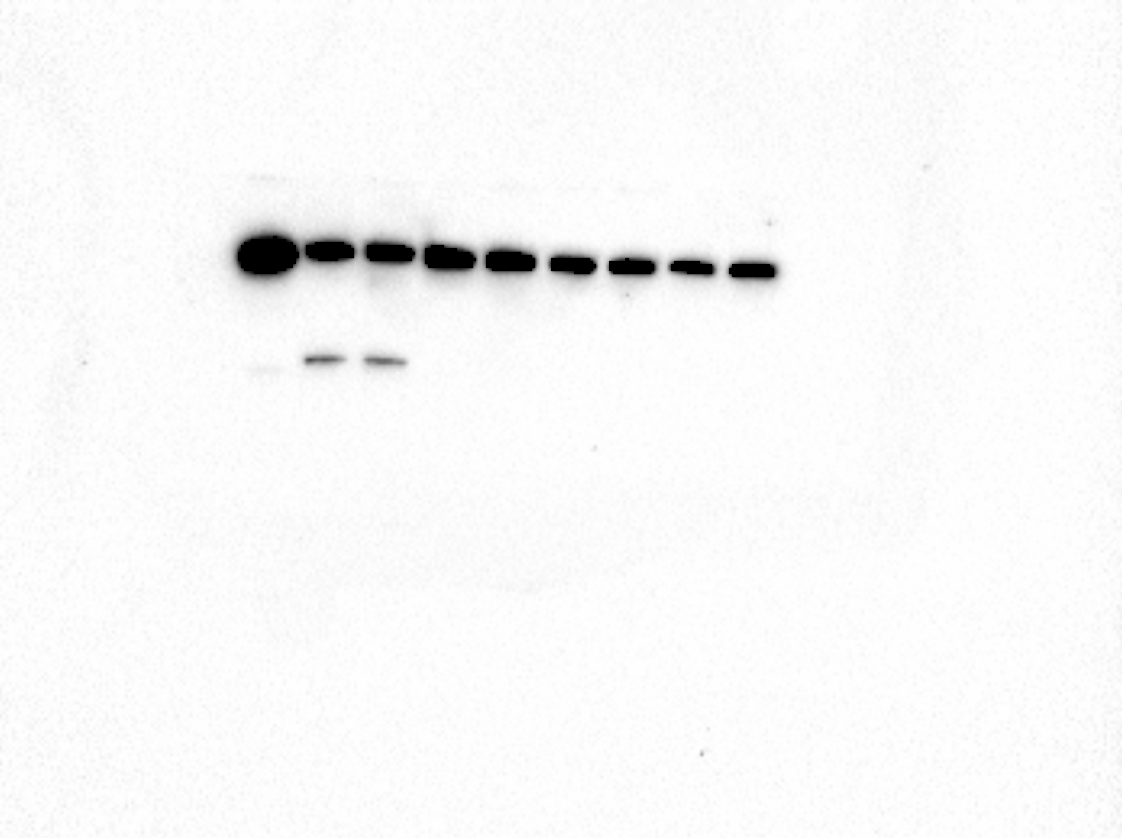

Supplement: Figure 1—figure supplement 2—source data 1. [file elife-89974-fig1-figsupp2-data1.zip › Figure 1-figure supplement 2-source data 1/Figure 1-figure supplement 2-source data.tif]

**Figure 1-figure supplement 2**

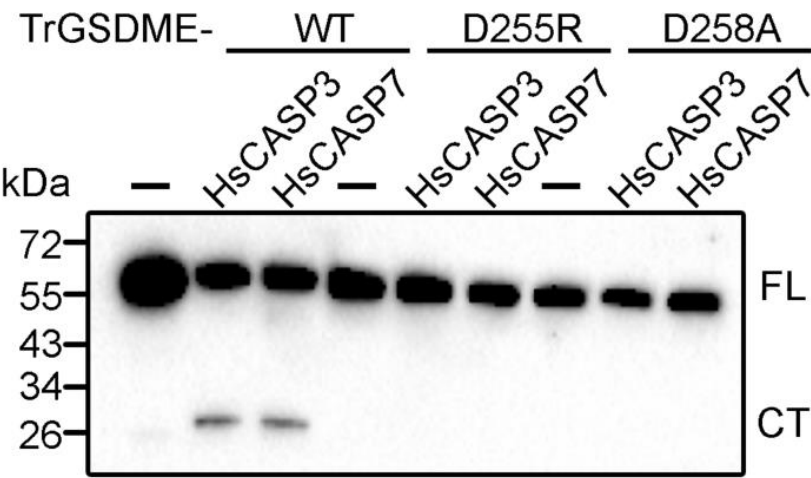

**anti-His**

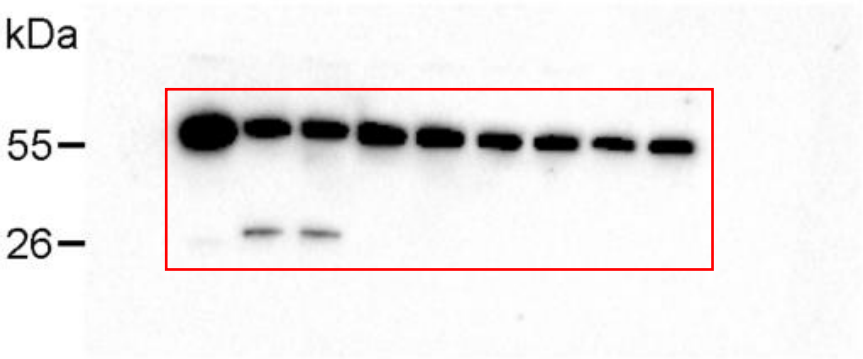

Supplement: Figure 1—figure supplement 2—source data 2. [file elife-89974-fig1-figsupp2-data2.zip › Figure 1-figure supplement 2-source data 2/Figure 1-figure supplement 2-source data.pdf]

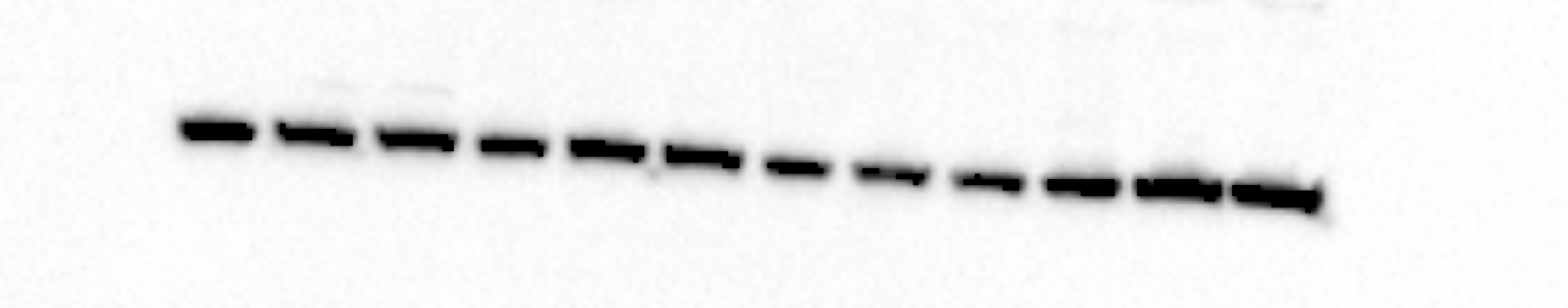

Supplement: Figure 2—source data 1. [file elife-89974-fig2-data1.zip › Figure 2-source data 1/Figure 2I-source data (anti-actin).tif]

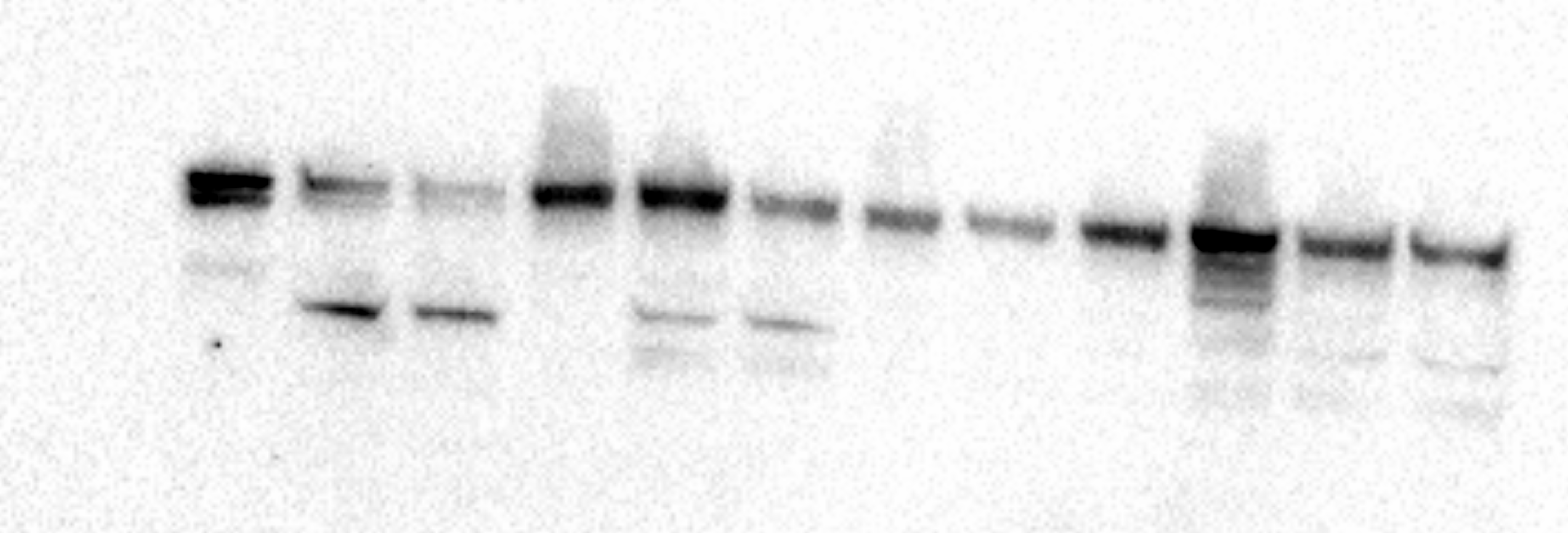

Supplement: Figure 2—source data 1. [file elife-89974-fig2-data1.zip › Figure 2-source data 1/Figure 2I-source data (anti-mcherry).tif]

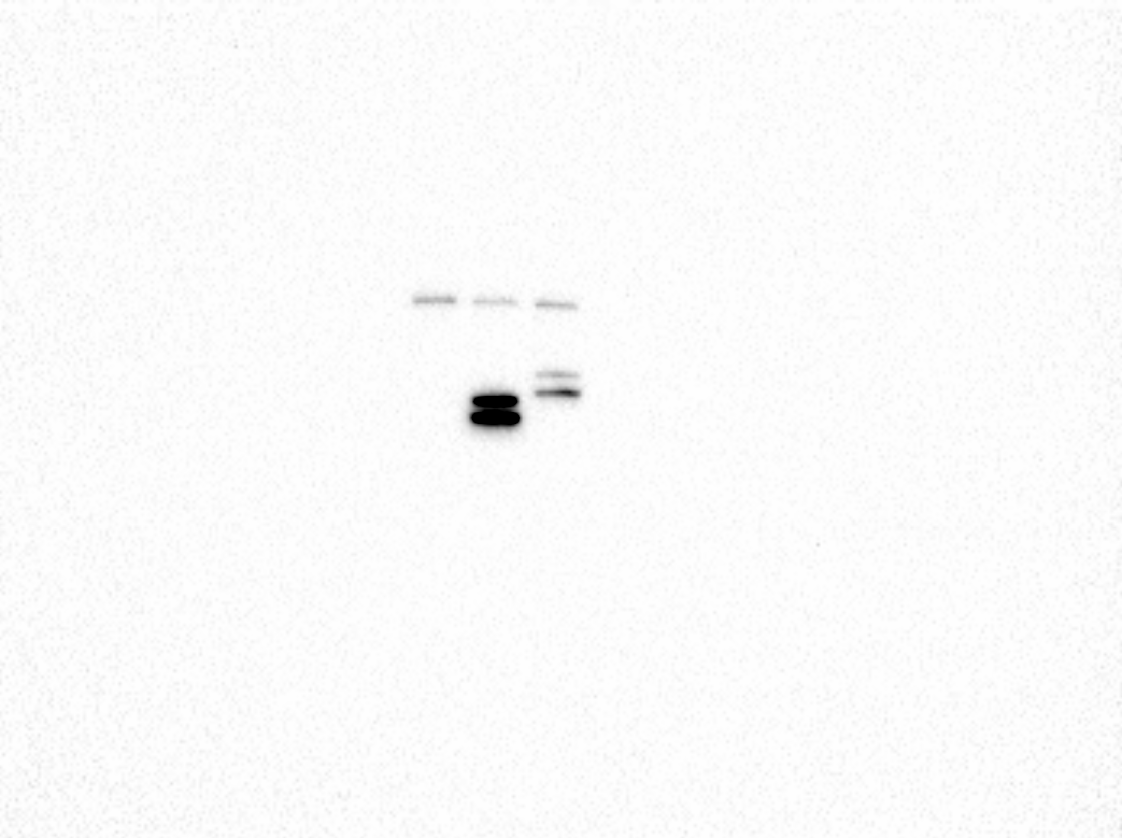

Supplement: Figure 2—source data 1. [file elife-89974-fig2-data1.zip › Figure 2-source data 1/Figure 2I-source data (anti-myc).tif]

**Figure 2I**

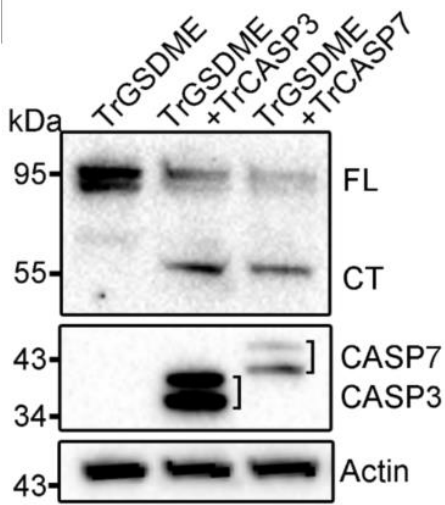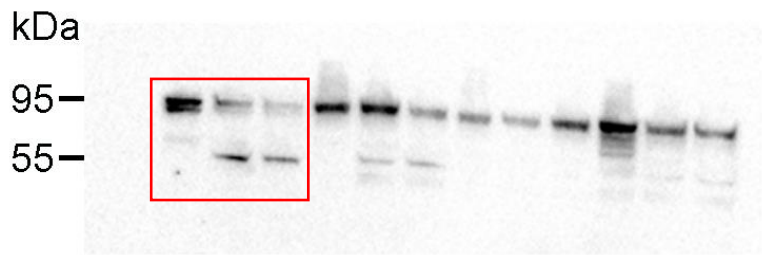

**anti-mcherry**

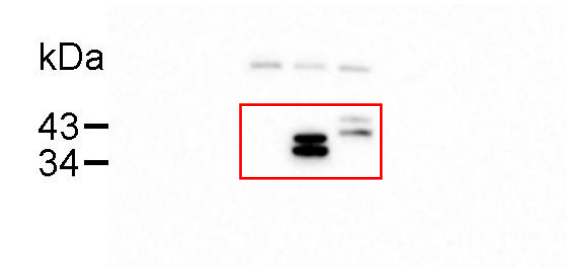

**anti-myc**

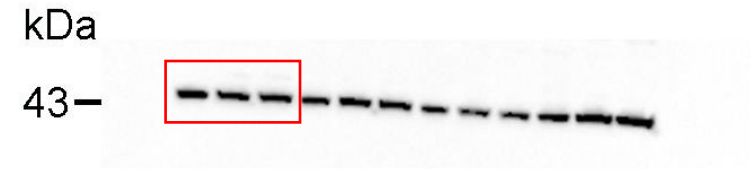

**anti-actin**

Supplement: Figure 2—source data 2. [file elife-89974-fig2-data2.zip › Figure 2-source data 2/Figure 2I-source data.pdf]

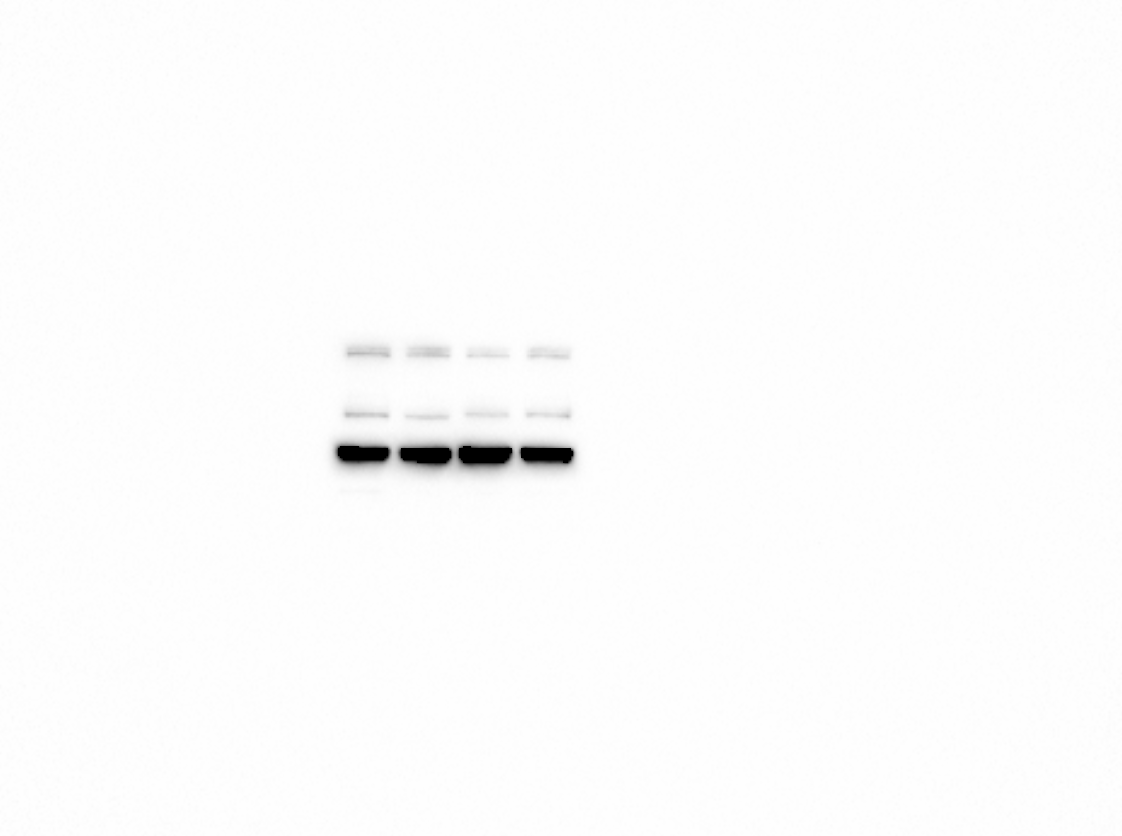

Supplement: Figure 2—figure supplement 1—source data 1. [file elife-89974-fig2-figsupp1-data1.zip › Figure 2-figure supplement 1-source data 1/Figure 2-figure supplement 1-source data (anti-actin).tif]

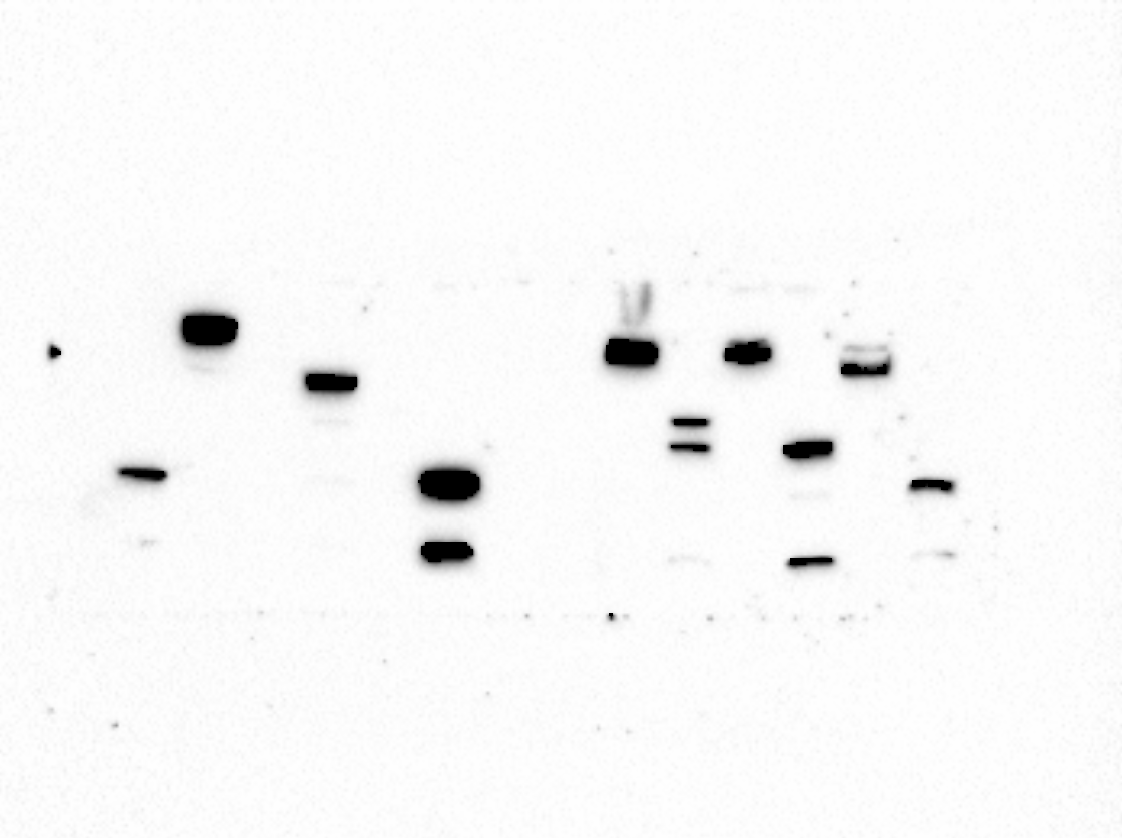

Supplement: Figure 2—figure supplement 1—source data 1. [file elife-89974-fig2-figsupp1-data1.zip › Figure 2-figure supplement 1-source data 1/Figure 2-figure supplement 1-source data (anti-mcherry).tif]

**Figure 2-figure supplement 1**

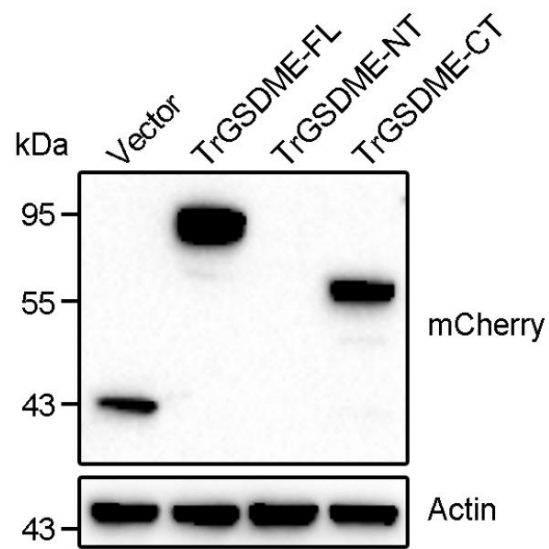

**anti-mcherry**

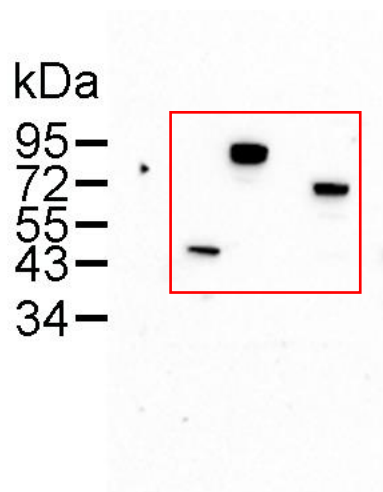

**anti-actin**

kDa

43—

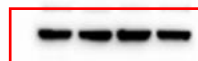

Supplement: Figure 2—figure supplement 1—source data 2. [file elife-89974-fig2-figsupp1-data2.zip › Figure 2-figure supplement 1-source data 2/Figure 2-figure supplement 1-source data.pdf]

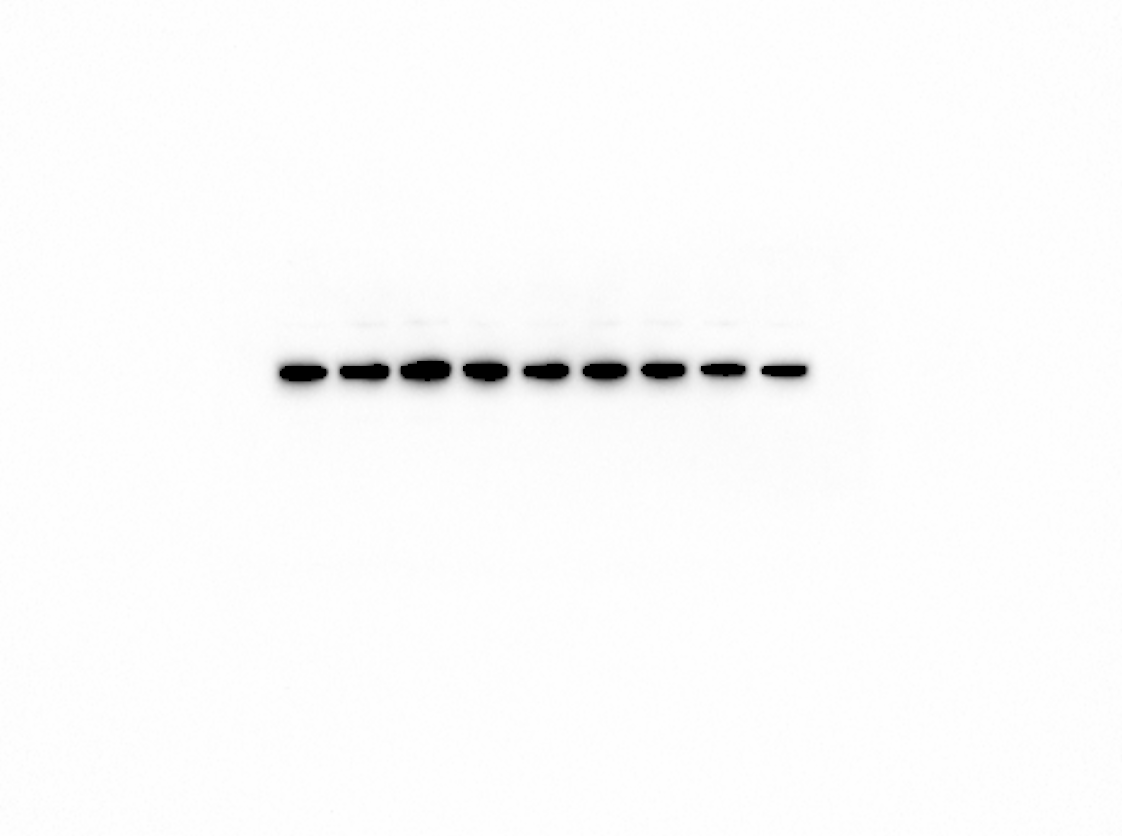

Supplement: Figure 2—figure supplement 3—source data 1. [file elife-89974-fig2-figsupp3-data1.zip › Figure 2-figure supplement 3-source data 1/Figure 2-figure supplement 3C-source data (anti-actin).tif]

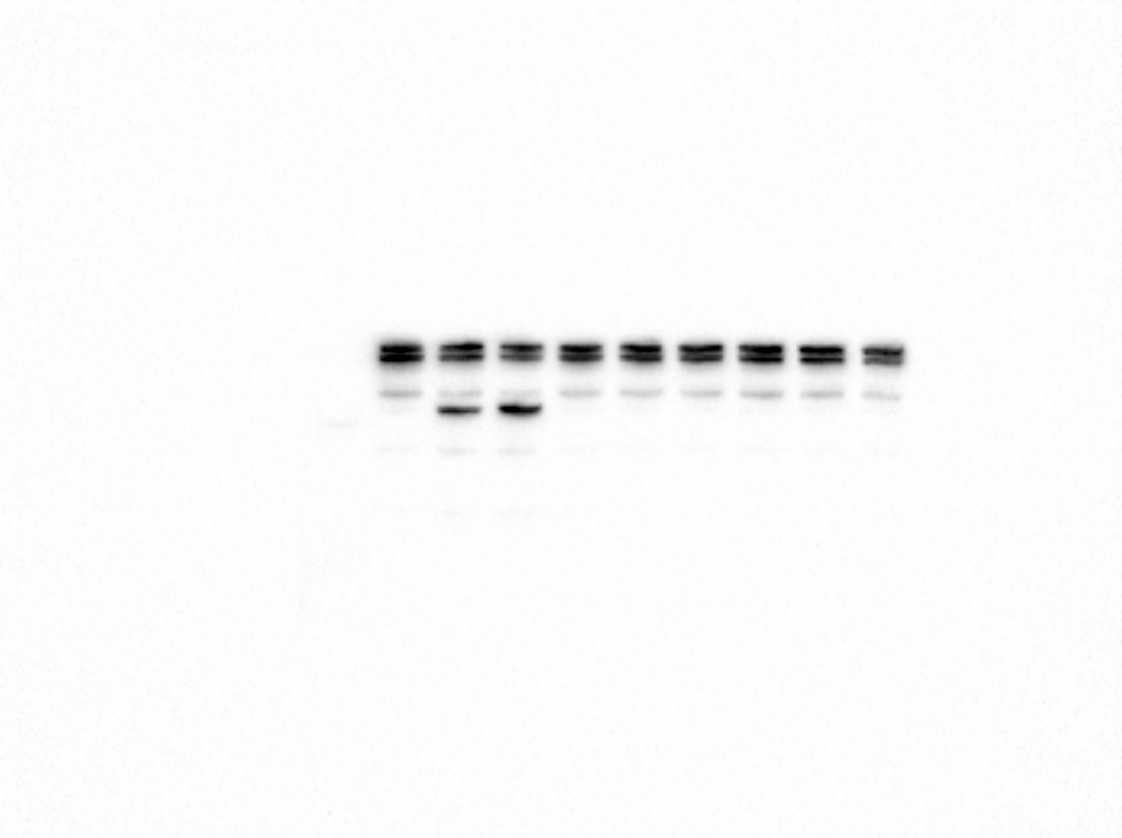

Supplement: Figure 2—figure supplement 3—source data 1. [file elife-89974-fig2-figsupp3-data1.zip › Figure 2-figure supplement 3-source data 1/Figure 2-figure supplement 3C-source data (anti-mcheryy).tif]

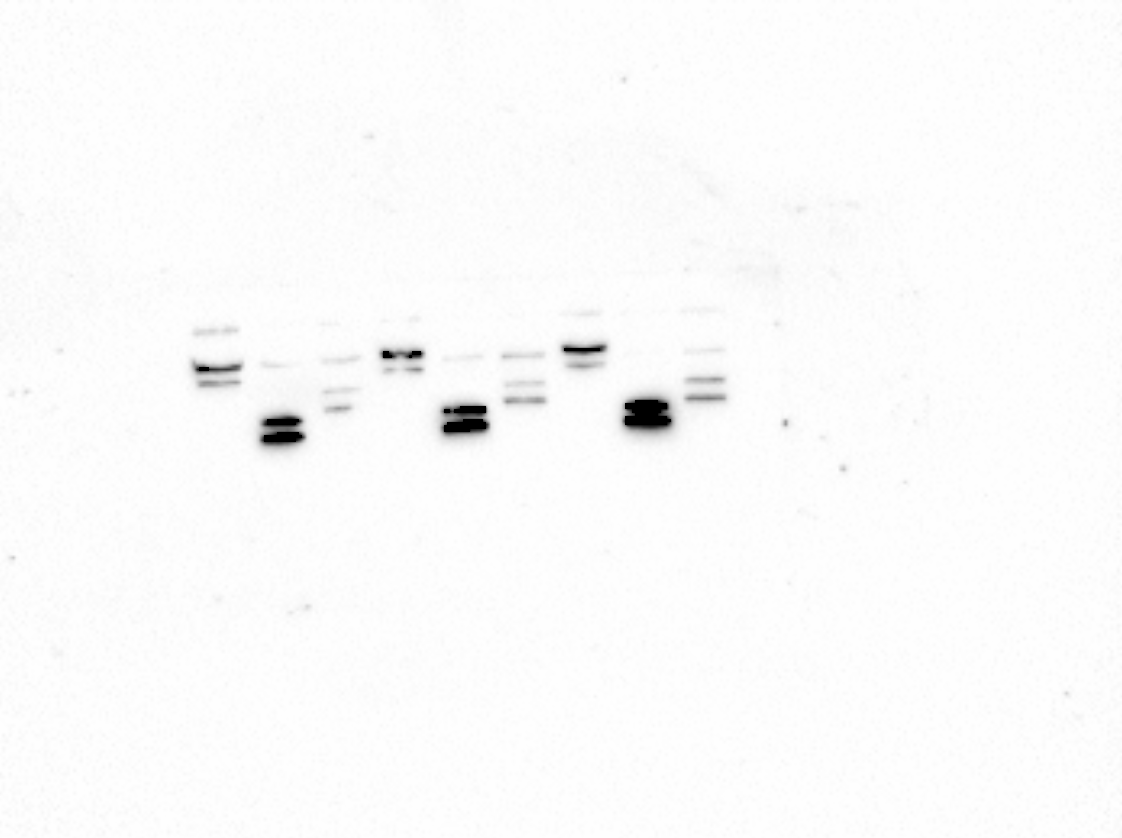

Supplement: Figure 2—figure supplement 3—source data 1. [file elife-89974-fig2-figsupp3-data1.zip › Figure 2-figure supplement 3-source data 1/Figure 2-figure supplement 3C-source data (anti-myc).tif]

**Figure 2-figure supplement 3C**

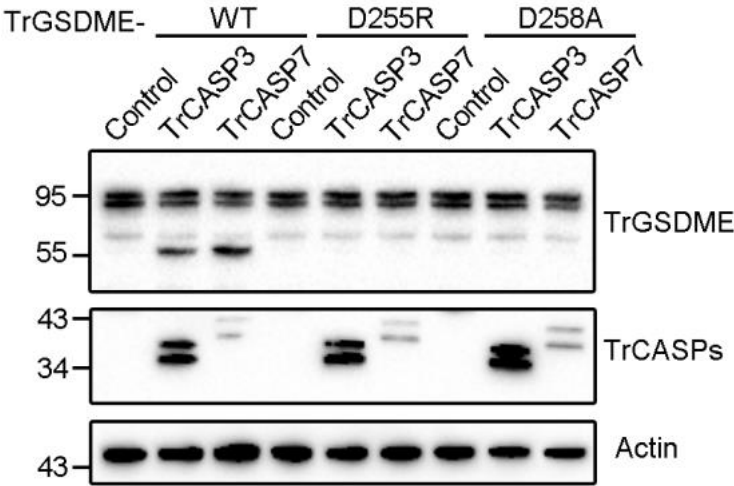

**anti-mCherry**

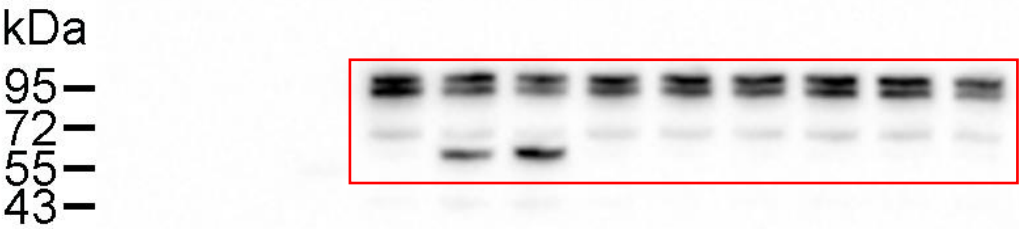

**anti-Myc**

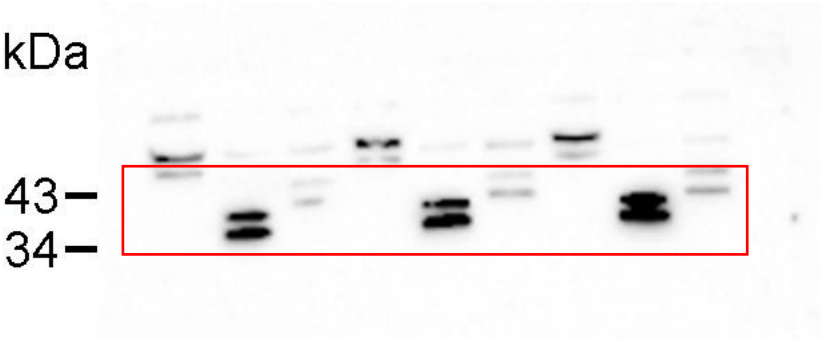

**anti-actin**

kDa

43—

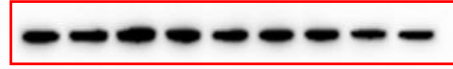

Supplement: Figure 2—figure supplement 3—source data 2. [file elife-89974-fig2-figsupp3-data2.zip › Figure 2-figure supplement 3-source data 2/Figure 2-figure supplement 3C-source data.pdf]

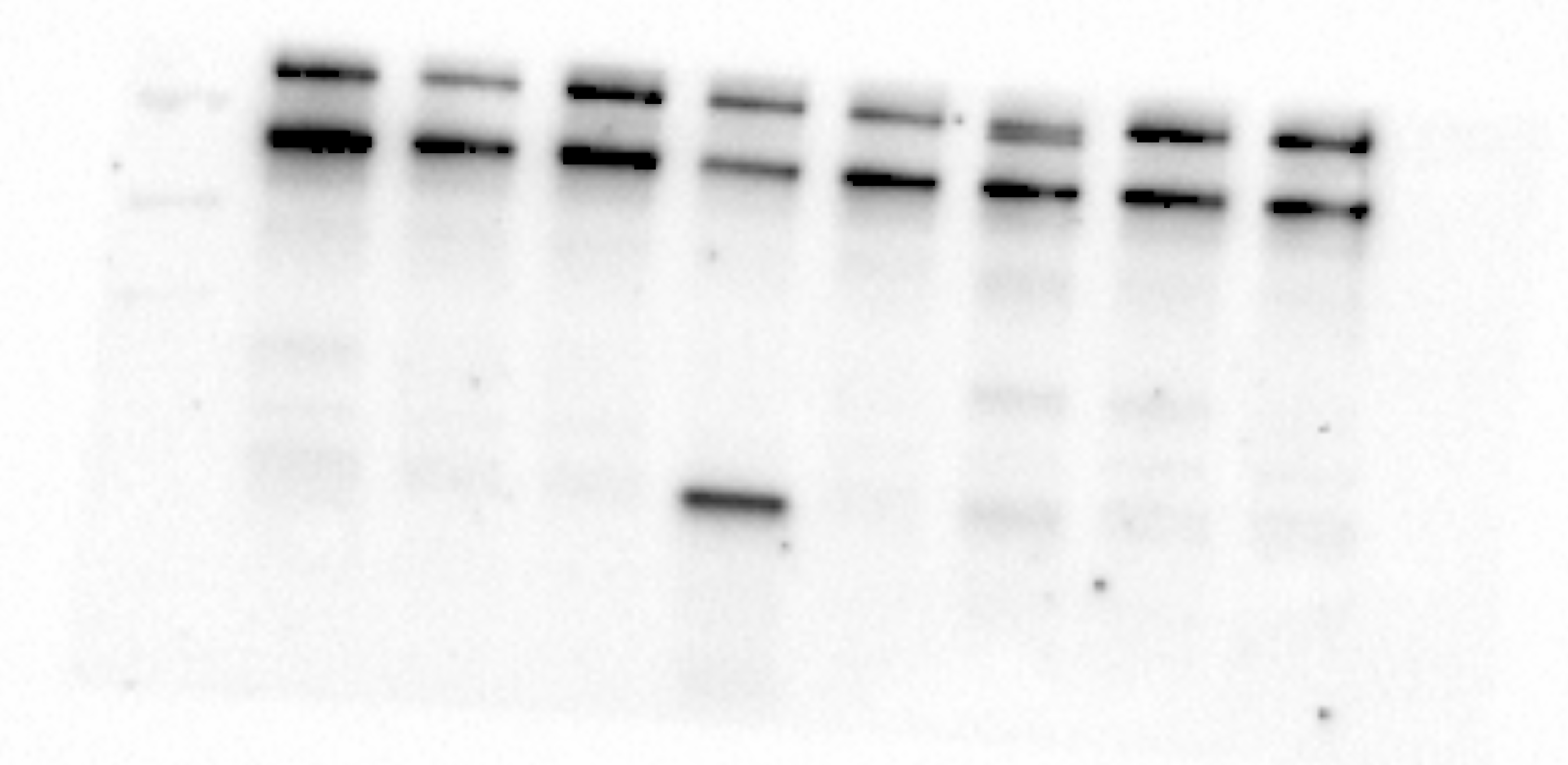

Supplement: Figure 3—source data 1. [file elife-89974-fig3-data1.zip › Figure 3-source data 1/Figure 3A-source data.tif]

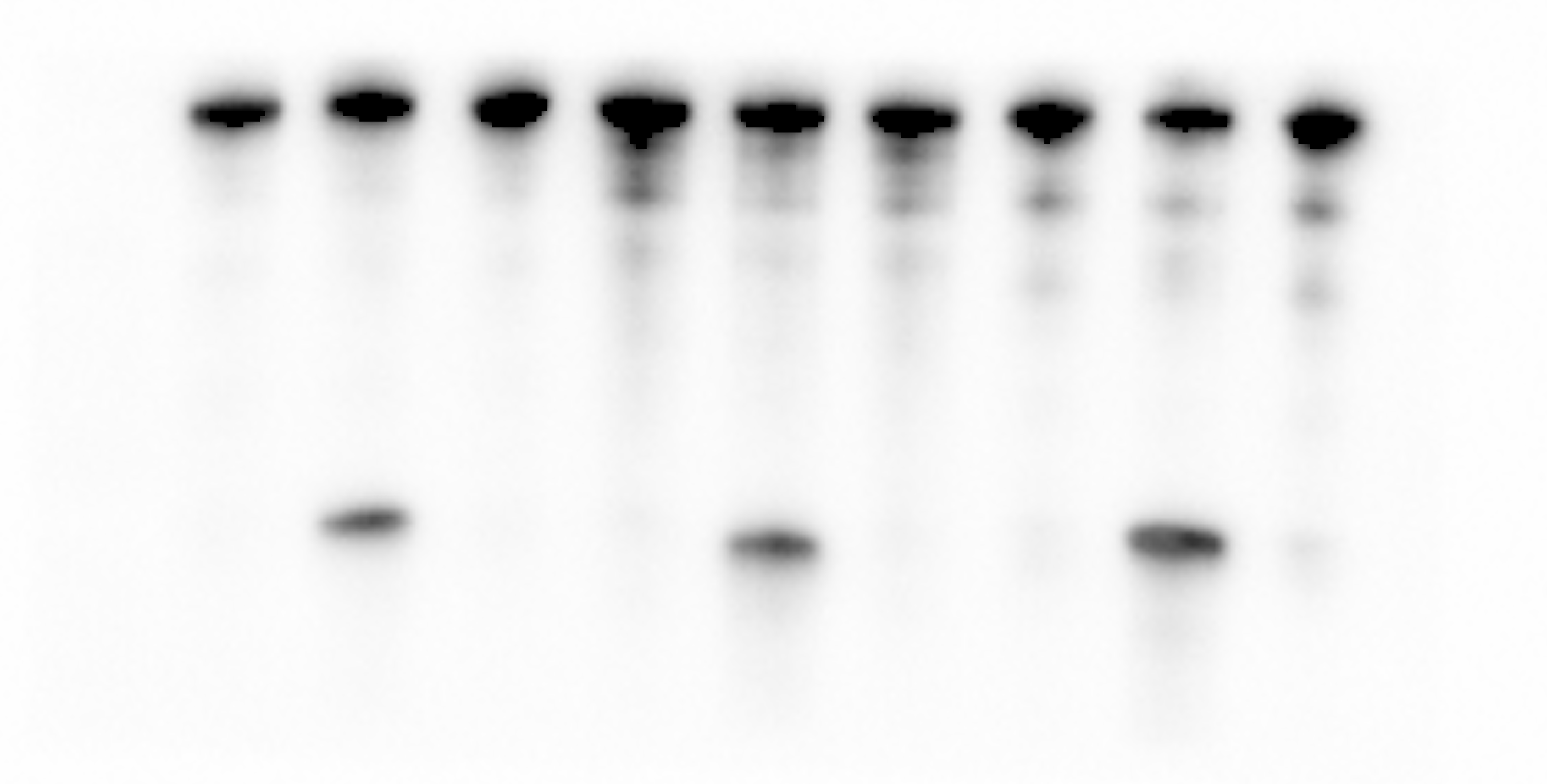

Supplement: Figure 3—source data 1. [file elife-89974-fig3-data1.zip › Figure 3-source data 1/Figure 3D-source data.tif]

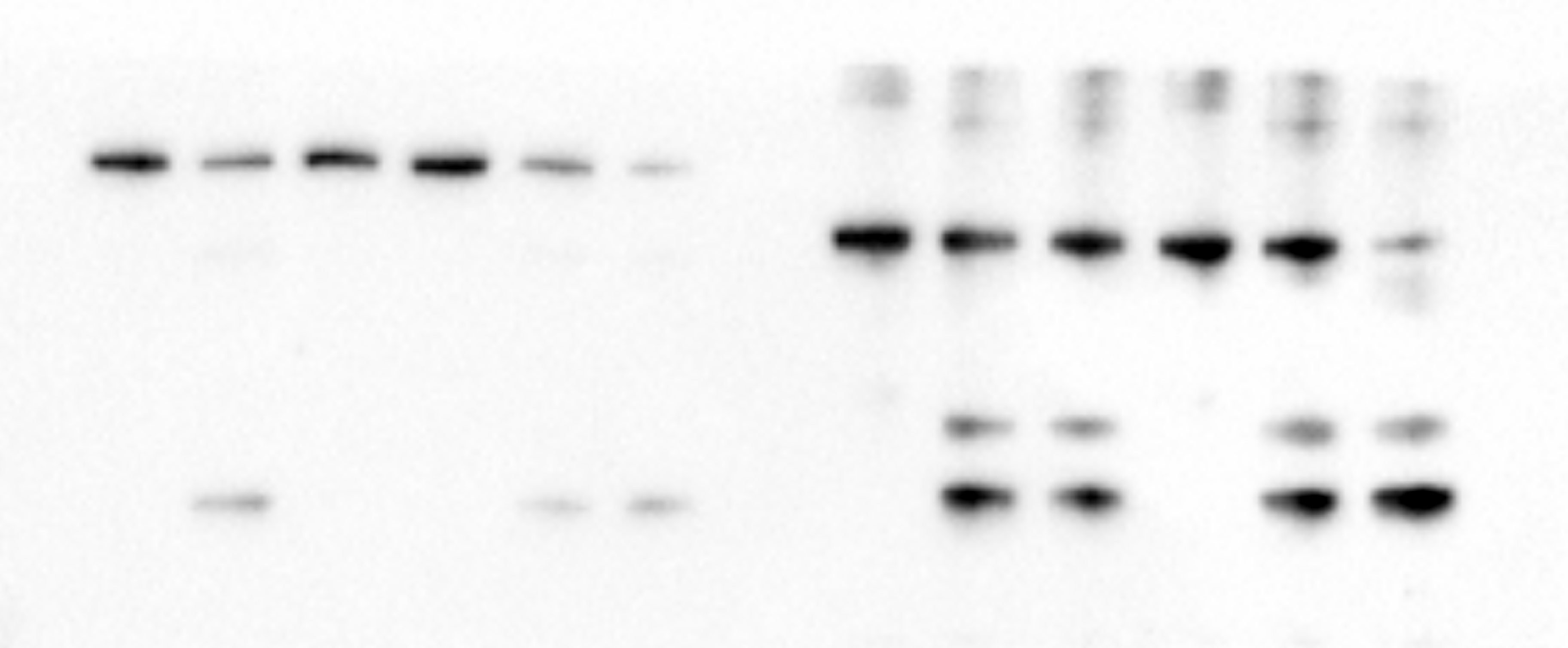

Supplement: Figure 3—source data 1. [file elife-89974-fig3-data1.zip › Figure 3-source data 1/Figure 3F-source data.tif]

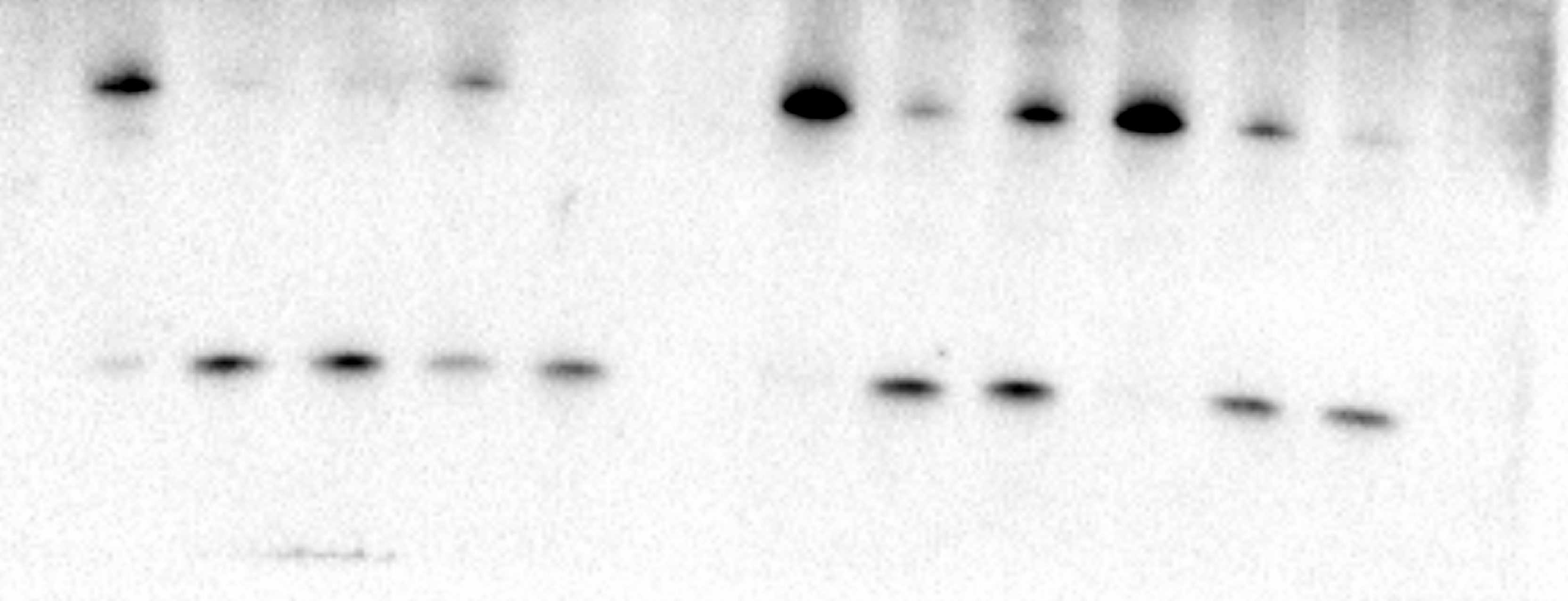

Supplement: Figure 3—source data 1. [file elife-89974-fig3-data1.zip › Figure 3-source data 1/Figure 3G-source data.tif]

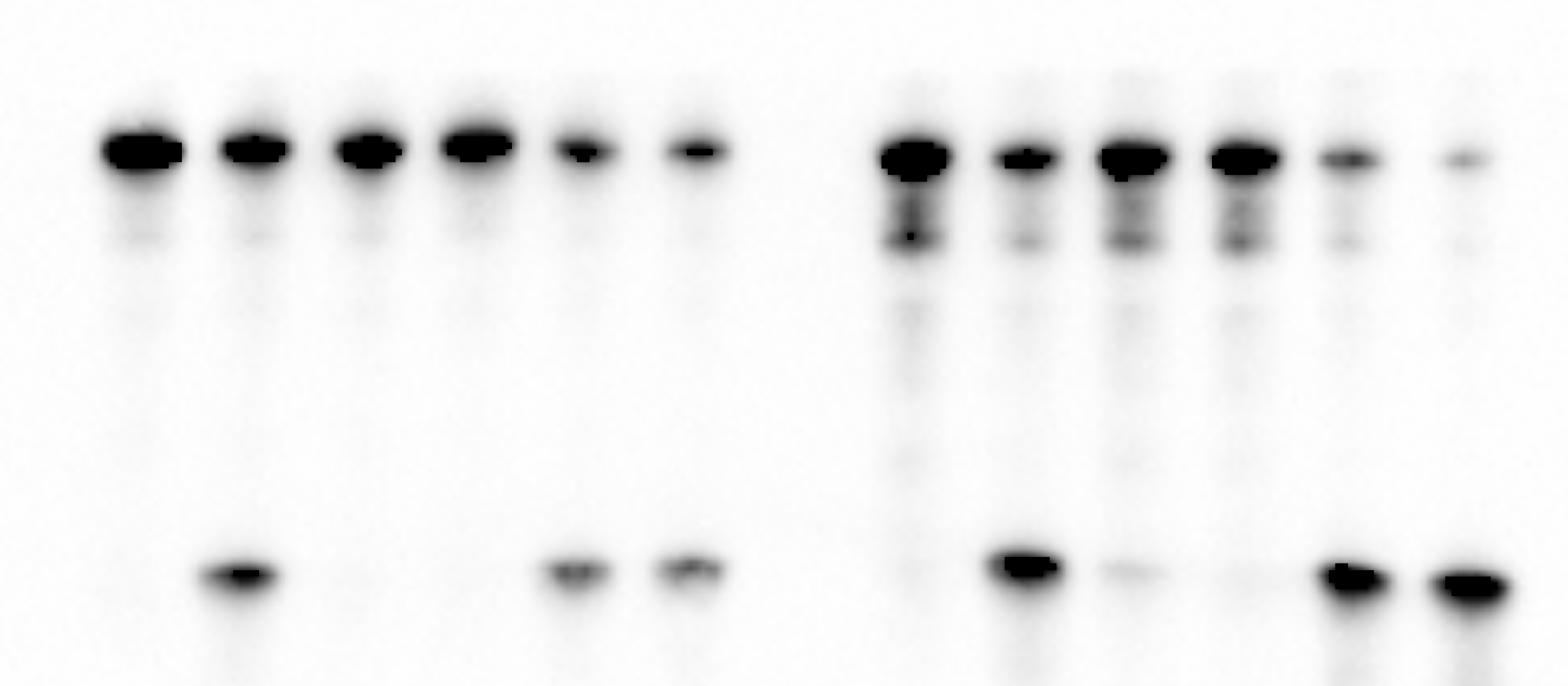

Supplement: Figure 3—source data 1. [file elife-89974-fig3-data1.zip › Figure 3-source data 1/Figure 3H-source data.tif]

**Figure 3A**

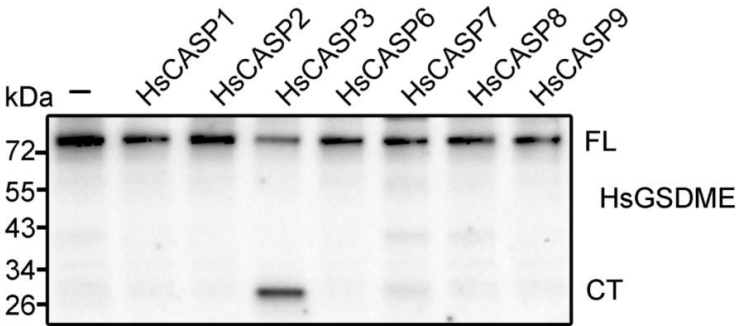

**anti-HsGSDME-CT**

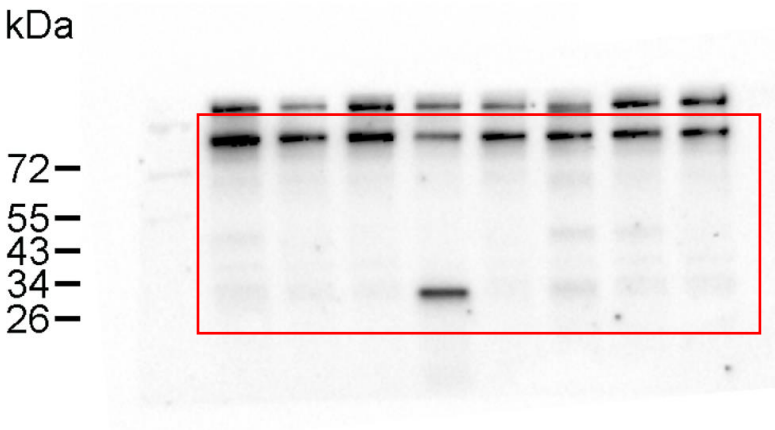

Supplement: Figure 3—source data 2. [file elife-89974-fig3-data2.zip › Figure 3-source data 2/Figure 3A-source data.pdf]

**Figure 3D**

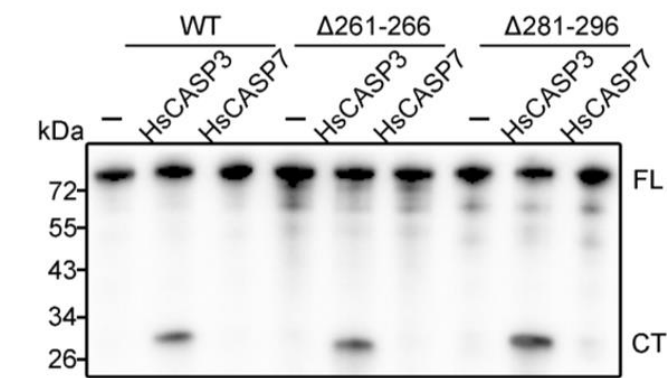

**anti-HsGSDME-CT**

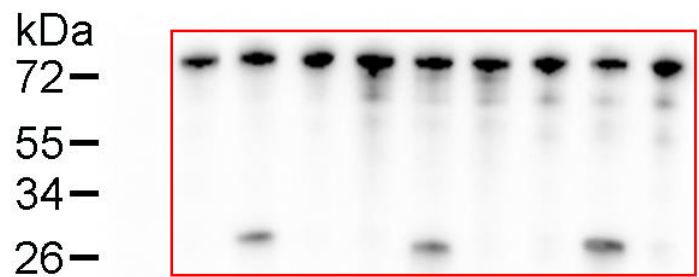

Supplement: Figure 3—source data 2. [file elife-89974-fig3-data2.zip › Figure 3-source data 2/Figure 3D-source data.pdf]

**Figure 3F**

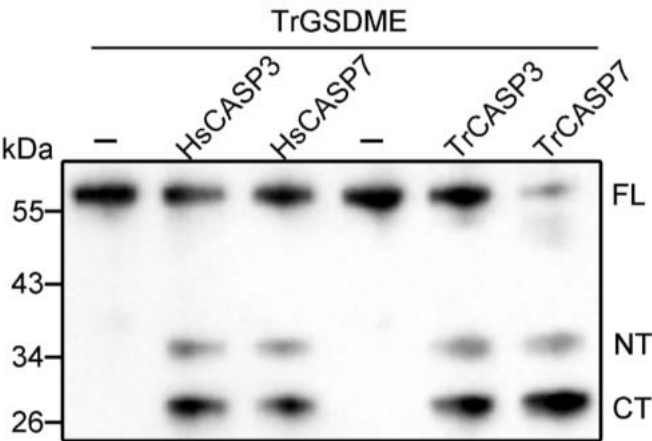

**anti-TrGSDME**

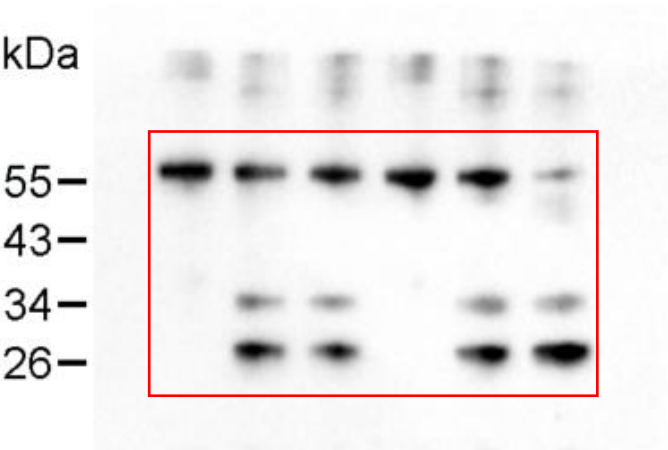

Supplement: Figure 3—source data 2. [file elife-89974-fig3-data2.zip › Figure 3-source data 2/Figure 3F-source data.pdf]

**Figure 3G**

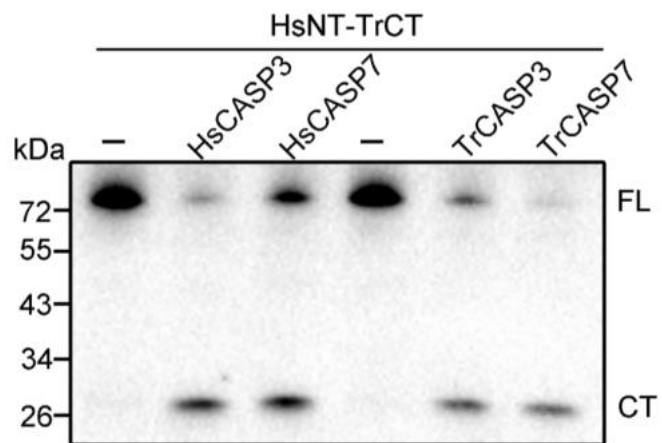

**anti-TrGSDME**

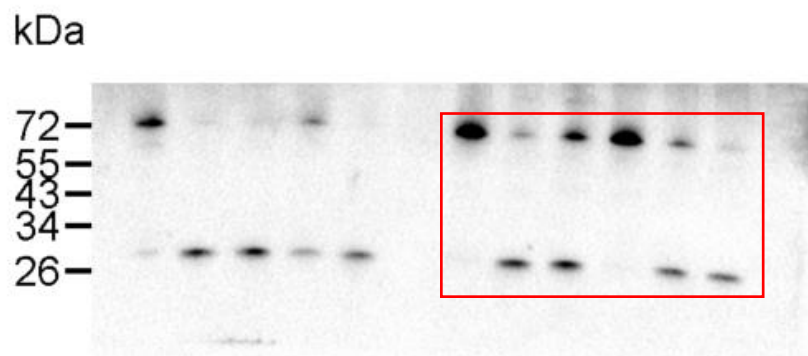

Supplement: Figure 3—source data 2. [file elife-89974-fig3-data2.zip › Figure 3-source data 2/Figure 3G-source data.pdf]

**Figure 3H**

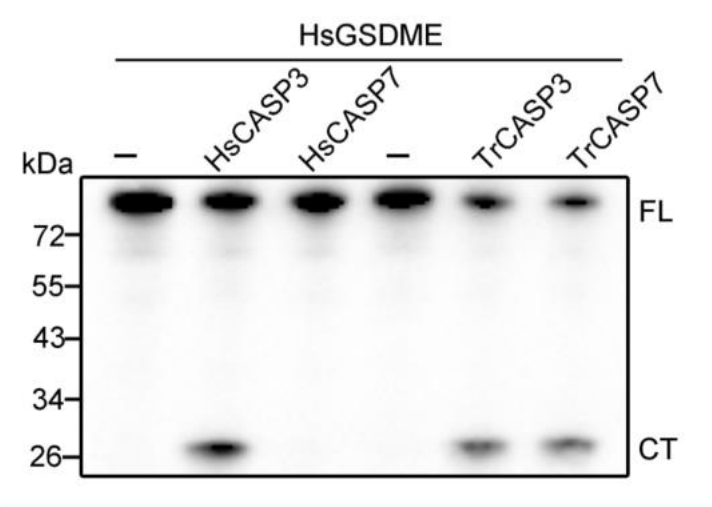

**anti-HsGSDME-CT**

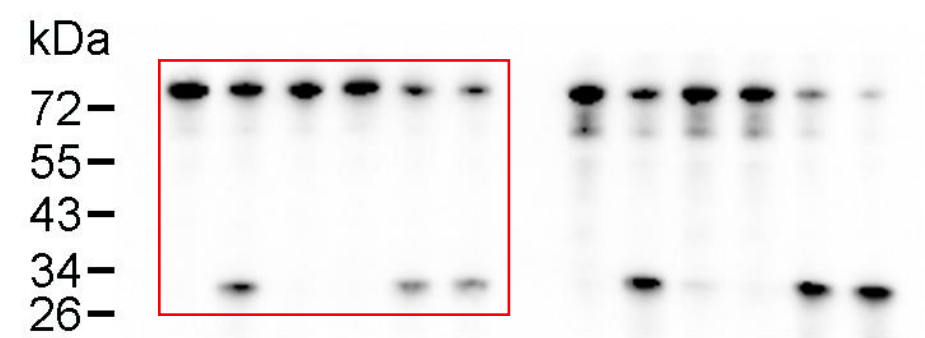

Supplement: Figure 3—source data 2. [file elife-89974-fig3-data2.zip › Figure 3-source data 2/Figure 3H-source data.pdf]

**Figure 3I**

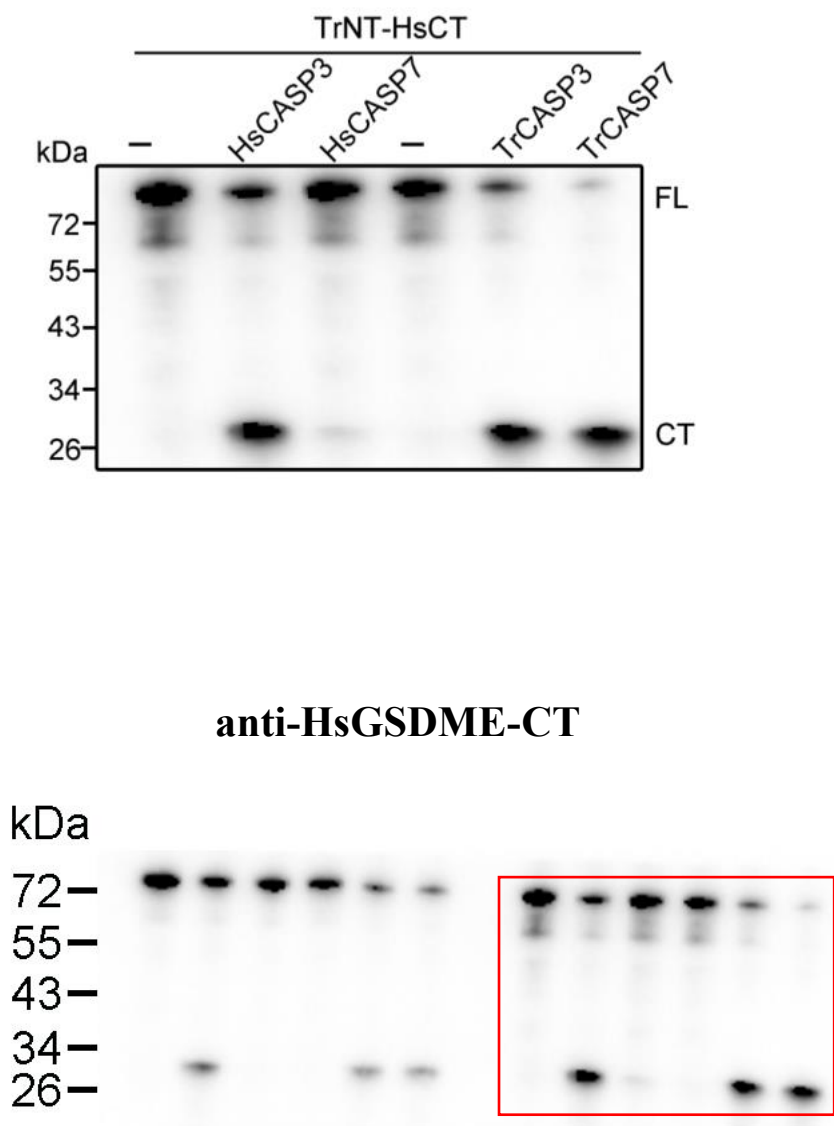

Supplement: Figure 3—source data 2. [file elife-89974-fig3-data2.zip › Figure 3-source data 2/Figure 3I-source data.pdf]

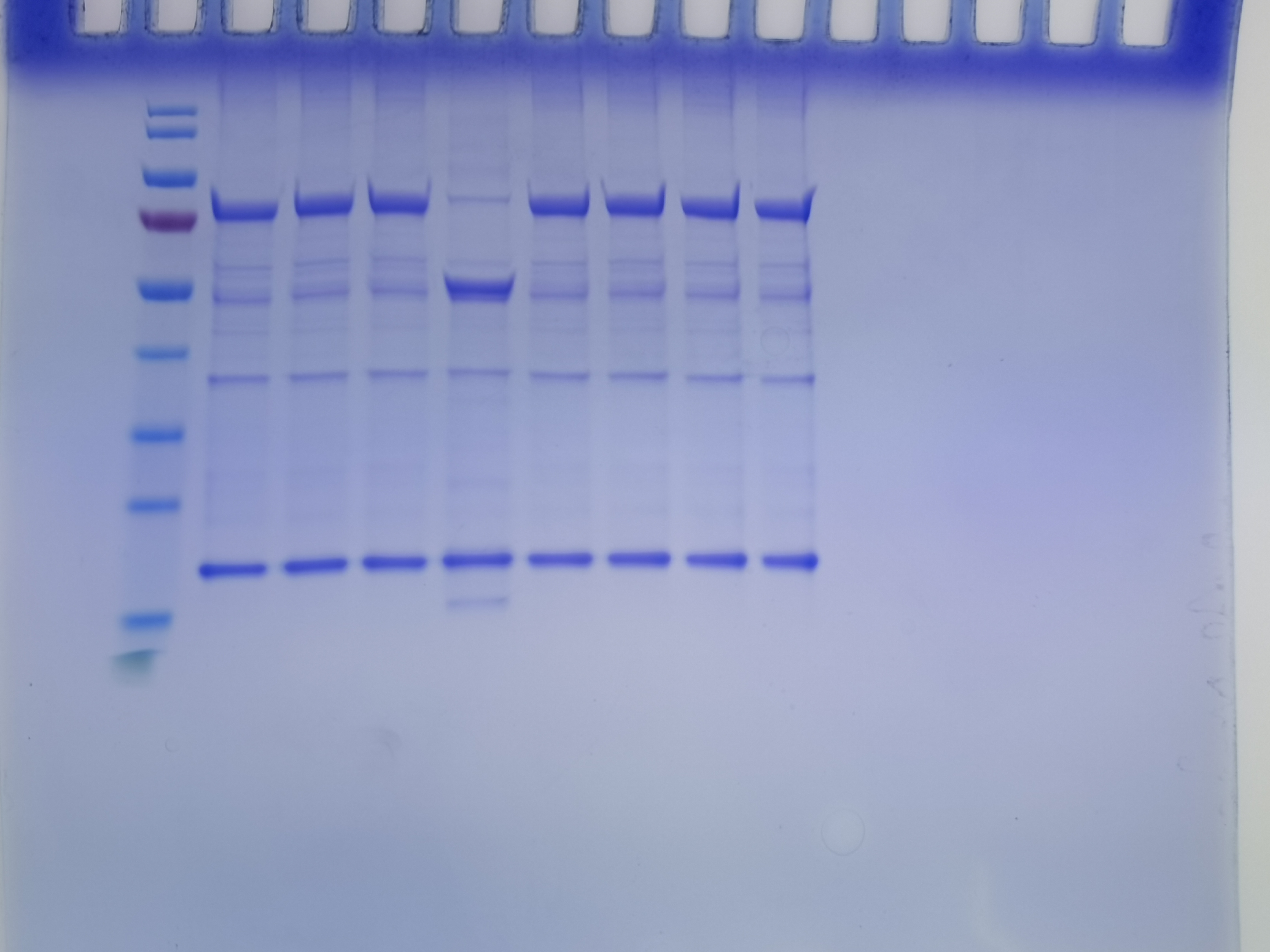

Supplement: Figure 3—figure supplement 1—source data 1. [file elife-89974-fig3-figsupp1-data1.zip › Figure 3-figure supplement 1-source data 1/Figure 3-figure supplement 1-source data.tif]

Figure 3-figure supplement 1

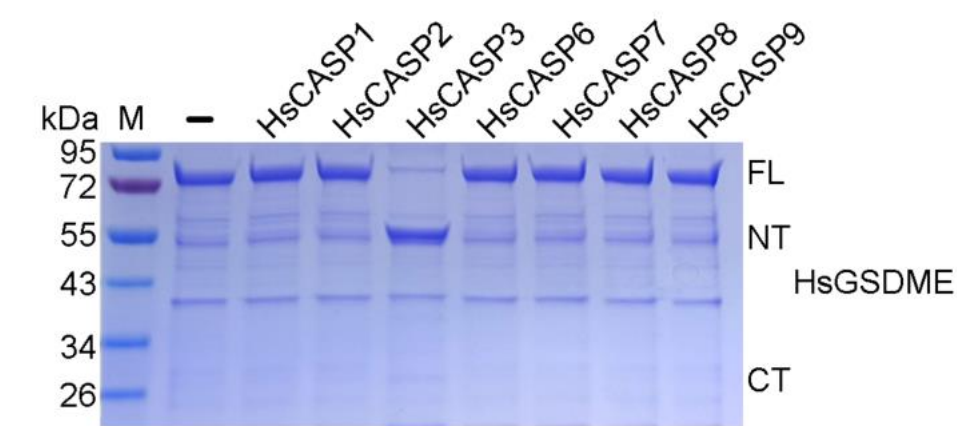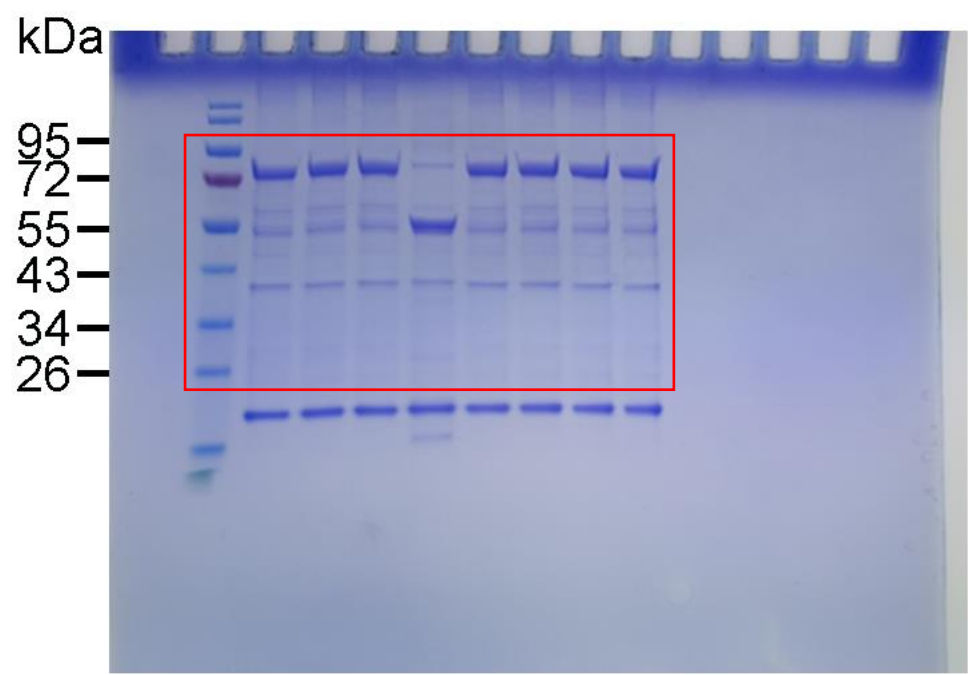

Supplement: Figure 3—figure supplement 1—source data 2. [file elife-89974-fig3-figsupp1-data2.zip › Figure 3-figure supplement 1-source data 2/Figure 3-figure supplement 1-source data.pdf]

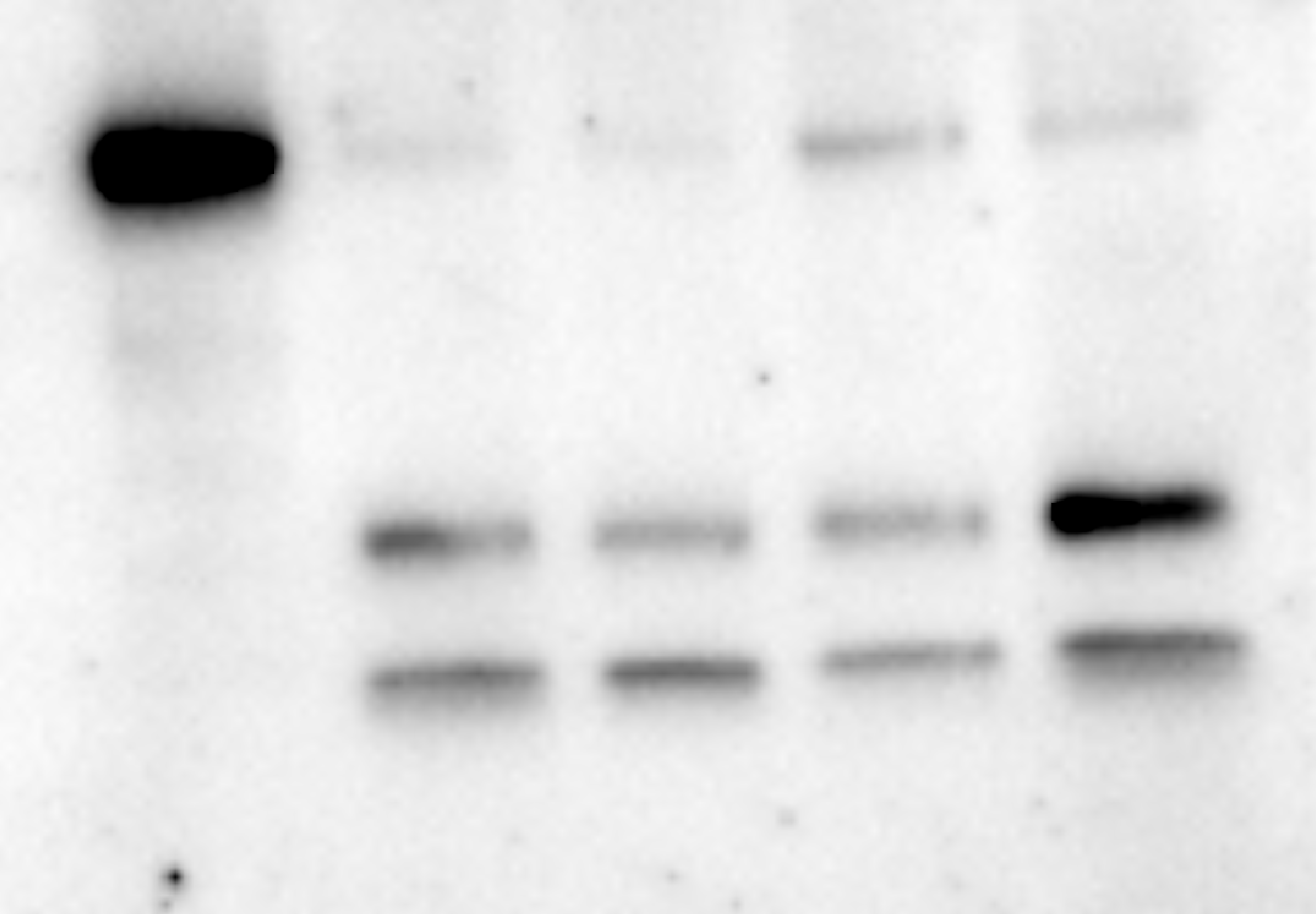

Supplement: Figure 4—source data 1. [file elife-89974-fig4-data1.zip › Figure 4-source data 1/Figure 4D-source data.tif]

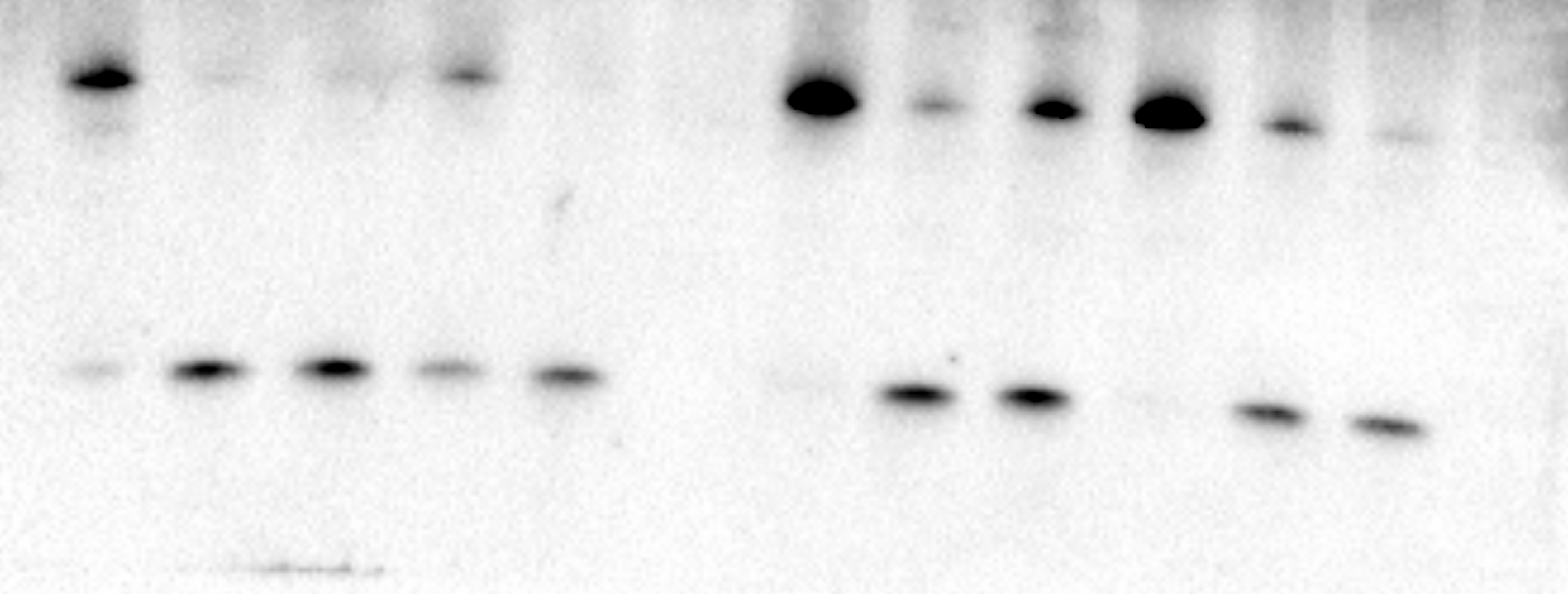

Supplement: Figure 4—source data 1. [file elife-89974-fig4-data1.zip › Figure 4-source data 1/Figure 4E-source data.tif]

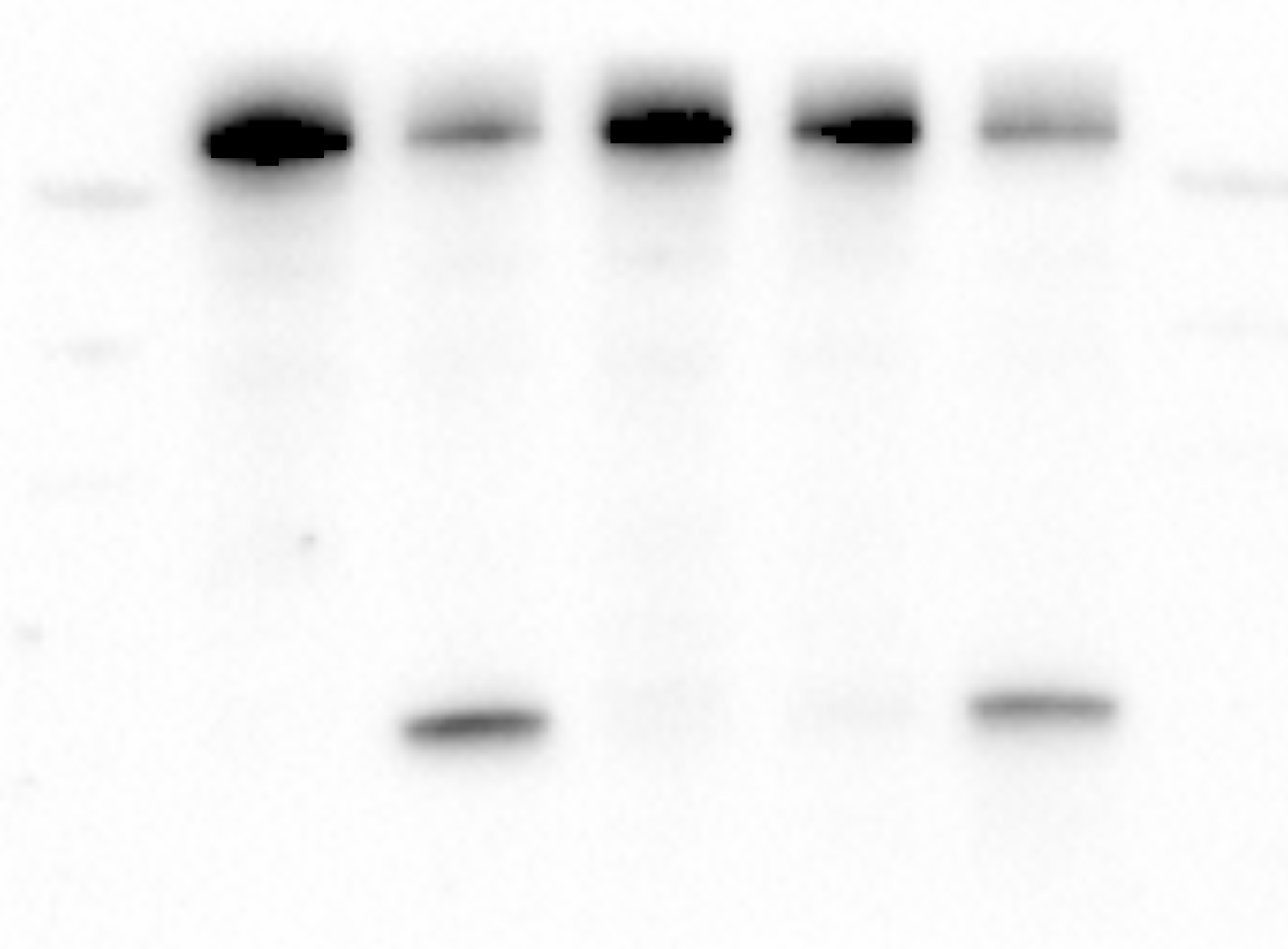

Supplement: Figure 4—source data 1. [file elife-89974-fig4-data1.zip › Figure 4-source data 1/Figure 4F-source data.tif]

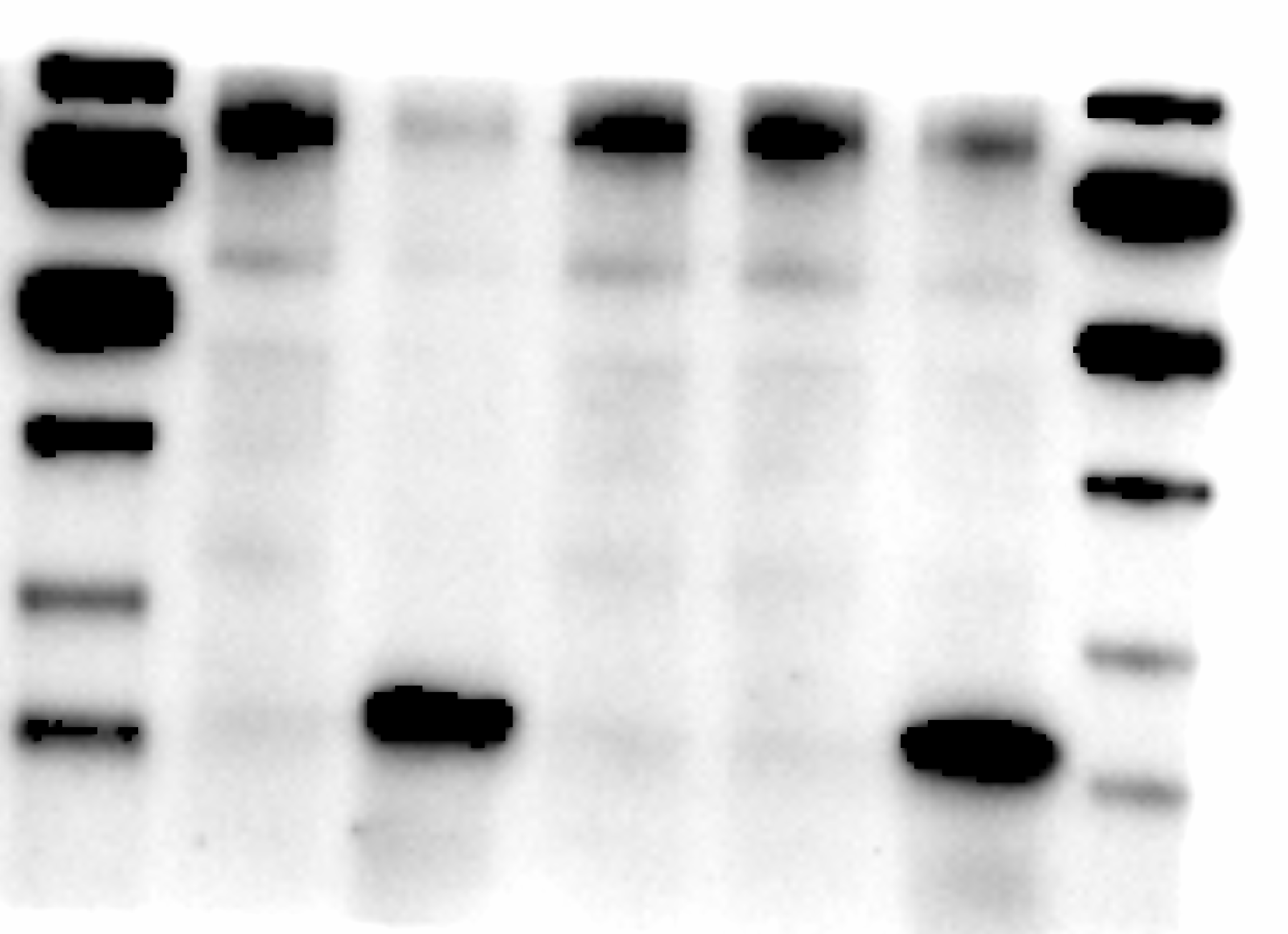

Supplement: Figure 4—source data 1. [file elife-89974-fig4-data1.zip › Figure 4-source data 1/Figure 4G-source data.tif]

**Figure 4D**

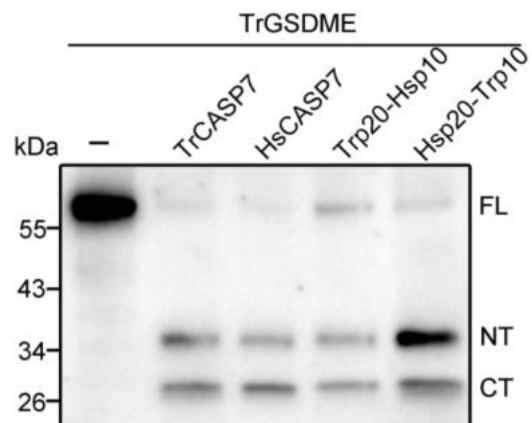

**anti-TrGSDME**

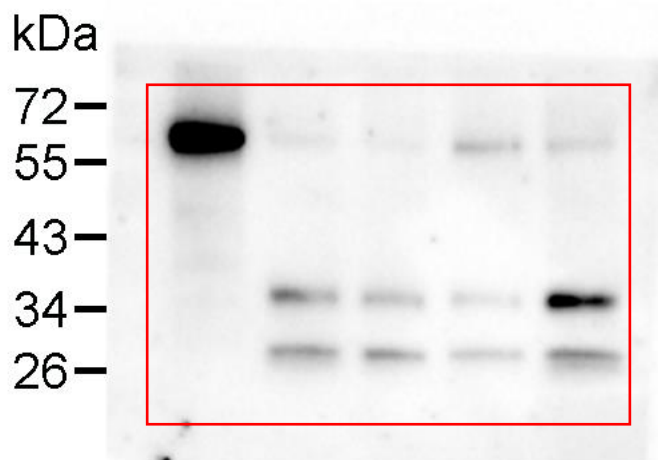

Supplement: Figure 4—source data 2. [file elife-89974-fig4-data2.zip › Figure 4-source data 2/Figure 4D-source data.pdf]

**Figure 4E**

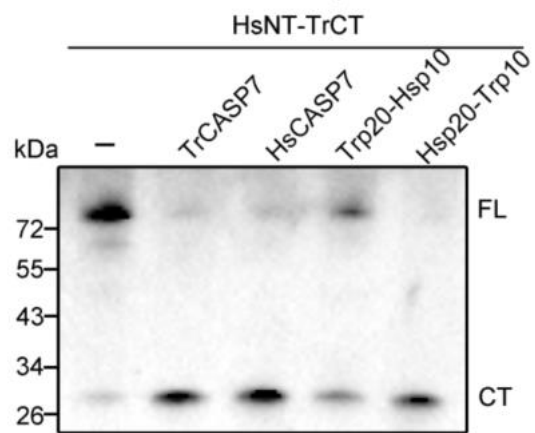

**anti-TrGSDME**

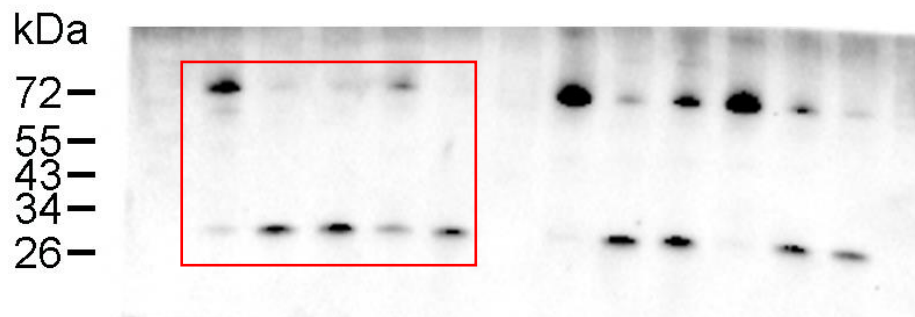

Supplement: Figure 4—source data 2. [file elife-89974-fig4-data2.zip › Figure 4-source data 2/Figure 4E-source data.pdf]

**Figure 4F**

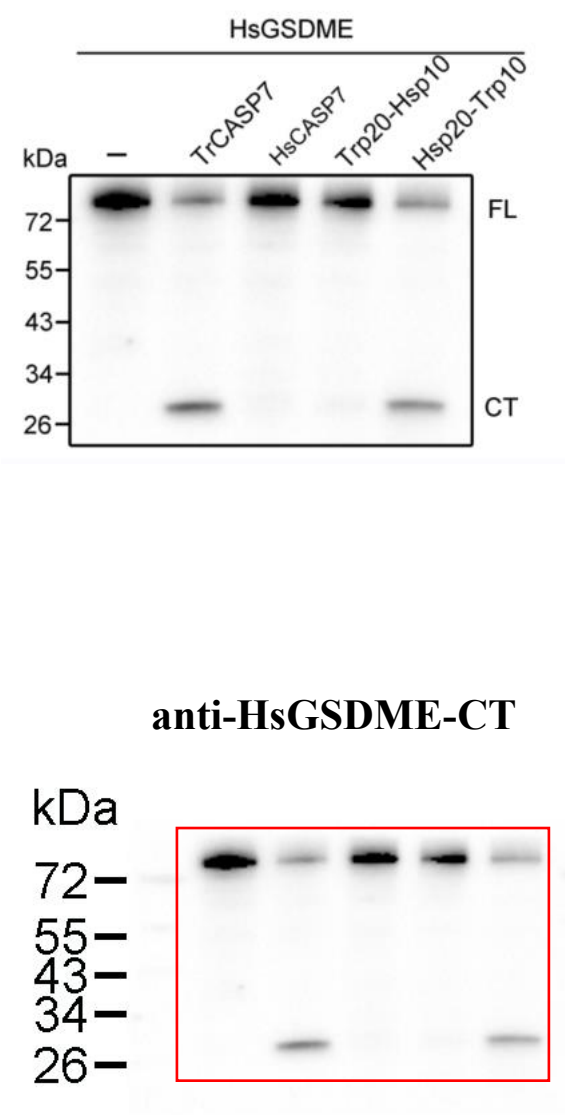

Supplement: Figure 4—source data 2. [file elife-89974-fig4-data2.zip › Figure 4-source data 2/Figure 4F-source data.pdf]

**Figure 4G**

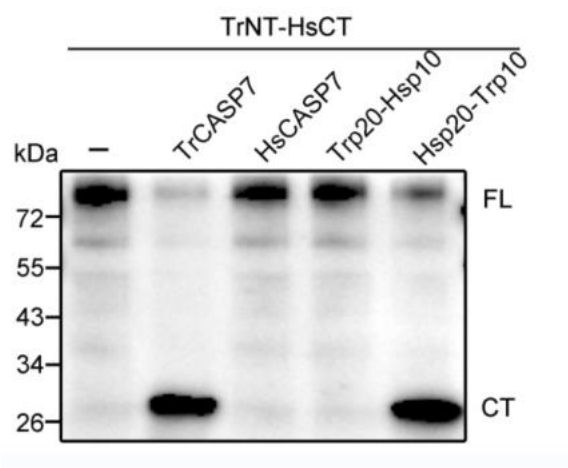

**anti-HsGSDME-CT**

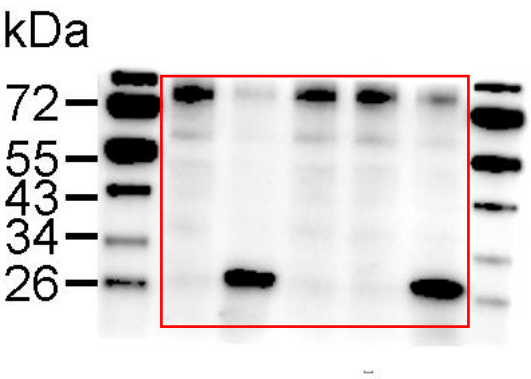

Supplement: Figure 4—source data 2. [file elife-89974-fig4-data2.zip › Figure 4-source data 2/Figure 4G-source data.pdf]

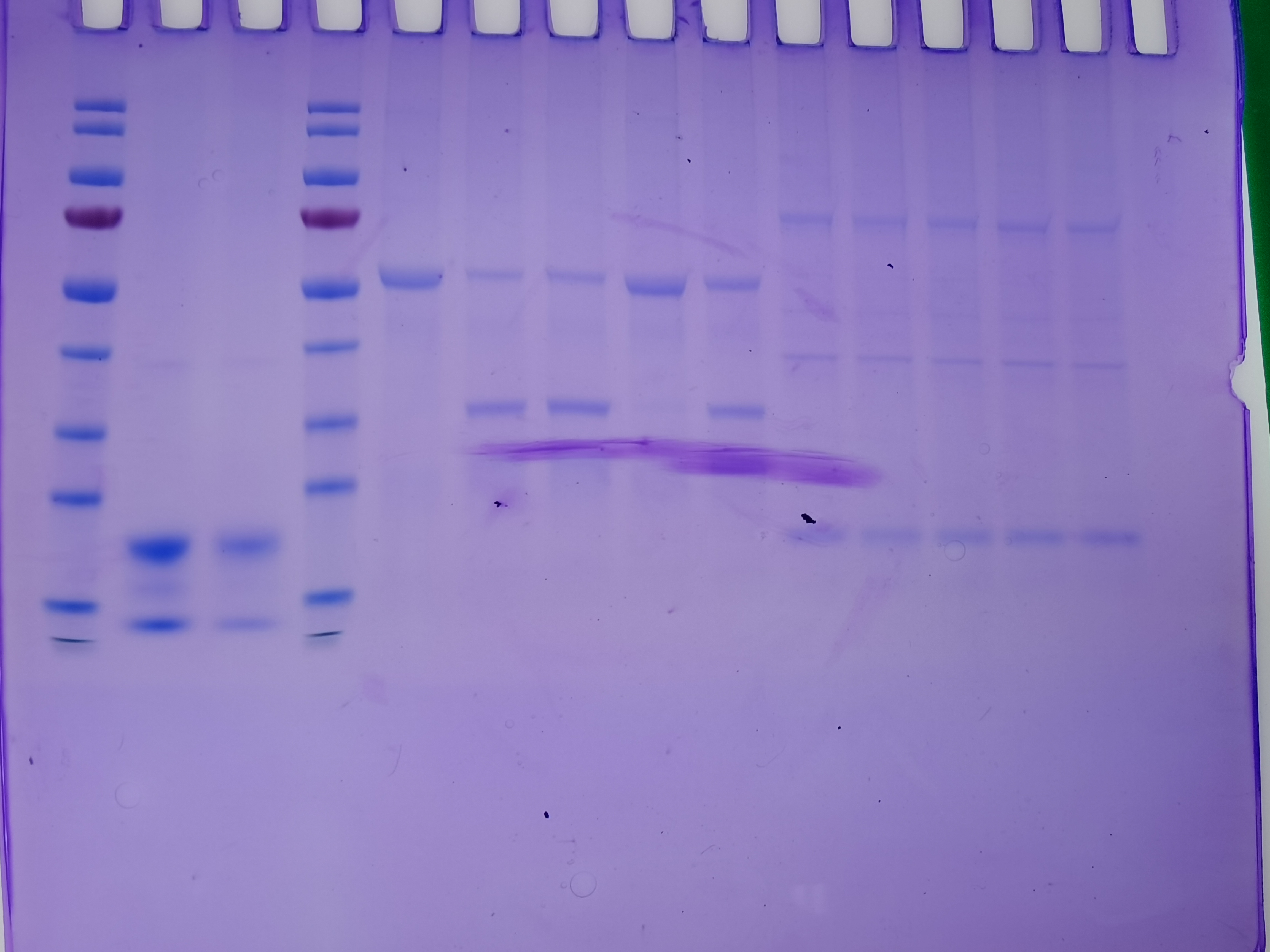

Supplement: Figure 4—figure supplement 1—source data 1. [file elife-89974-fig4-figsupp1-data1.zip › Figure 4-figure supplement 1-source data 1/Figure 4-figure supplement 1A-source data.tif]

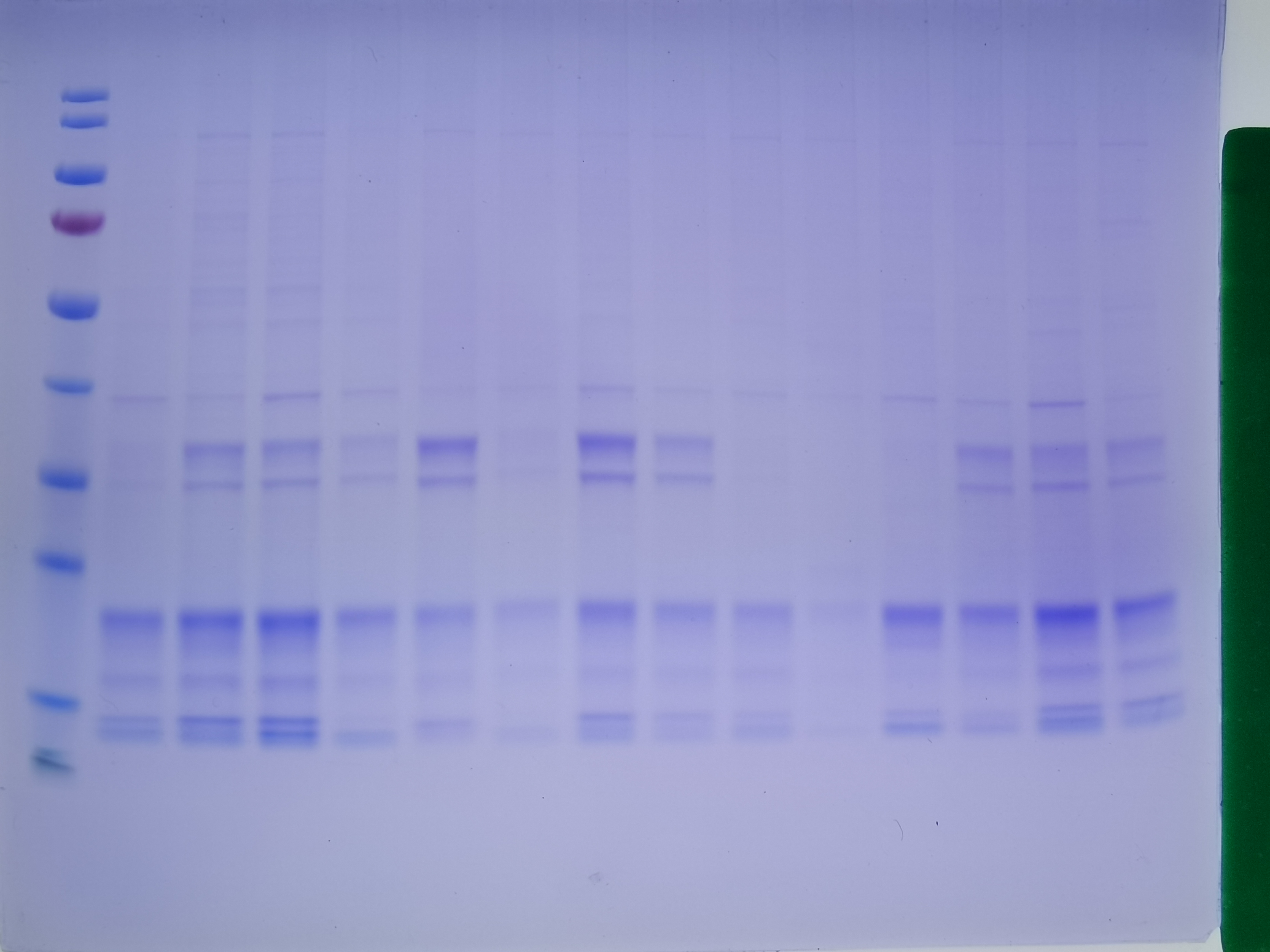

Supplement: Figure 4—figure supplement 1—source data 1. [file elife-89974-fig4-figsupp1-data1.zip › Figure 4-figure supplement 1-source data 1/Figure 4-figure supplement 1B-SDS PAGE-source data.tif]

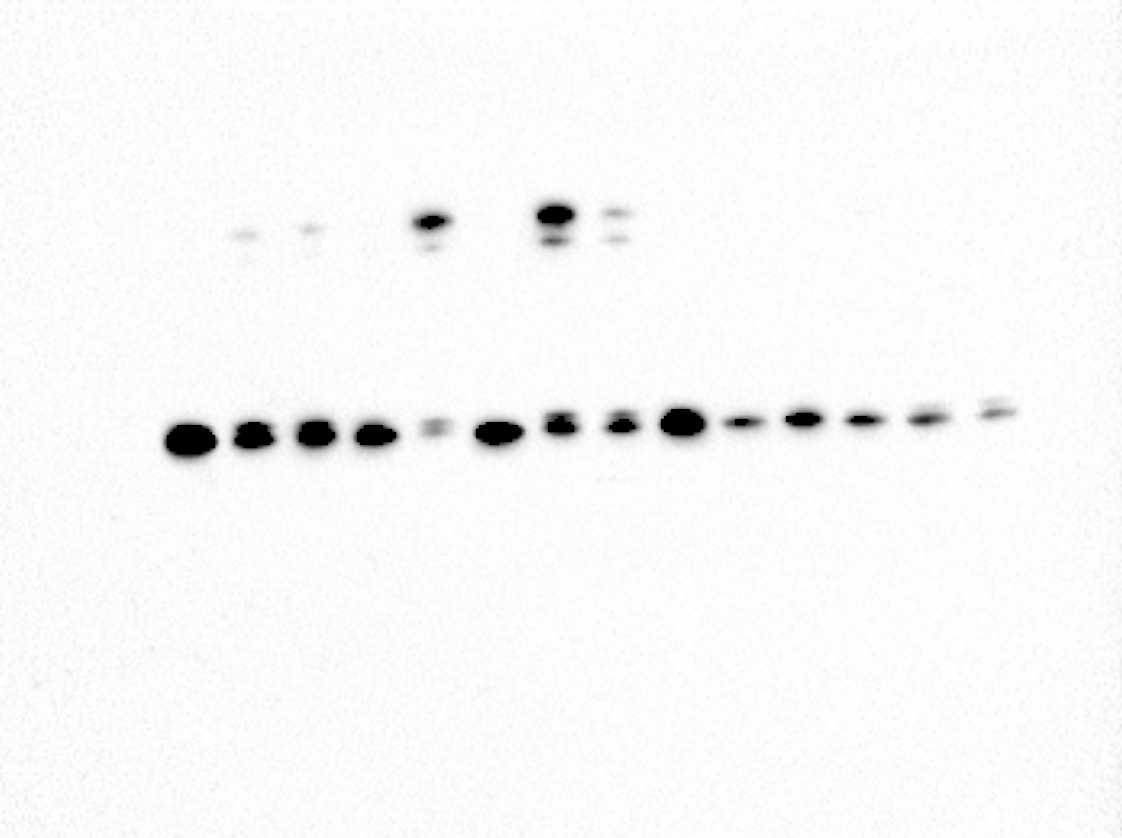

Supplement: Figure 4—figure supplement 1—source data 1. [file elife-89974-fig4-figsupp1-data1.zip › Figure 4-figure supplement 1-source data 1/Figure 4-figure supplement 1B-WB-source data.tif]

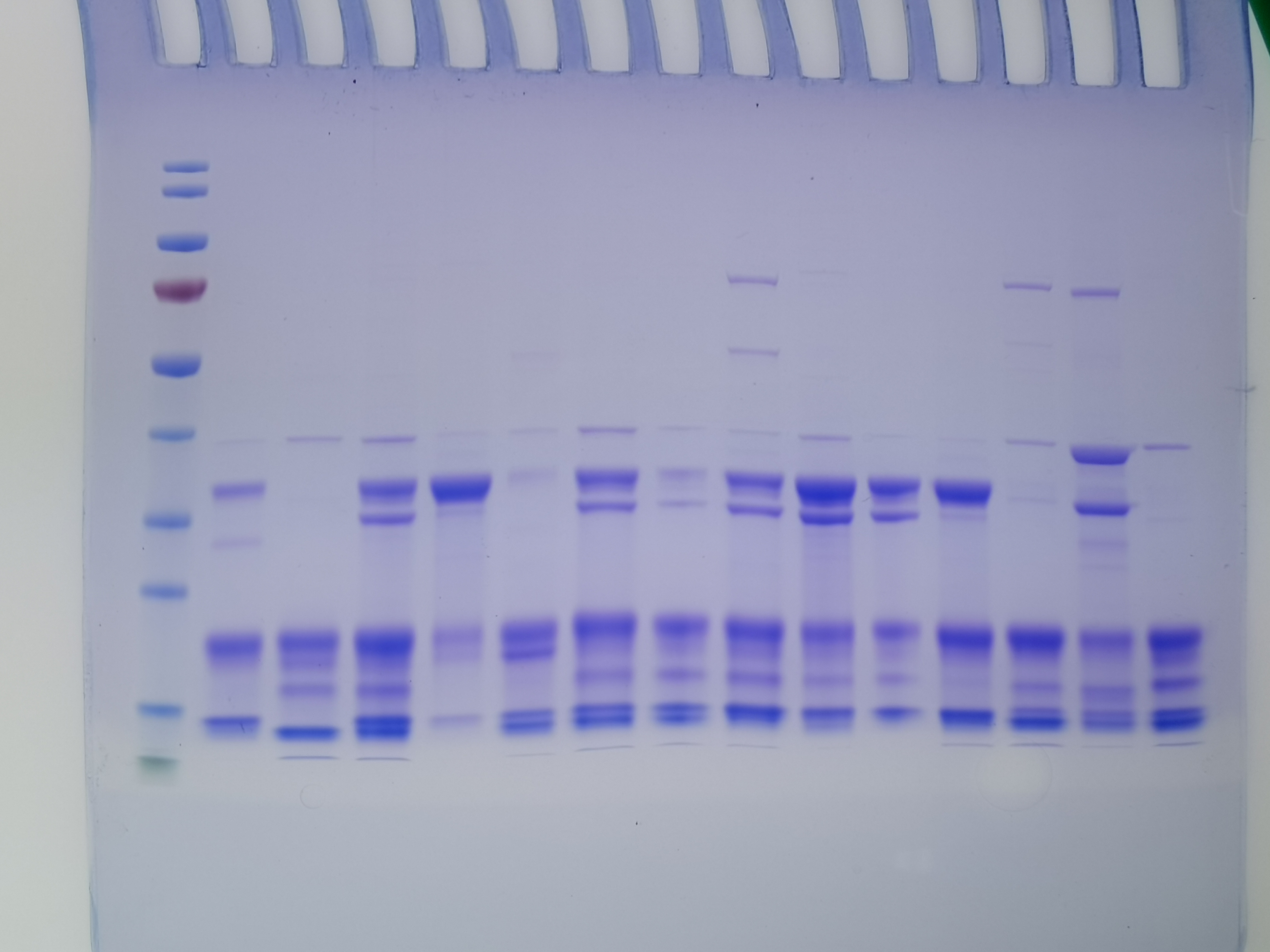

Supplement: Figure 4—figure supplement 1—source data 1. [file elife-89974-fig4-figsupp1-data1.zip › Figure 4-figure supplement 1-source data 1/Figure 4-figure supplement 1C-SDS PAGE-source data.tif]

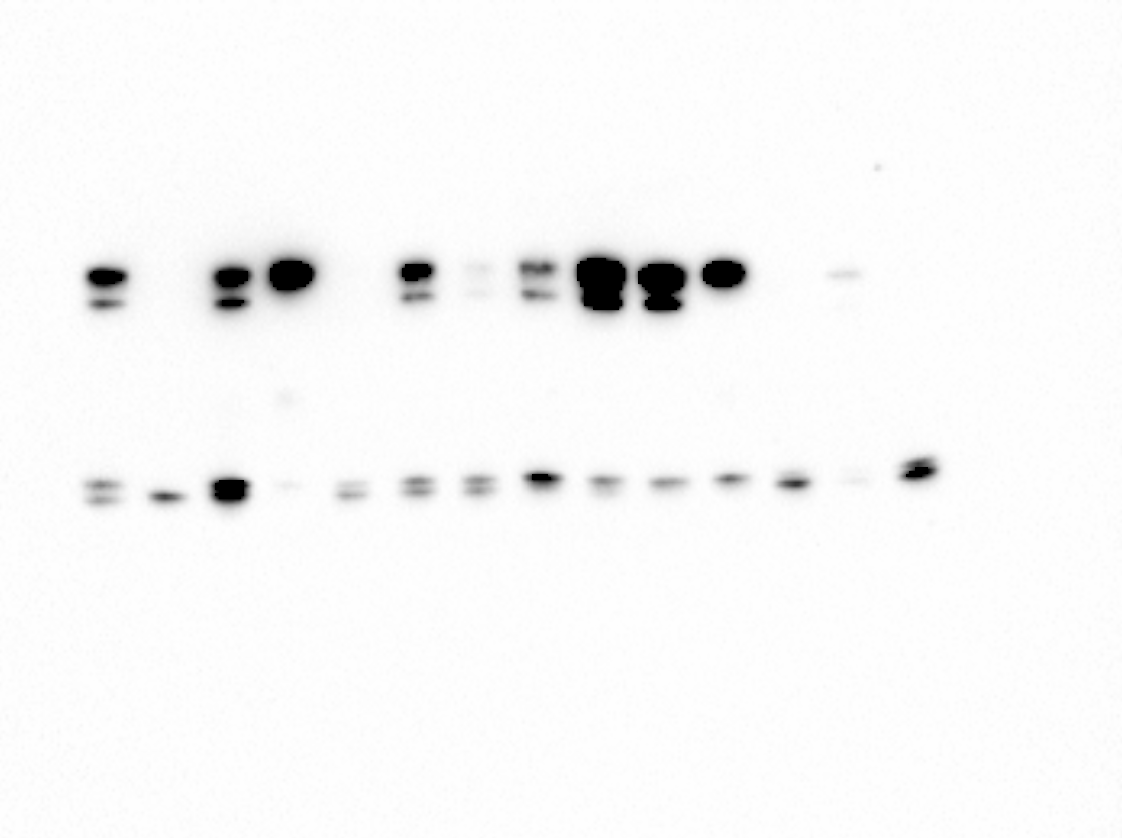

Supplement: Figure 4—figure supplement 1—source data 1. [file elife-89974-fig4-figsupp1-data1.zip › Figure 4-figure supplement 1-source data 1/Figure 4-figure supplement 1C-WB-source data.tif]

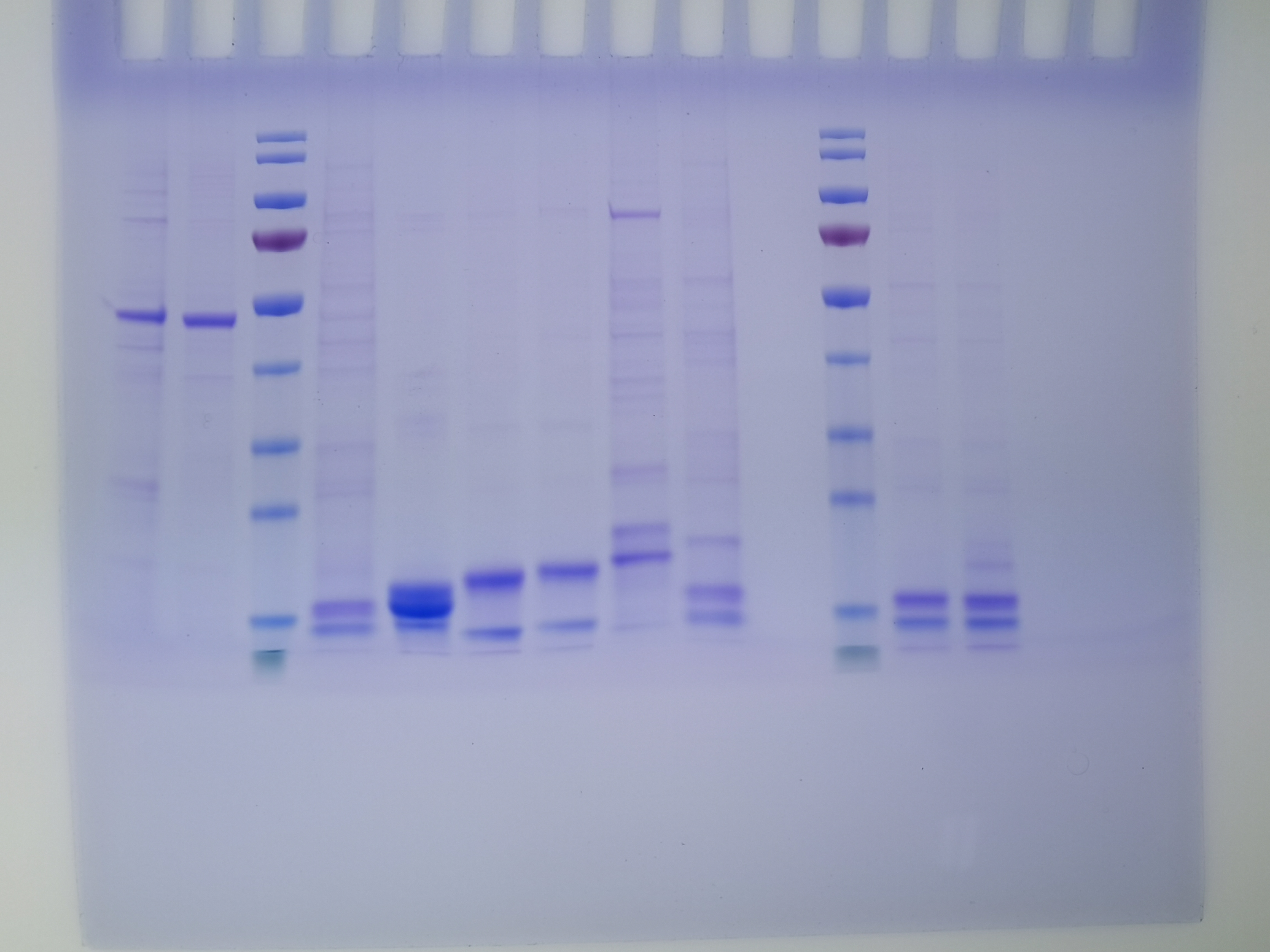

Supplement: Figure 4—figure supplement 1—source data 1. [file elife-89974-fig4-figsupp1-data1.zip › Figure 4-figure supplement 1-source data 1/Figure 4-figure supplement 1D-source data.tif]

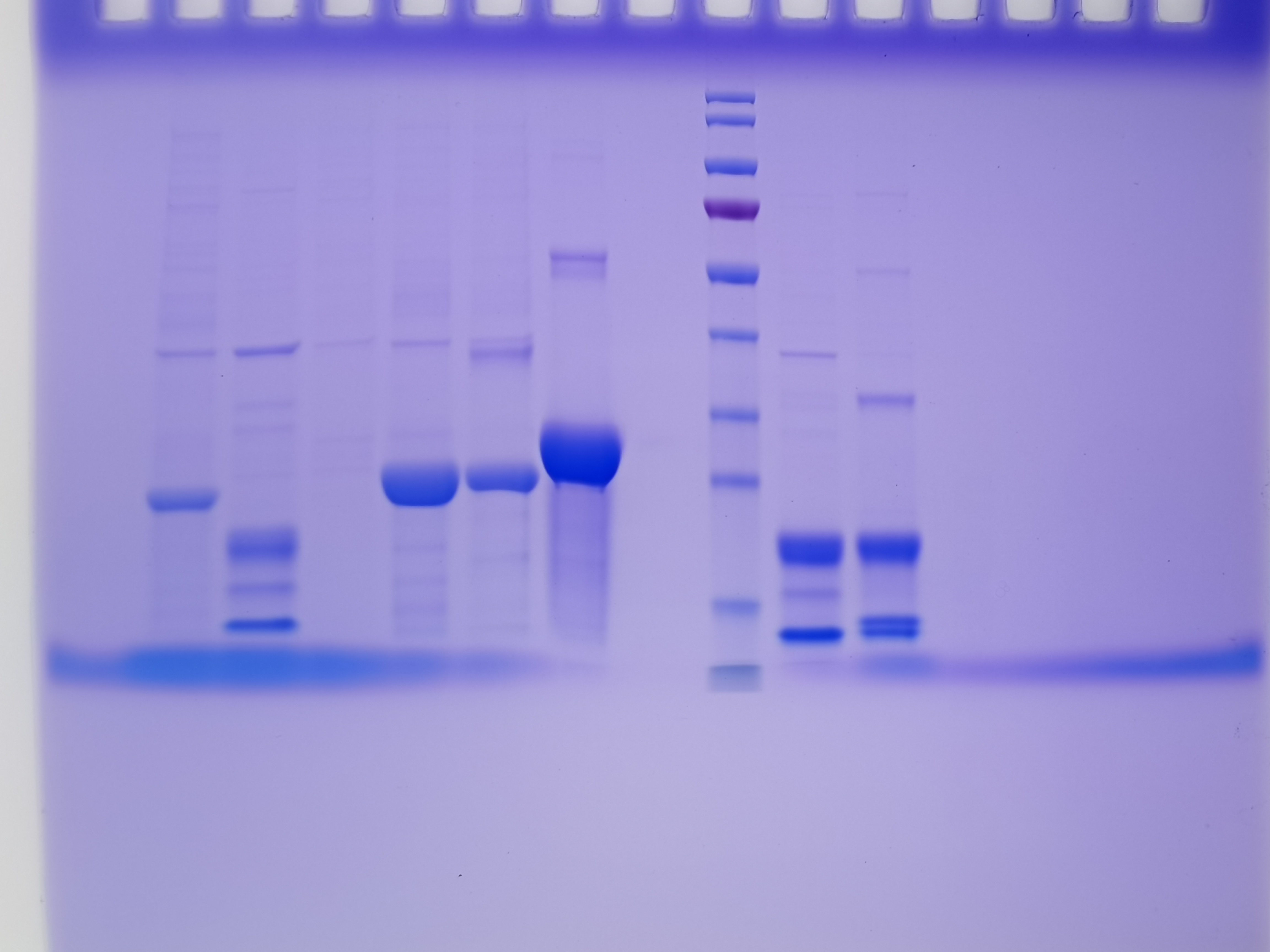

Supplement: Figure 4—figure supplement 1—source data 1. [file elife-89974-fig4-figsupp1-data1.zip › Figure 4-figure supplement 1-source data 1/Figure 4-figure supplement 1E-source data.tif]

**Figure 4-figure supplement 1A**

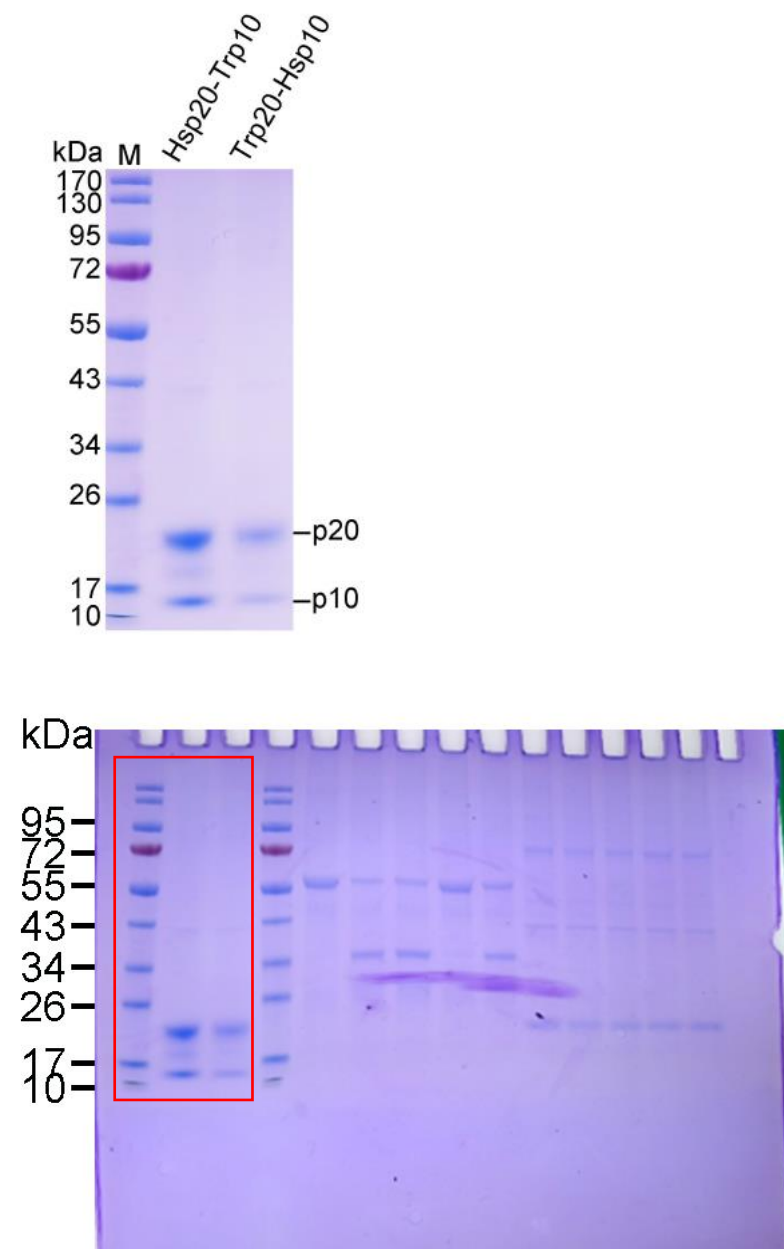

Supplement: Figure 4—figure supplement 1—source data 2. [file elife-89974-fig4-figsupp1-data2.zip › Figure 4-figure supplement 1-source data 2/Figure 4-figure supplement 1A-source data.pdf]

Figure 4-figure supplement 1B

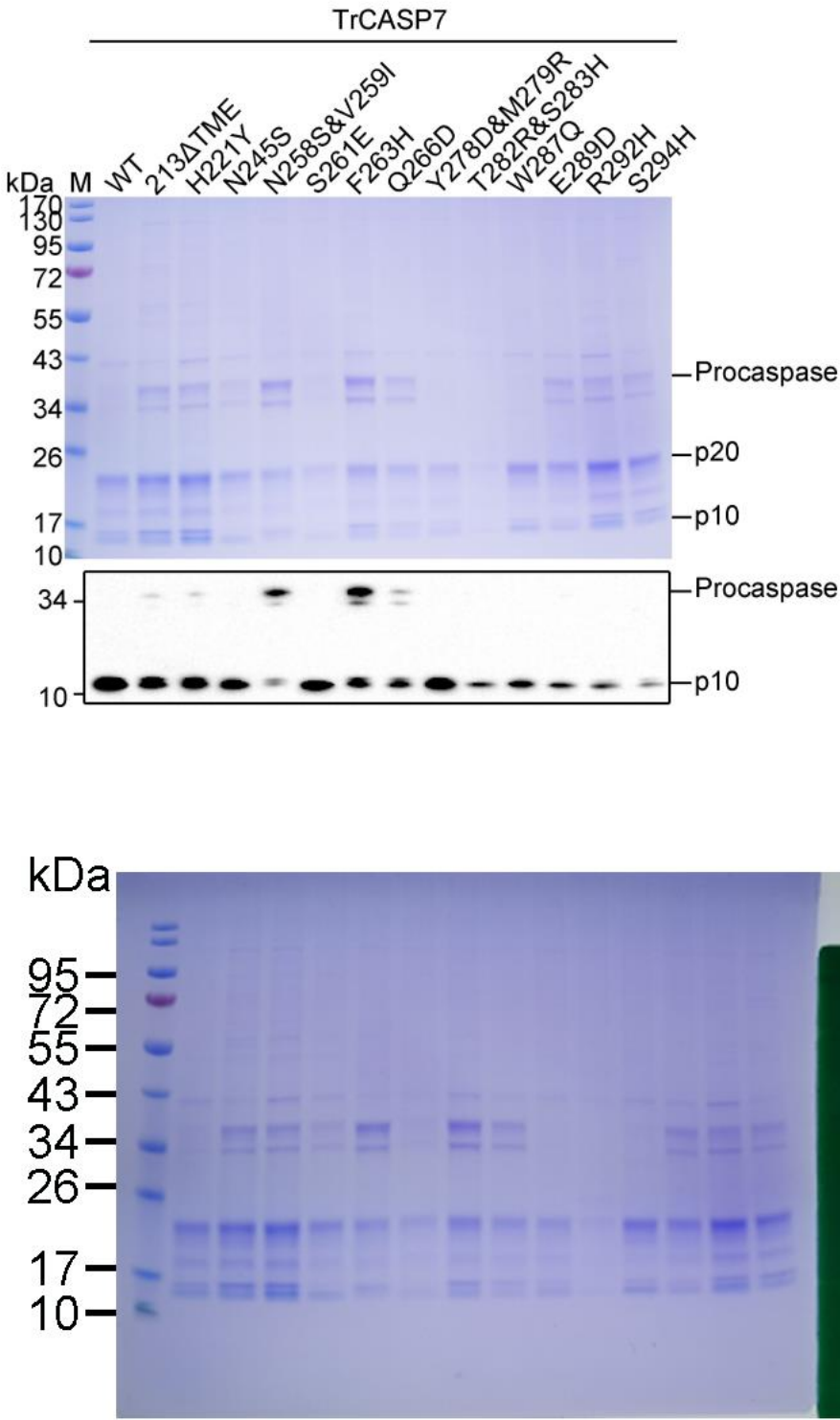

**anti-His**

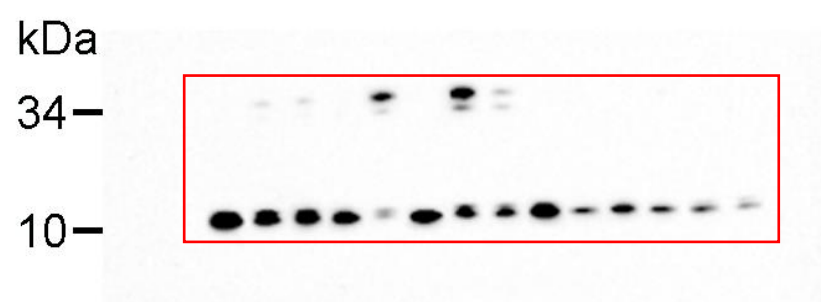

Supplement: Figure 4—figure supplement 1—source data 2. [file elife-89974-fig4-figsupp1-data2.zip › Figure 4-figure supplement 1-source data 2/Figure 4-figure supplement 1B-source data.pdf]

Figure 4-figure supplement 1C

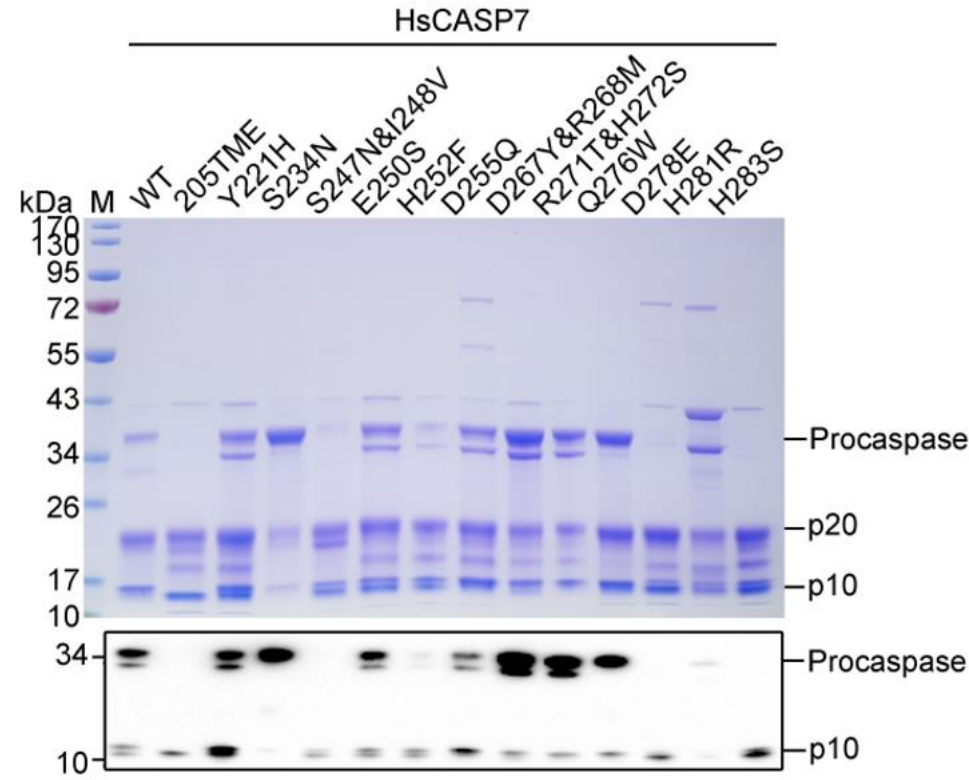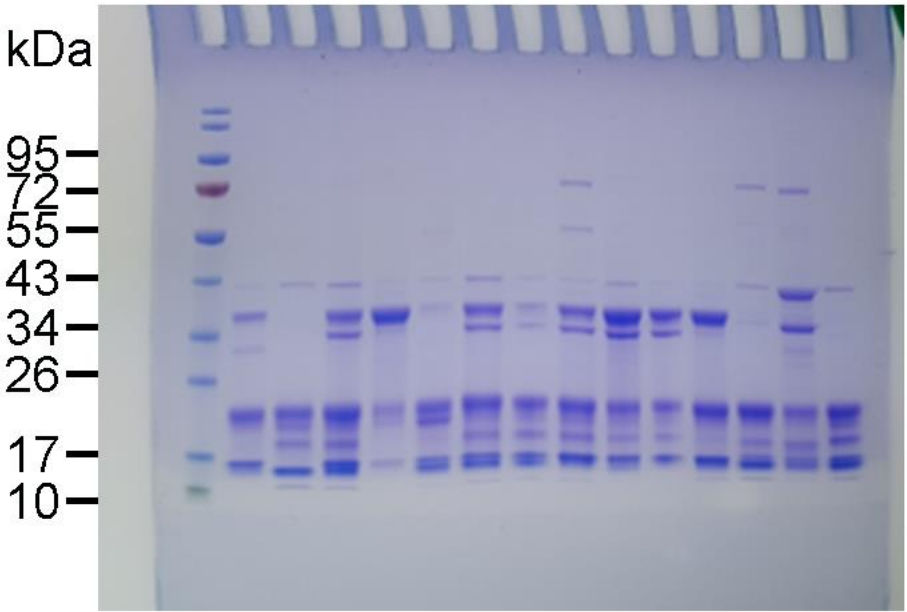

**anti-His**

kDa

34—

10—

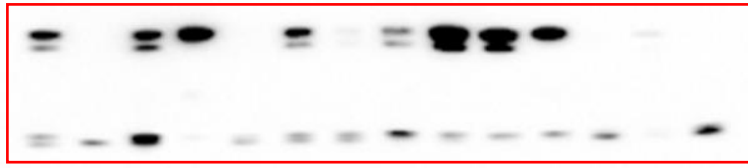

Supplement: Figure 4—figure supplement 1—source data 2. [file elife-89974-fig4-figsupp1-data2.zip › Figure 4-figure supplement 1-source data 2/Figure 4-figure supplement 1C-source data.pdf]

Figure 4-figure supplement 1D

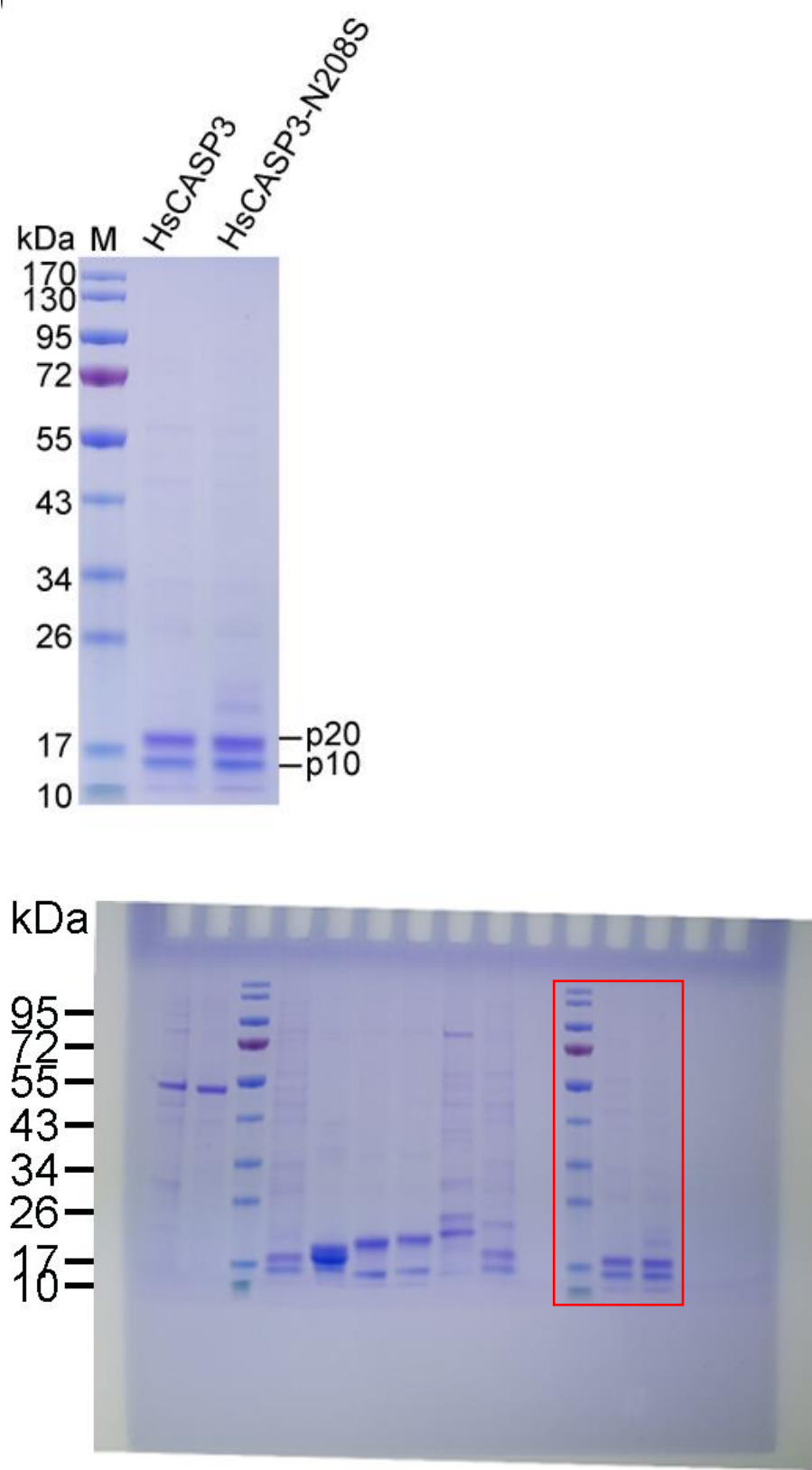

Supplement: Figure 4—figure supplement 1—source data 2. [file elife-89974-fig4-figsupp1-data2.zip › Figure 4-figure supplement 1-source data 2/Figure 4-figure supplement 1D-source data.pdf]

Figure 4-figure supplement 1E

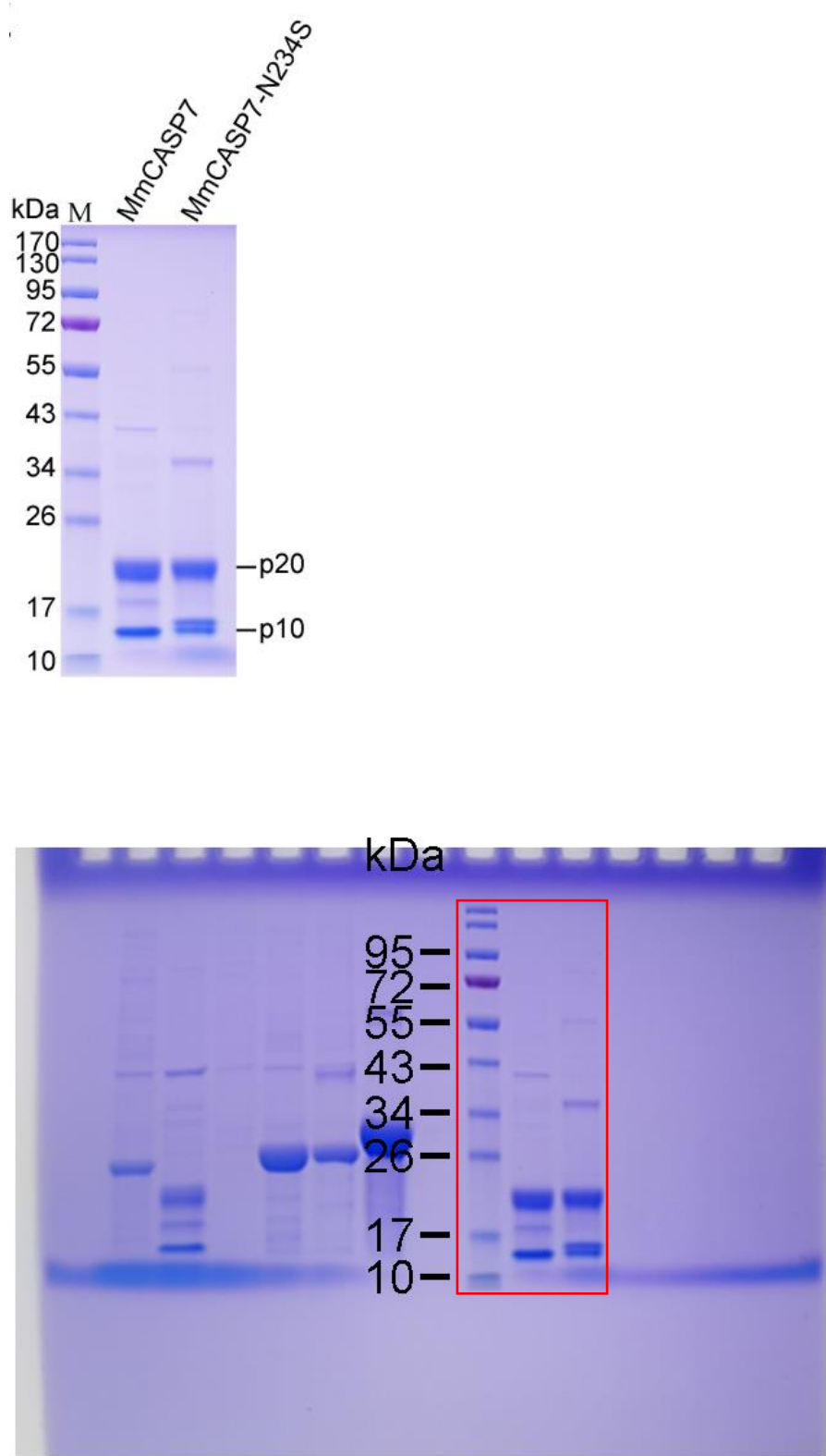

Supplement: Figure 4—figure supplement 1—source data 2. [file elife-89974-fig4-figsupp1-data2.zip › Figure 4-figure supplement 1-source data 2/Figure 4-figure supplement 1E-source data.pdf]

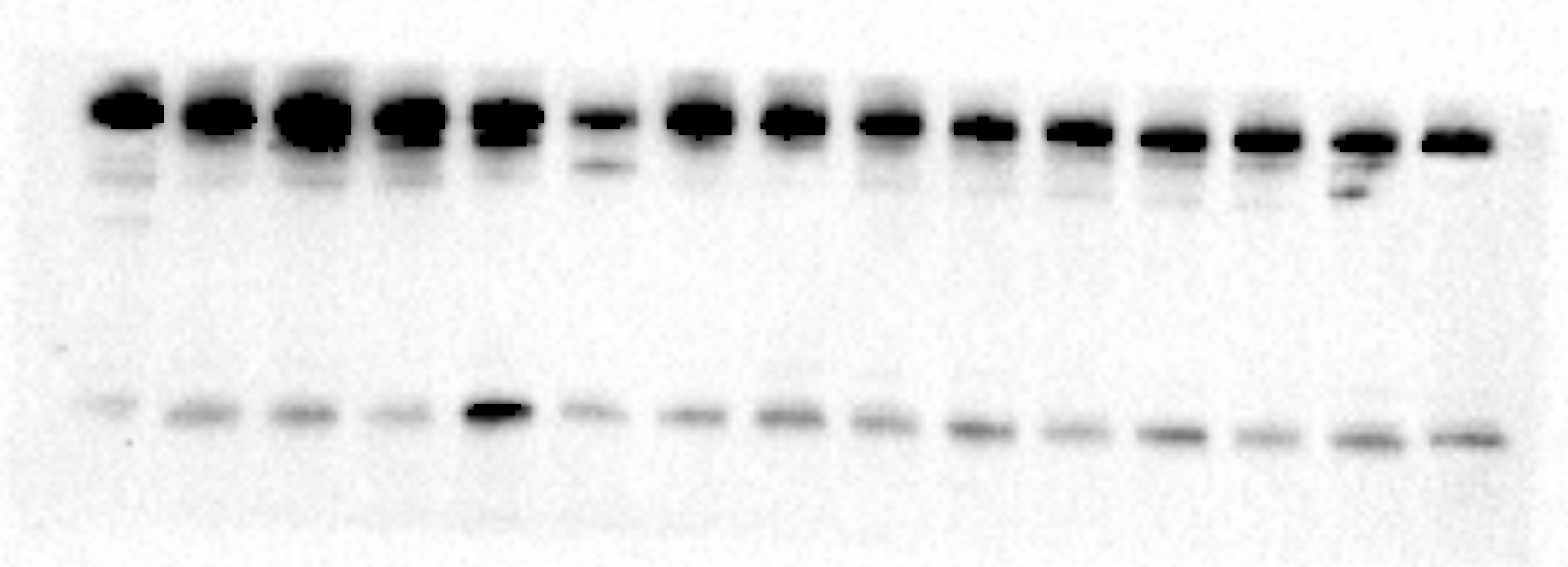

Supplement: Figure 5—source data 1. [file elife-89974-fig5-data1.zip › Figure 5-source data 1/Figure 5B-source data.tif]

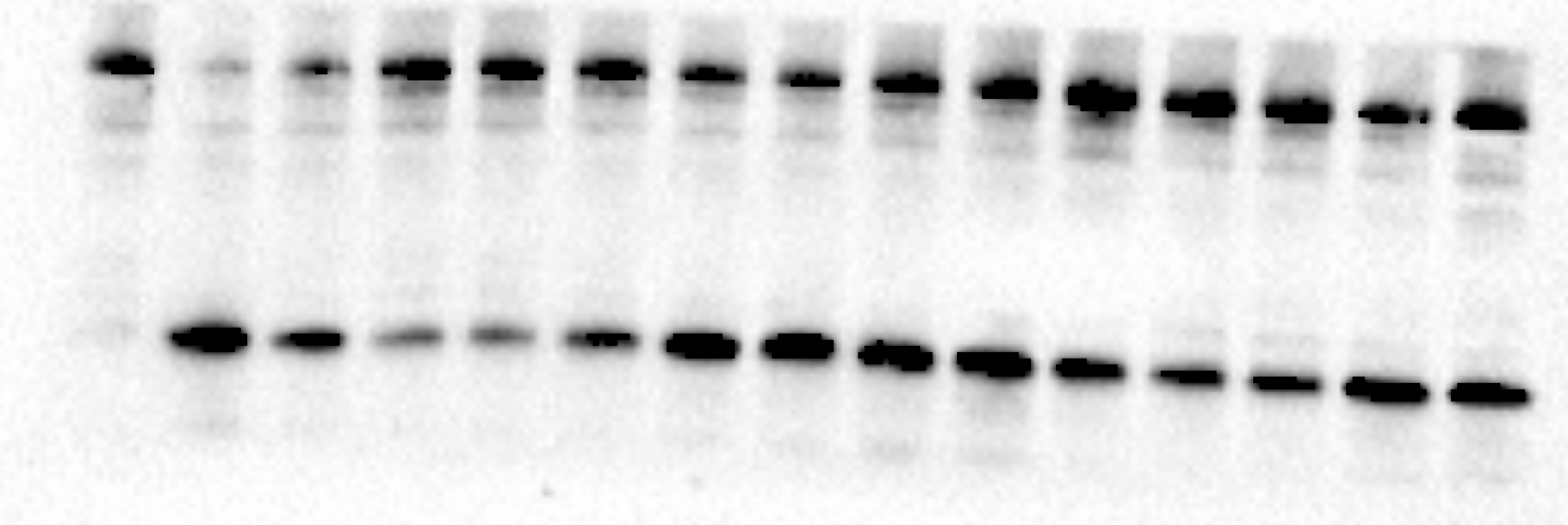

Supplement: Figure 5—source data 1. [file elife-89974-fig5-data1.zip › Figure 5-source data 1/Figure 5C-source data.tif]

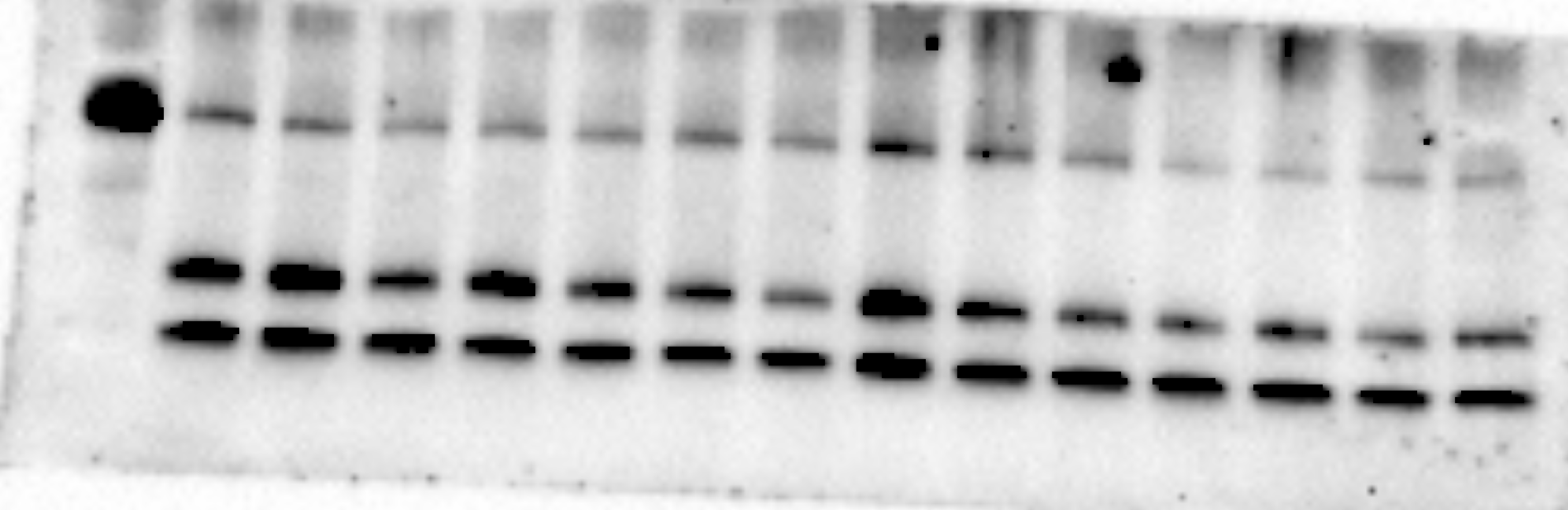

Supplement: Figure 5—source data 1. [file elife-89974-fig5-data1.zip › Figure 5-source data 1/Figure 5D-source data.tif]

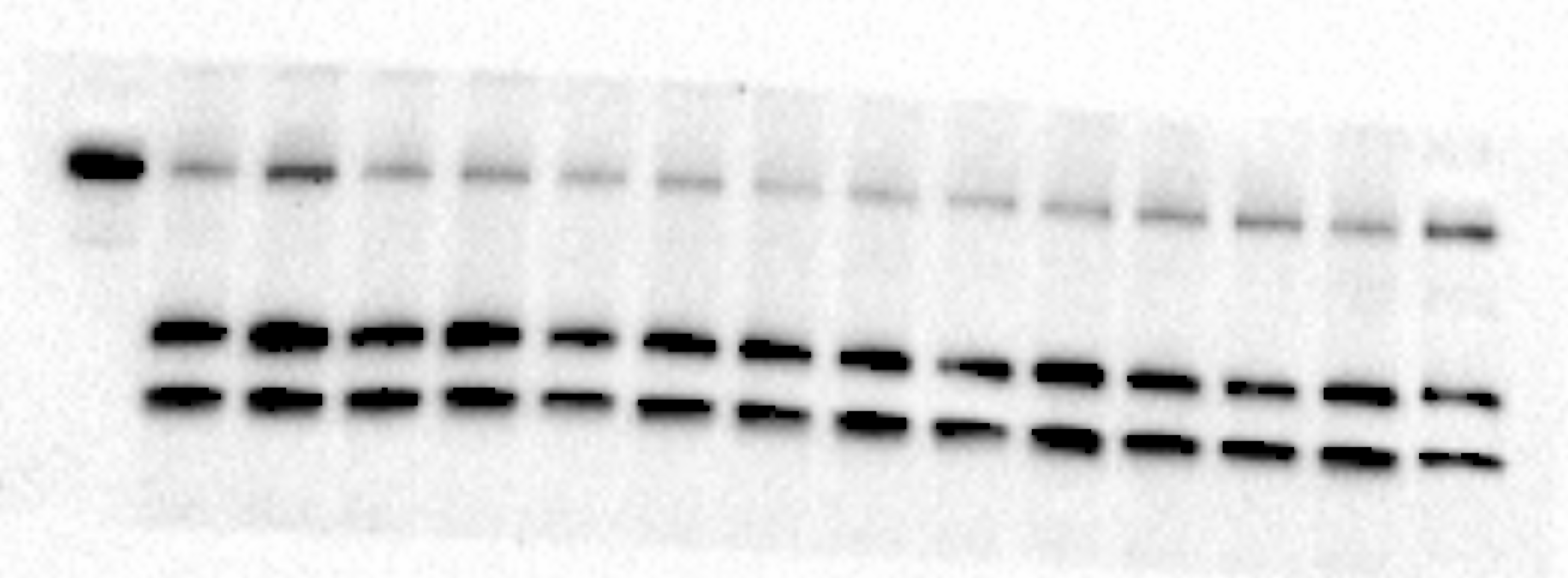

Supplement: Figure 5—source data 1. [file elife-89974-fig5-data1.zip › Figure 5-source data 1/Figure 5E-source data.tif]

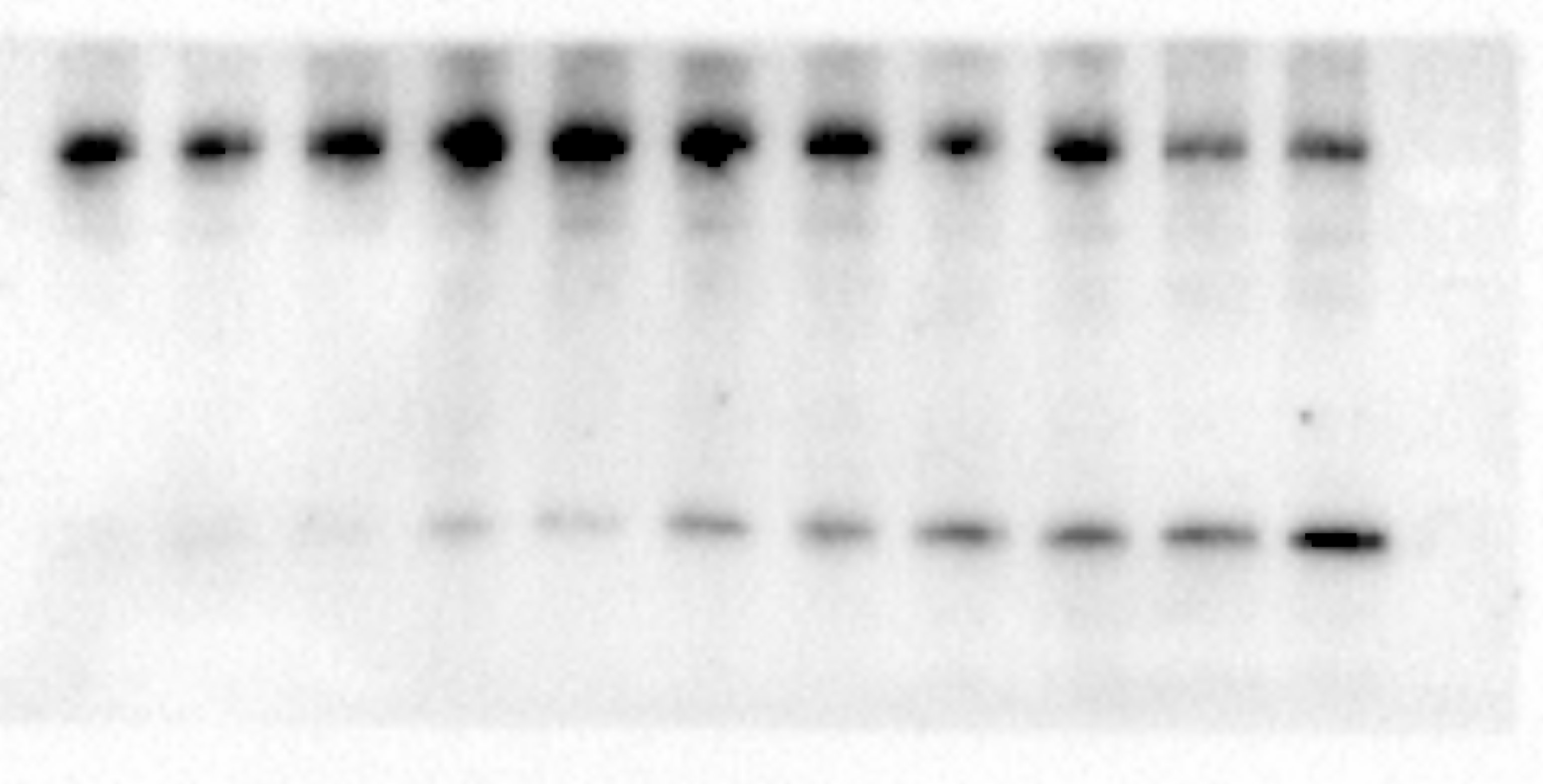

Supplement: Figure 5—source data 1. [file elife-89974-fig5-data1.zip › Figure 5-source data 1/Figure 5F-source data.tif]

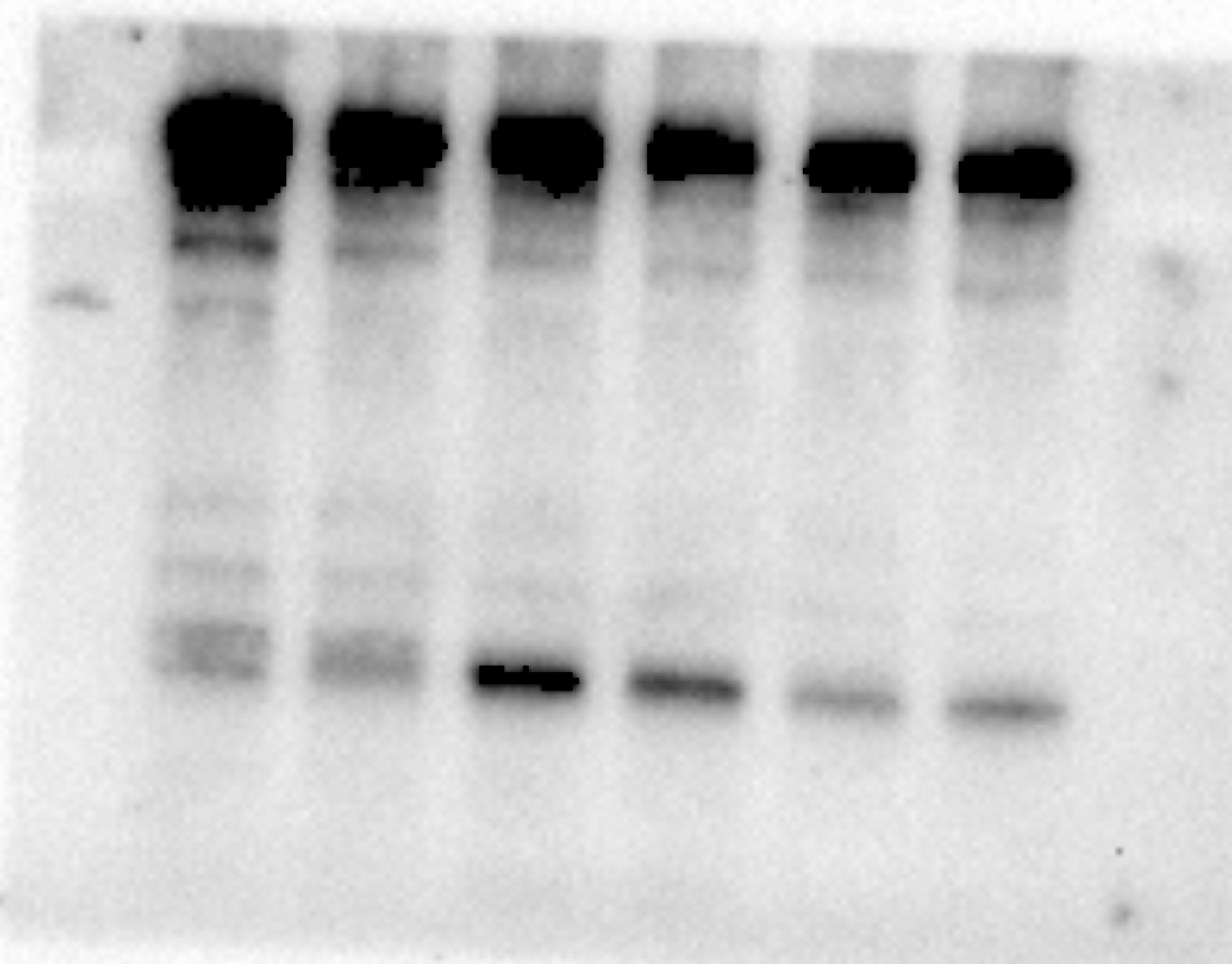

Supplement: Figure 5—source data 1. [file elife-89974-fig5-data1.zip › Figure 5-source data 1/Figure 5G-source data.tif]

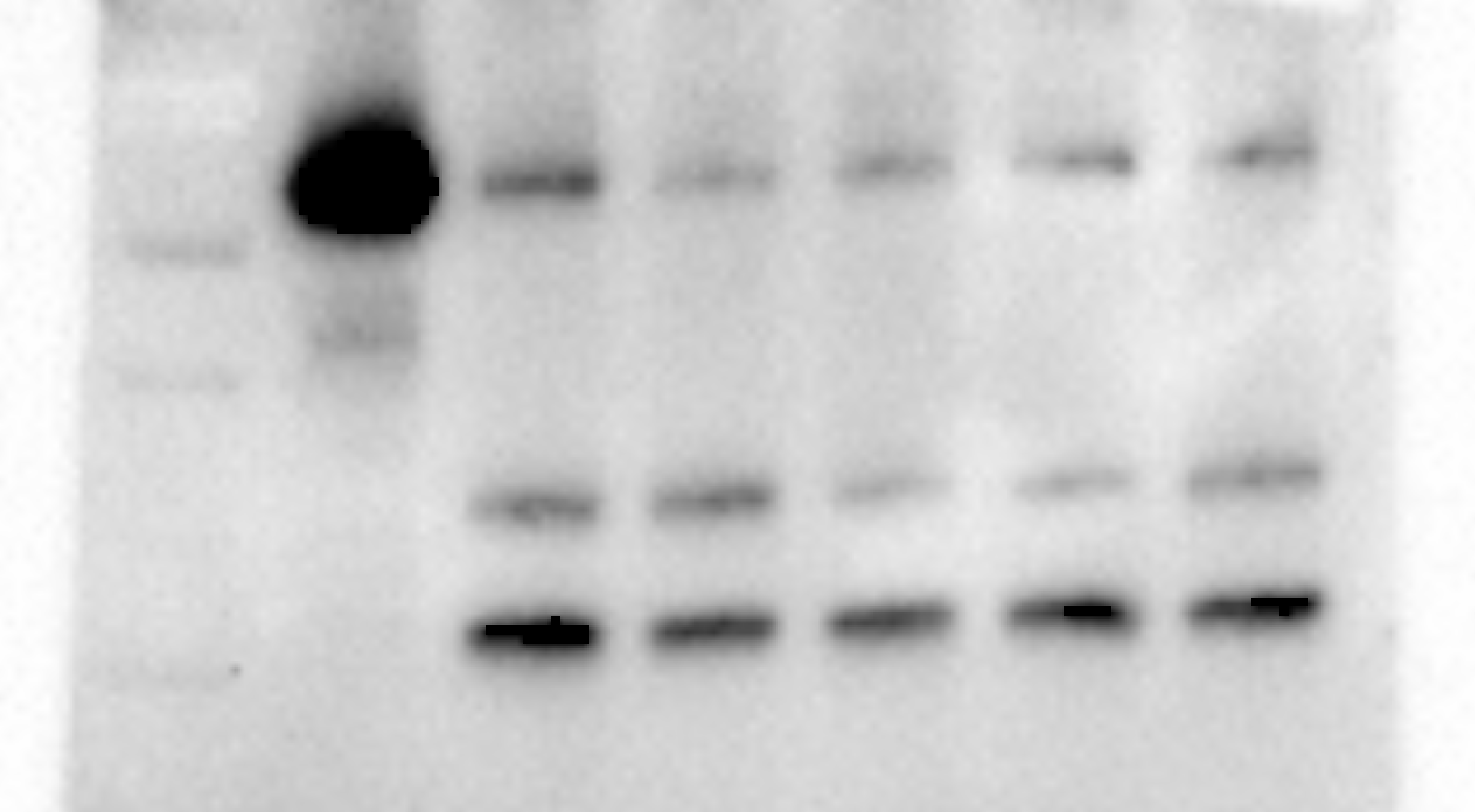

Supplement: Figure 5—source data 1. [file elife-89974-fig5-data1.zip › Figure 5-source data 1/Figure 5H-source data.tif]

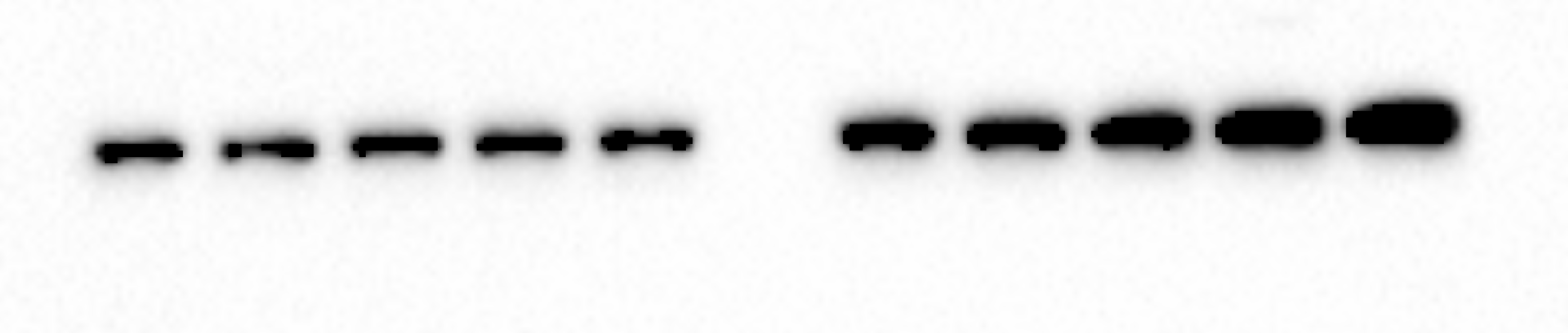

Supplement: Figure 5—source data 1. [file elife-89974-fig5-data1.zip › Figure 5-source data 1/Figure 5I-source data (anti-actin).tif]

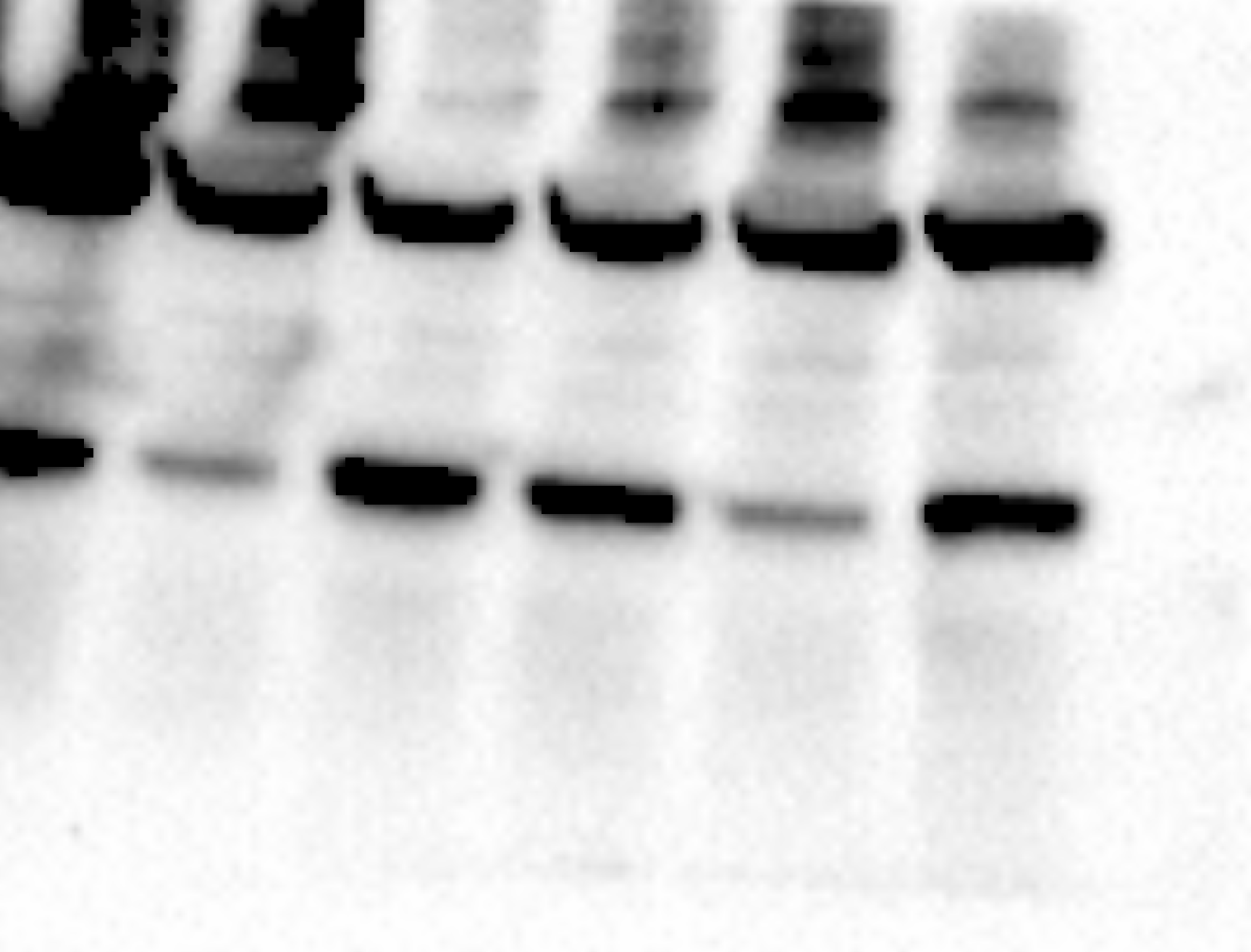

Supplement: Figure 5—source data 1. [file elife-89974-fig5-data1.zip › Figure 5-source data 1/Figure 5I-source data (anti-flag).tif]

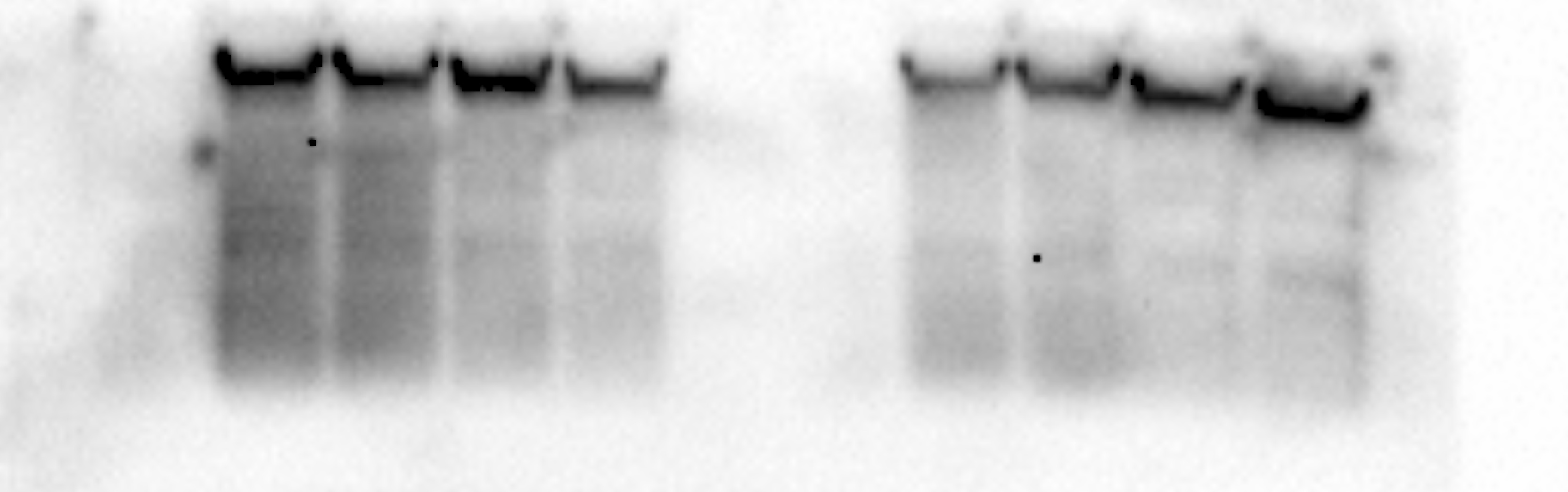

Supplement: Figure 5—source data 1. [file elife-89974-fig5-data1.zip › Figure 5-source data 1/Figure 5I-source data (anti-myc).tif]

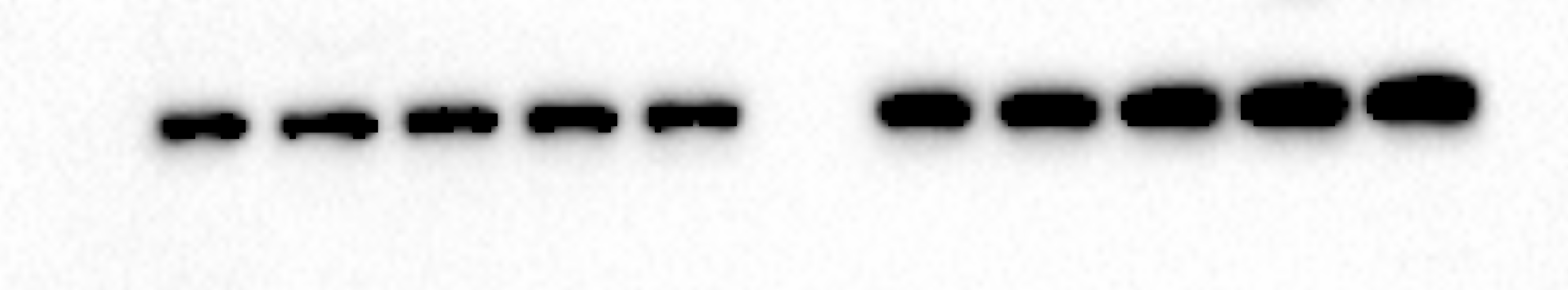

Supplement: Figure 5—source data 1. [file elife-89974-fig5-data1.zip › Figure 5-source data 1/Figure 5J-source data (anti-actin).tif]

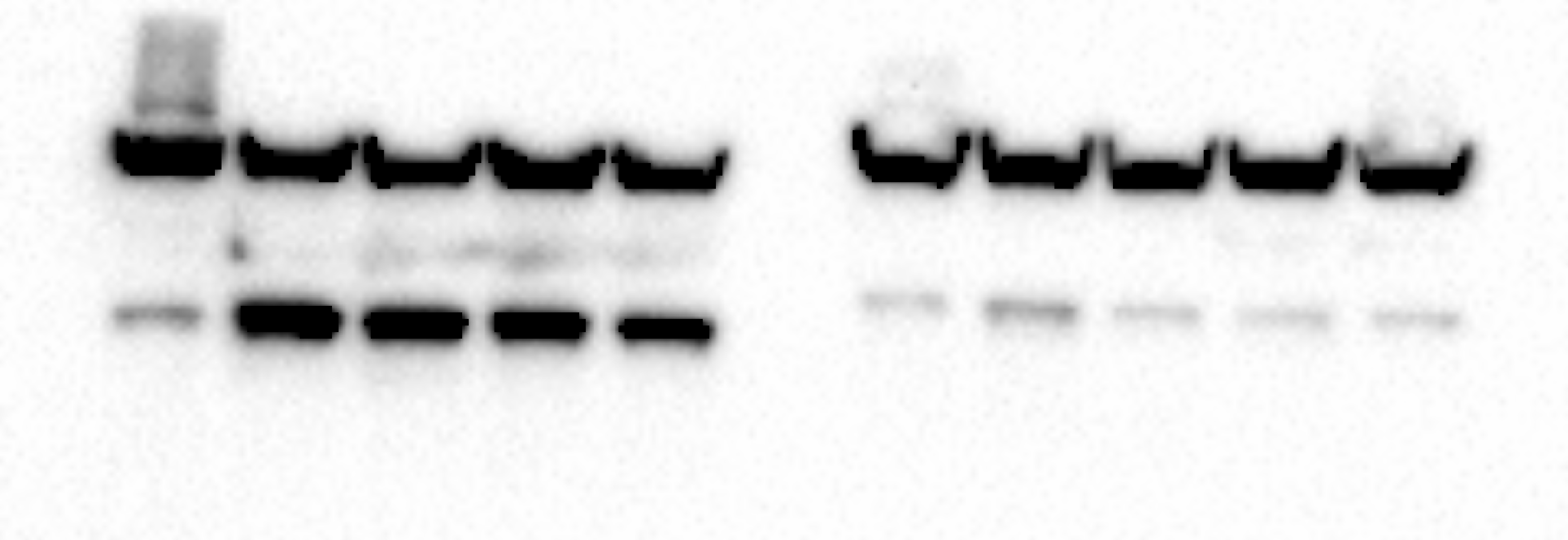

Supplement: Figure 5—source data 1. [file elife-89974-fig5-data1.zip › Figure 5-source data 1/Figure 5J-source data (anti-flag).tif]

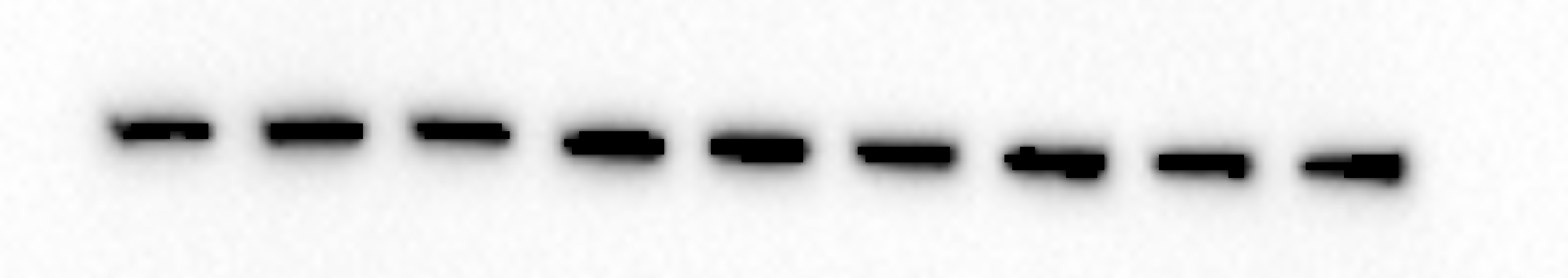

Supplement: Figure 5—source data 1. [file elife-89974-fig5-data1.zip › Figure 5-source data 1/Figure 5K-source data (anti-actin).tif]

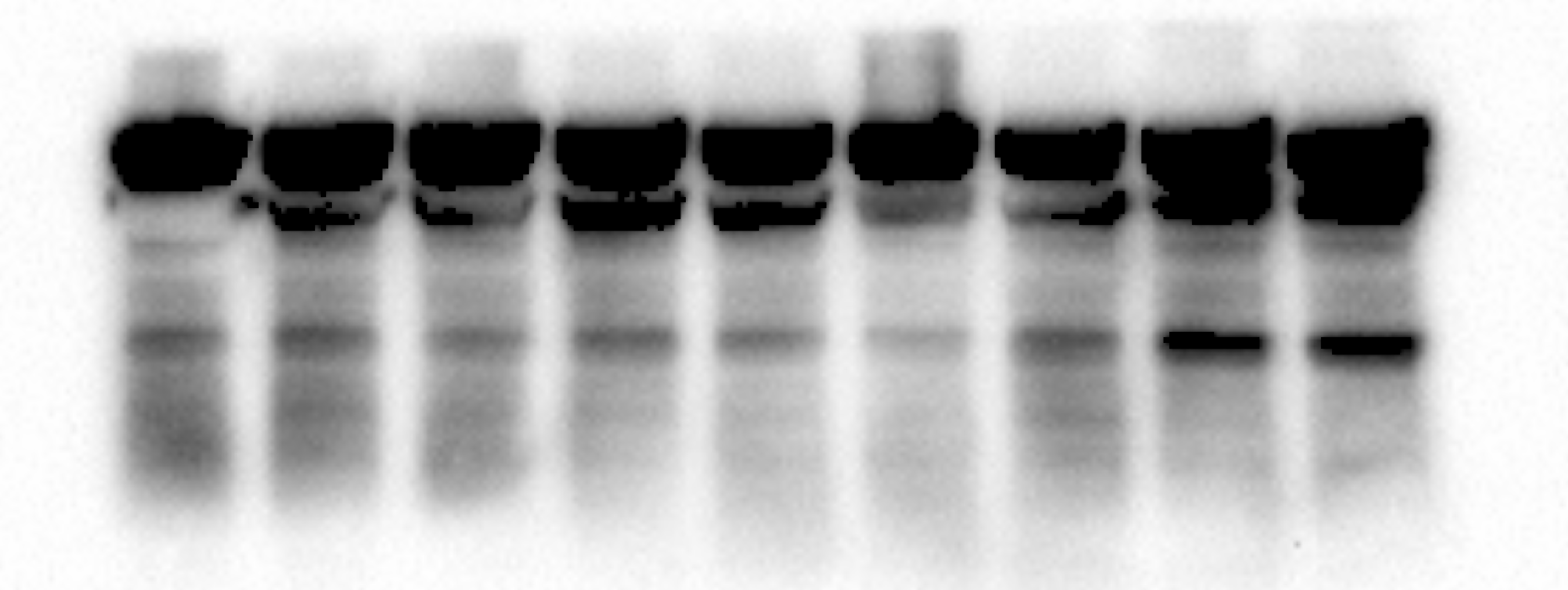

Supplement: Figure 5—source data 1. [file elife-89974-fig5-data1.zip › Figure 5-source data 1/Figure 5K-source data (anti-flag).tif]

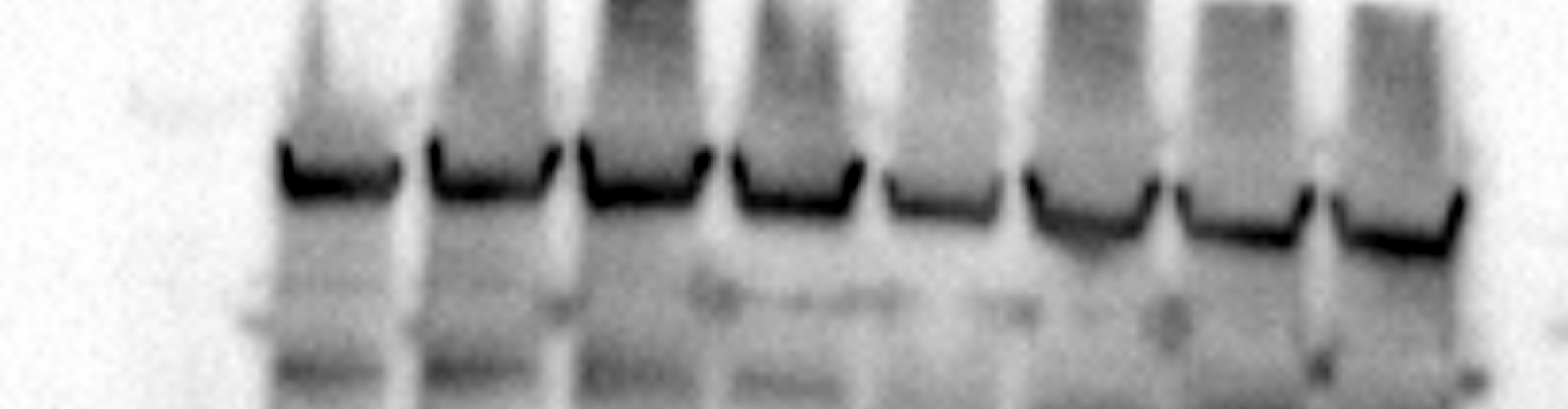

Supplement: Figure 5—source data 1. [file elife-89974-fig5-data1.zip › Figure 5-source data 1/Figure 5K-source data (anti-myc).tif]

**Figure 5B**

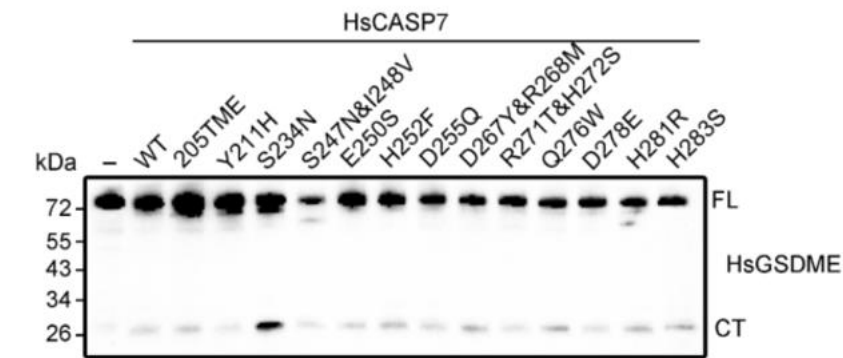

**anti-HsGSDME-CT**

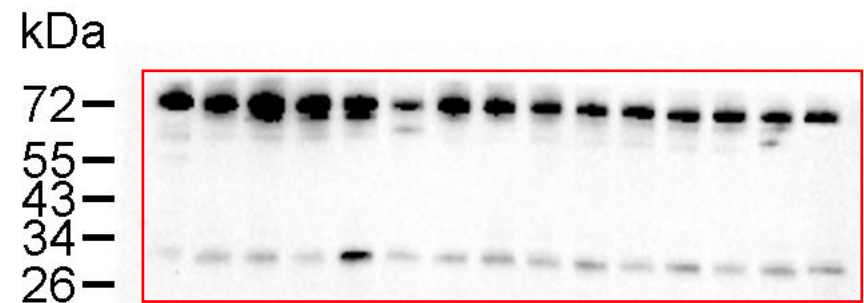

Supplement: Figure 5—source data 2. [file elife-89974-fig5-data2.zip › Figure 5-source data 2/Figure 5B-source data.pdf]

**Figure 5C**

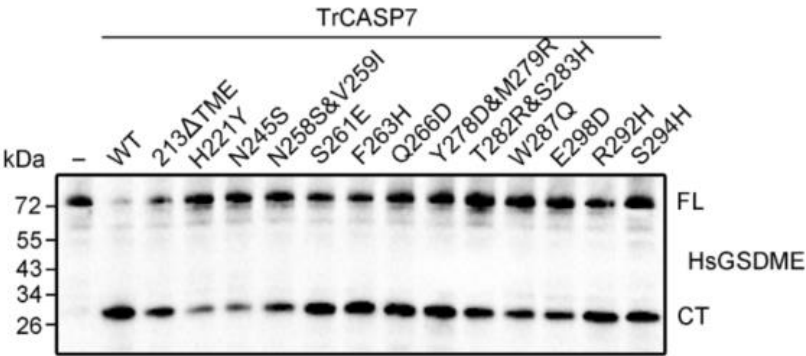

**anti-HsGSDME-CT**

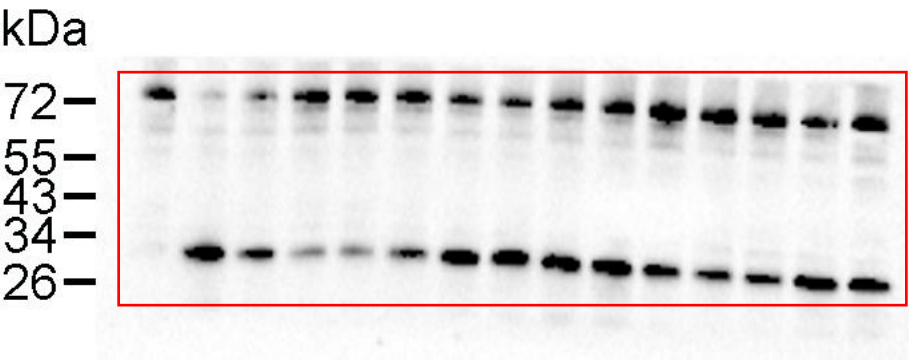

Supplement: Figure 5—source data 2. [file elife-89974-fig5-data2.zip › Figure 5-source data 2/Figure 5C-source data.pdf]

**Figure 5D**

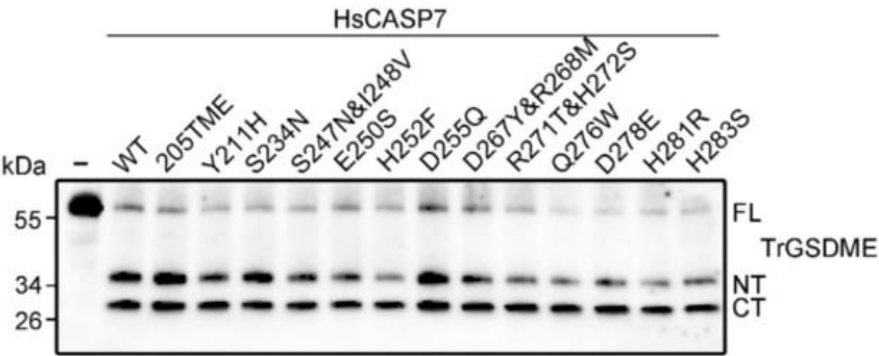

**anti-TrGSDME**

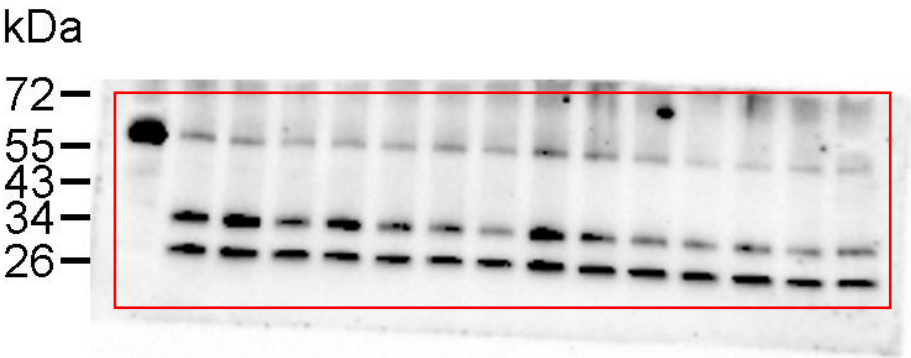

Supplement: Figure 5—source data 2. [file elife-89974-fig5-data2.zip › Figure 5-source data 2/Figure 5D-source data.pdf]

**Figure 5E**

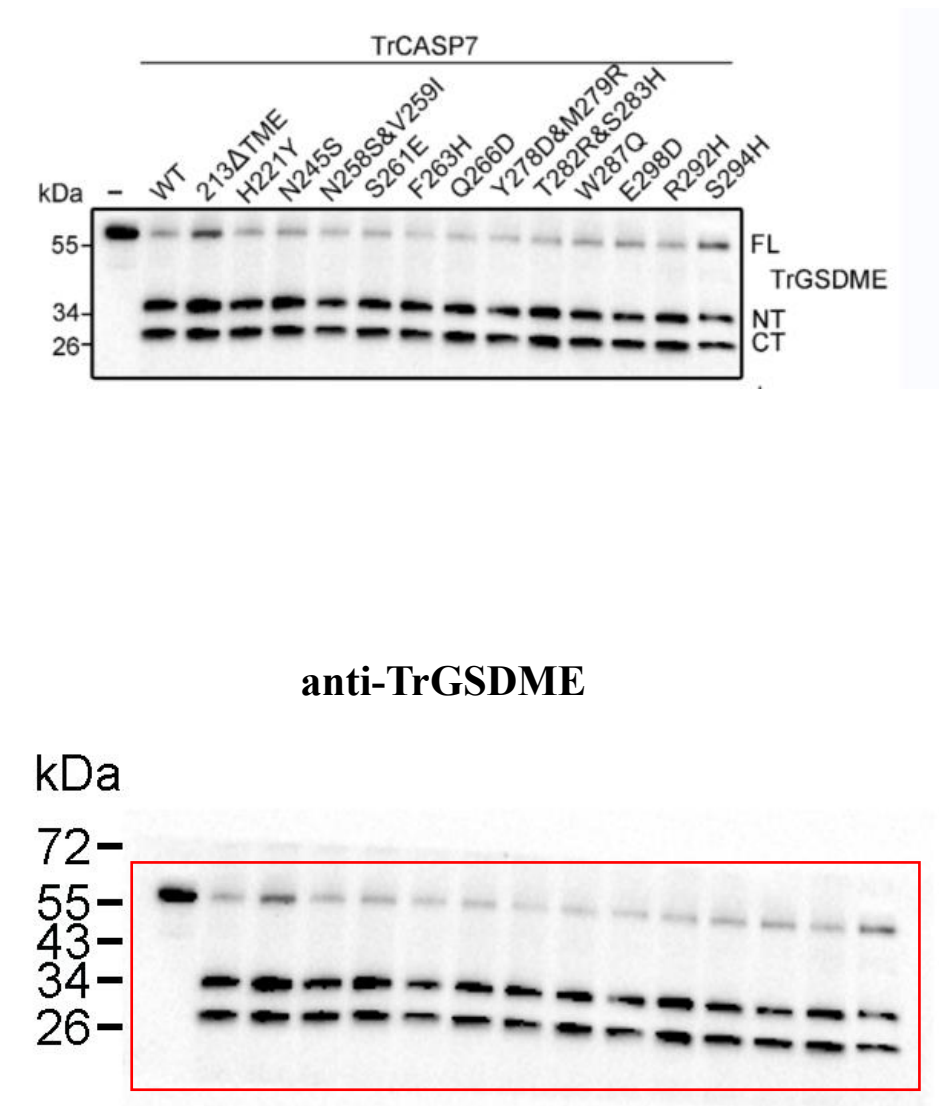

Supplement: Figure 5—source data 2. [file elife-89974-fig5-data2.zip › Figure 5-source data 2/Figure 5E-source data.pdf]

**Figure 5F**

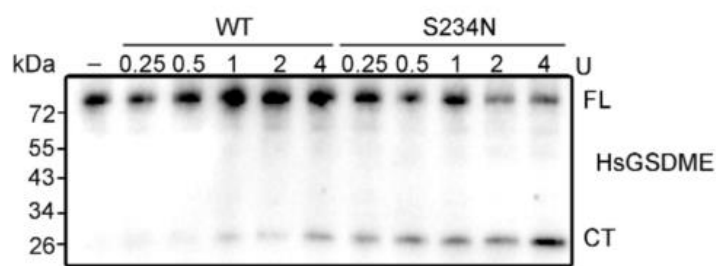

**anti-HsGSDME-CT**

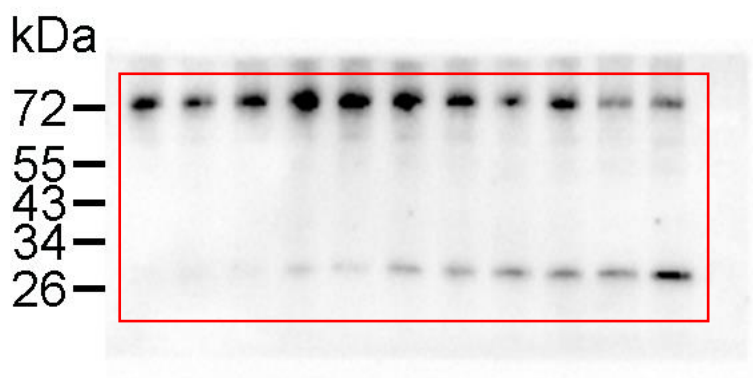

Supplement: Figure 5—source data 2. [file elife-89974-fig5-data2.zip › Figure 5-source data 2/Figure 5F-source data.pdf]

**Figure 5G**

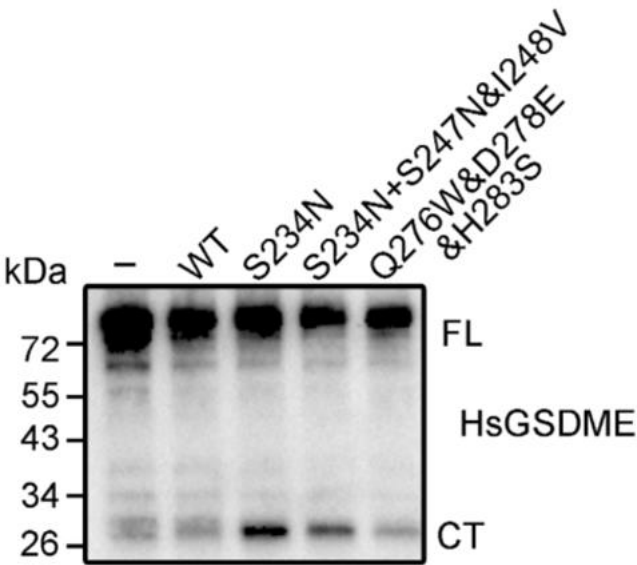

**anti-HsGSDME-CT**

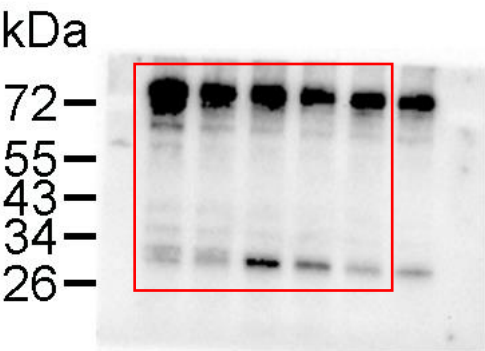

Supplement: Figure 5—source data 2. [file elife-89974-fig5-data2.zip › Figure 5-source data 2/Figure 5G-source data.pdf]

**Figure 5H**

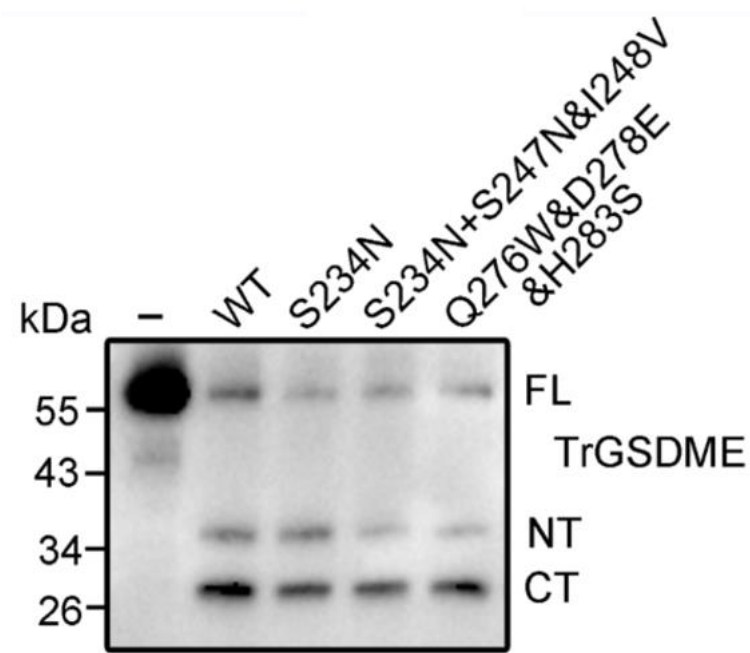

**Anti-TrGSDME**

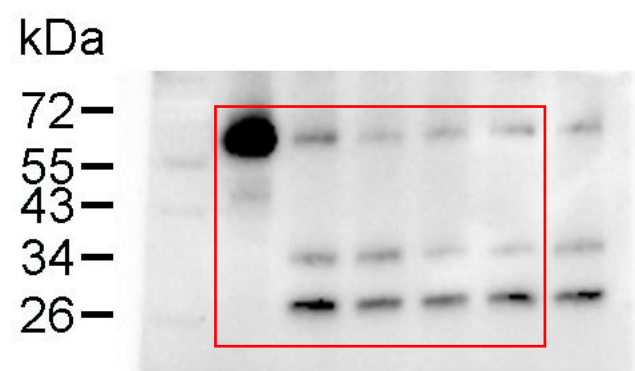

Supplement: Figure 5—source data 2. [file elife-89974-fig5-data2.zip › Figure 5-source data 2/Figure 5H-source data.pdf]

**Figure 5I**

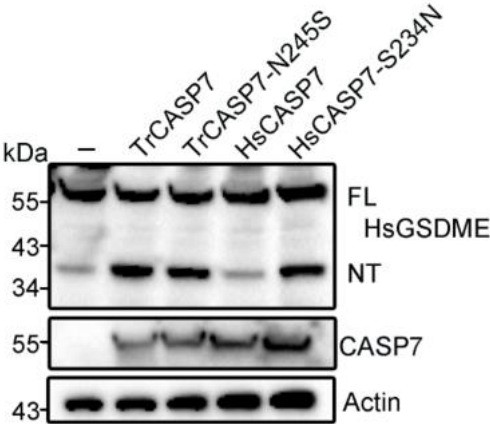

**anti-Flag**

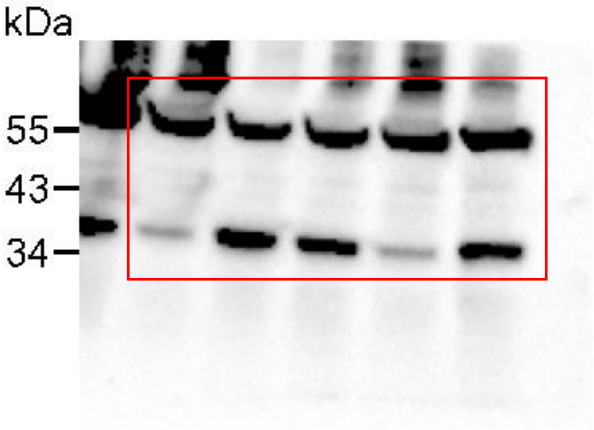

**anti-Myc**

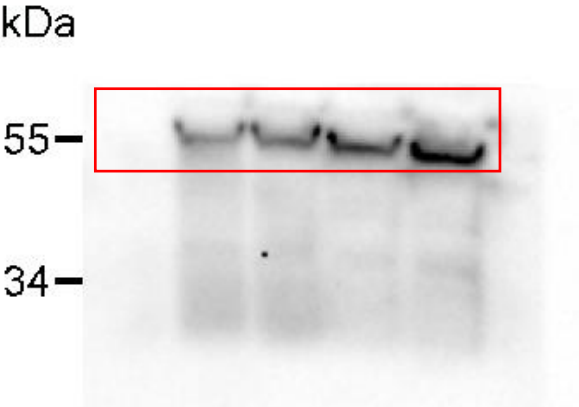

# anti-actin

kDa

43—

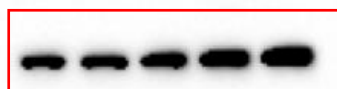

Supplement: Figure 5—source data 2. [file elife-89974-fig5-data2.zip › Figure 5-source data 2/Figure 5I-source data.pdf]

**Figure 5J**

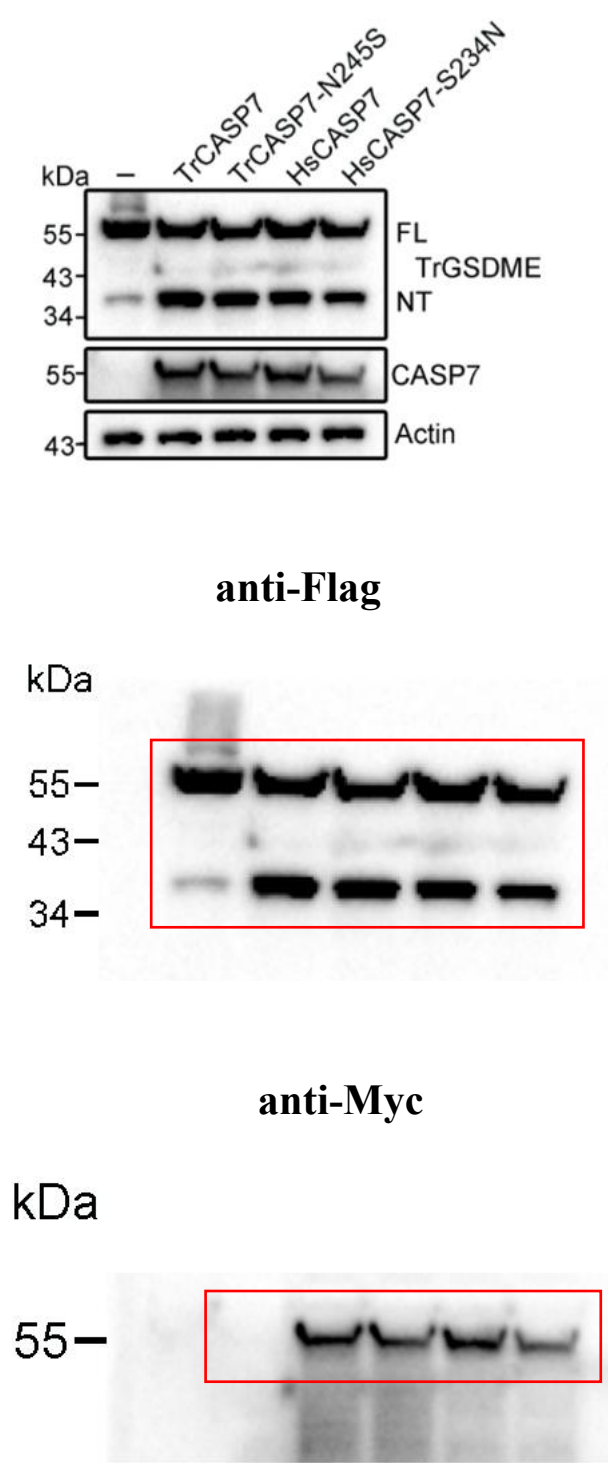

**anti-actin**

kDa

43—

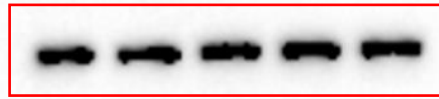

Supplement: Figure 5—source data 2. [file elife-89974-fig5-data2.zip › Figure 5-source data 2/Figure 5J-source data.pdf]

**Figure 5K**

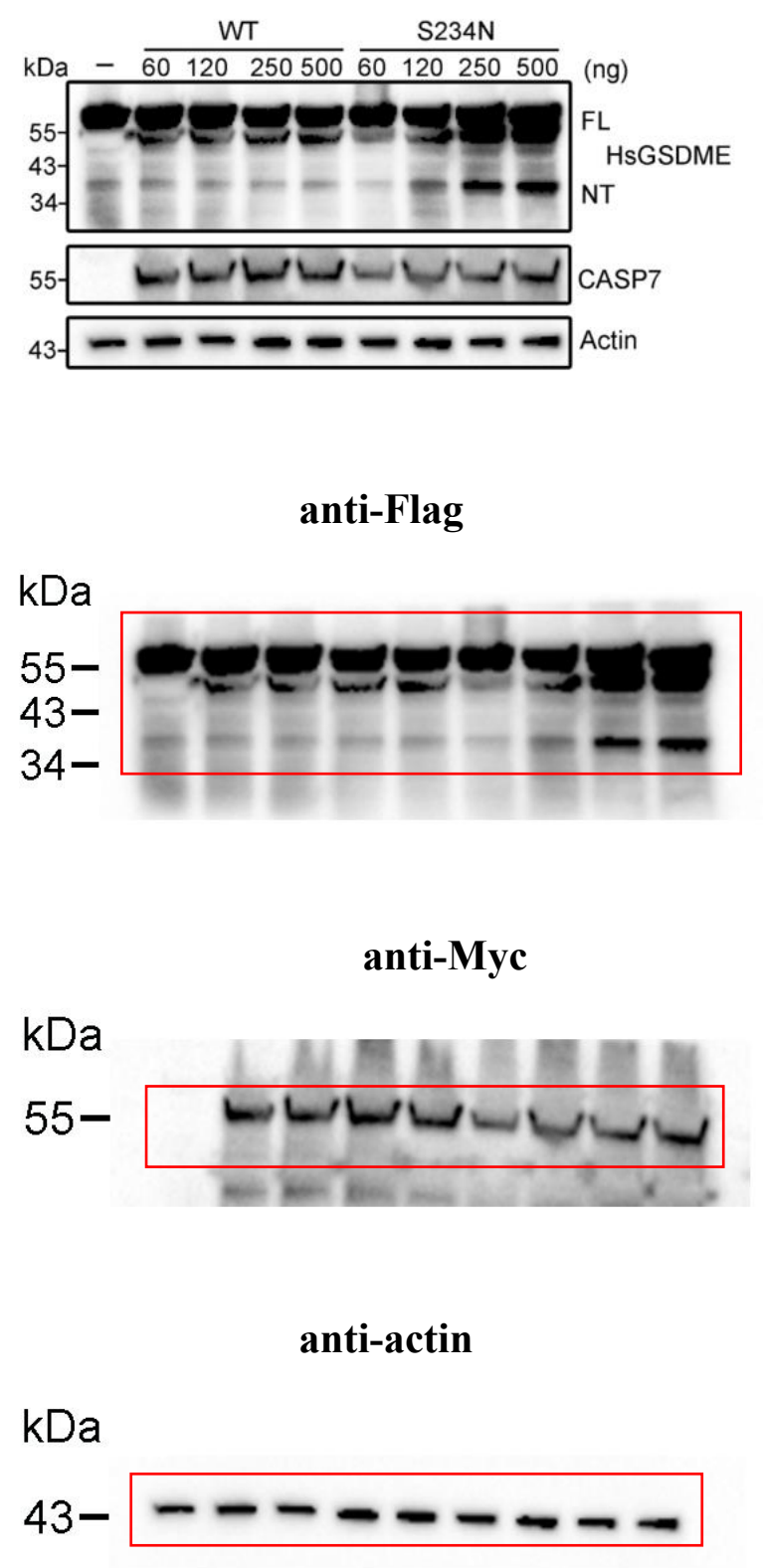

Supplement: Figure 5—source data 2. [file elife-89974-fig5-data2.zip › Figure 5-source data 2/Figure 5K-source data.pdf]

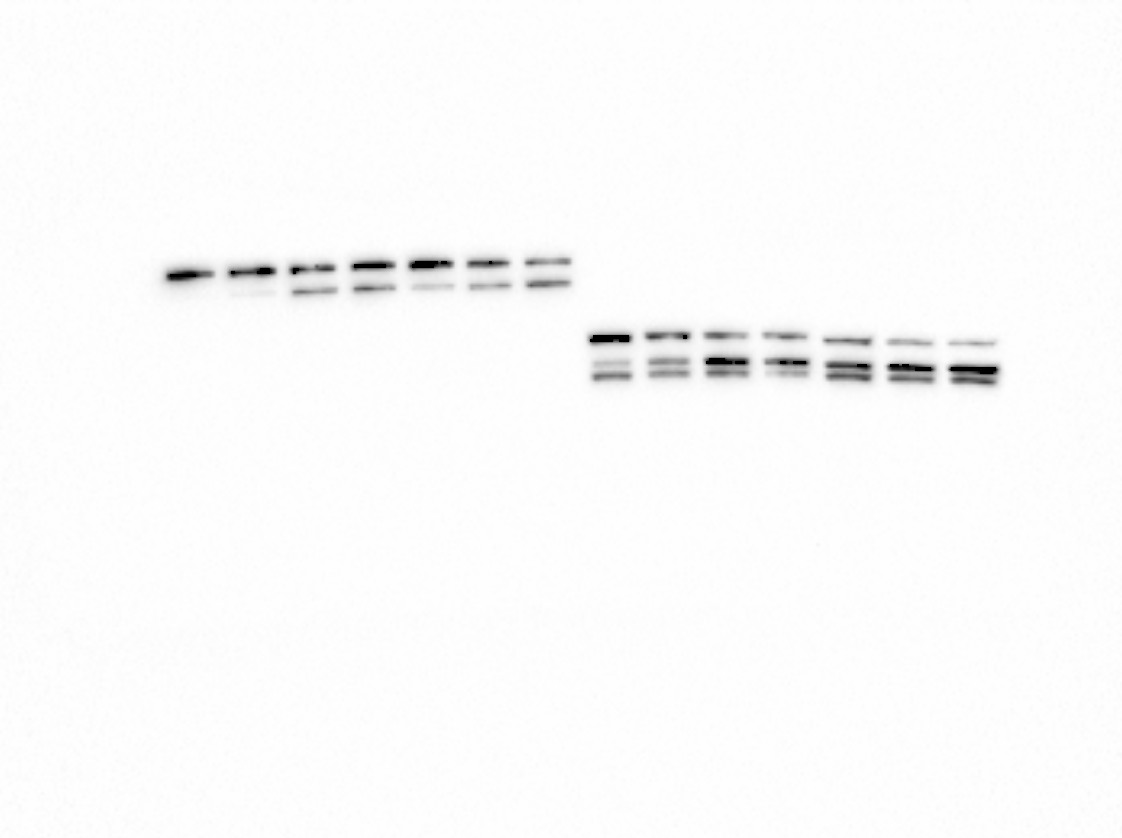

Supplement: Figure 5—figure supplement 1—source data 1. [file elife-89974-fig5-figsupp1-data1.zip › Figure 5-figure supplement 1-source data 1/Figure 5-figure supplement 1-left-source data.tif]

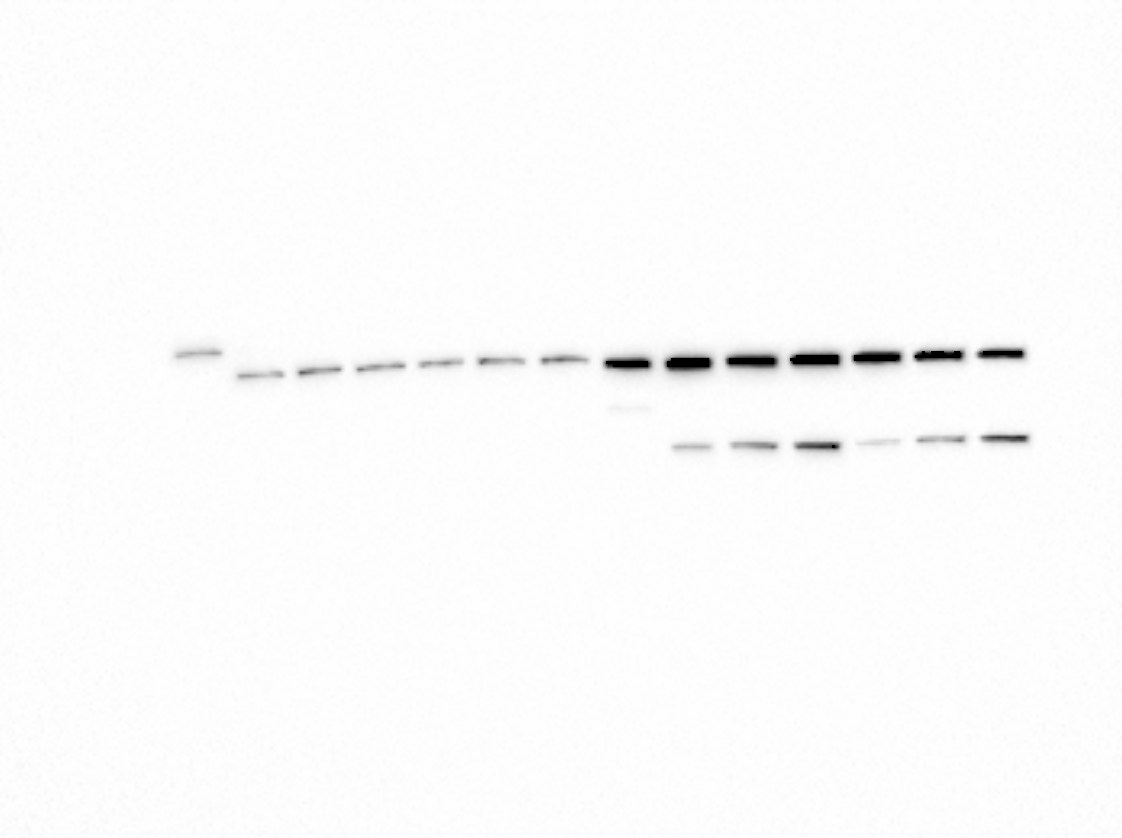

Supplement: Figure 5—figure supplement 1—source data 1. [file elife-89974-fig5-figsupp1-data1.zip › Figure 5-figure supplement 1-source data 1/Figure 5-figure supplement 1-right-source data.tif]

**Figure 5-figure supplement 1-left**

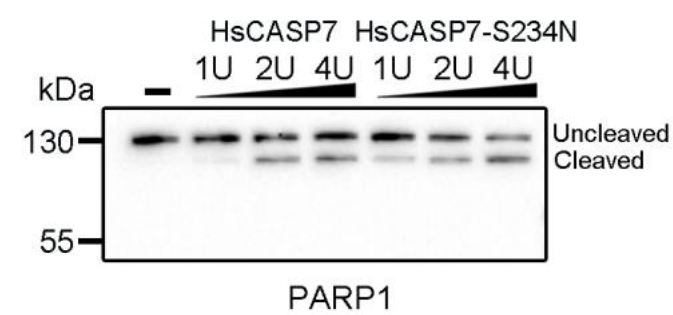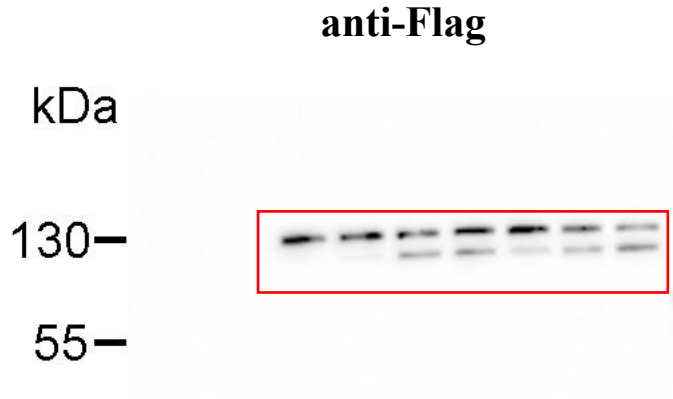

Supplement: Figure 5—figure supplement 1—source data 2. [file elife-89974-fig5-figsupp1-data2.zip › Figure 5-figure supplement 1-source data 2/Figure 5-figure supplement 1-left-source data.pdf]

**Figure 5-figure supplement 1-right**

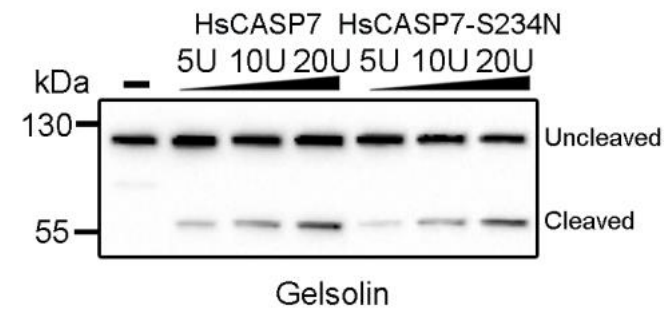

**anti-Flag**

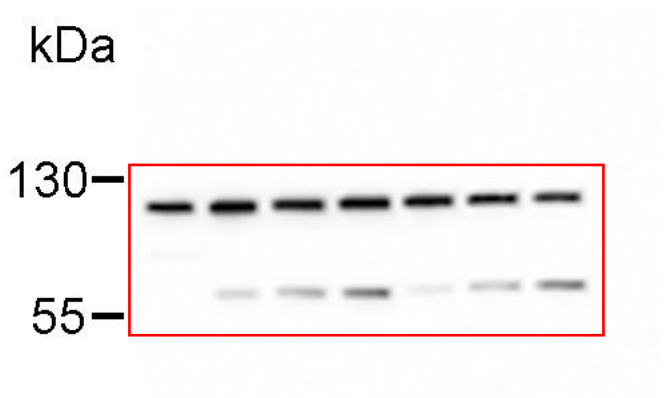

Supplement: Figure 5—figure supplement 1—source data 2. [file elife-89974-fig5-figsupp1-data2.zip › Figure 5-figure supplement 1-source data 2/Figure 5-figure supplement 1-right-source data.pdf]

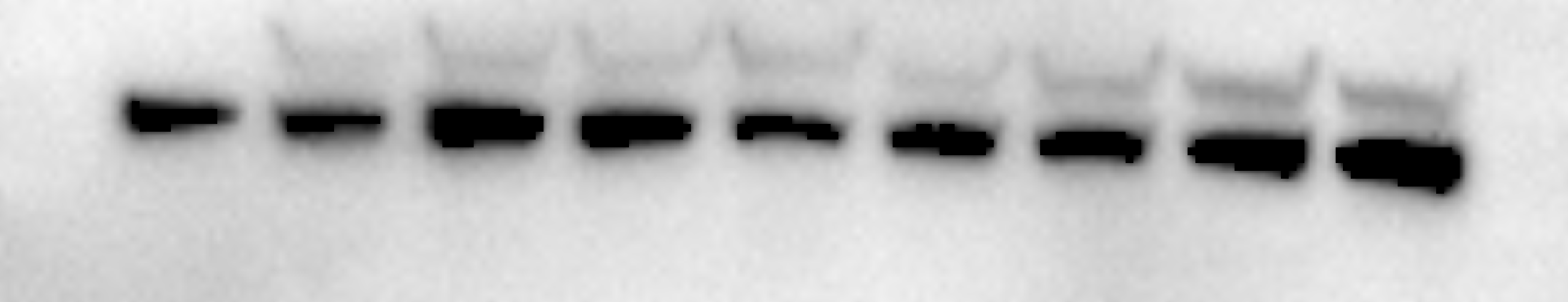

Supplement: Figure 5—figure supplement 3—source data 1. [file elife-89974-fig5-figsupp3-data1.zip › Figure 5-figure supplement 3-source data 1/Figure 5-figure supplement 3A-source data (anti-actin).tif]
