# Supplementary figures and images for: The molecular mechanism and evolutionary divergence of caspase 3/7-regulated gasdermin E activation (part 2 of 2)
Source: eLife. 2024 Mar 15;12:RP89974. doi: 10.7554/eLife.89974 (PMC10942788; doi:10.7554/eLife.89974)

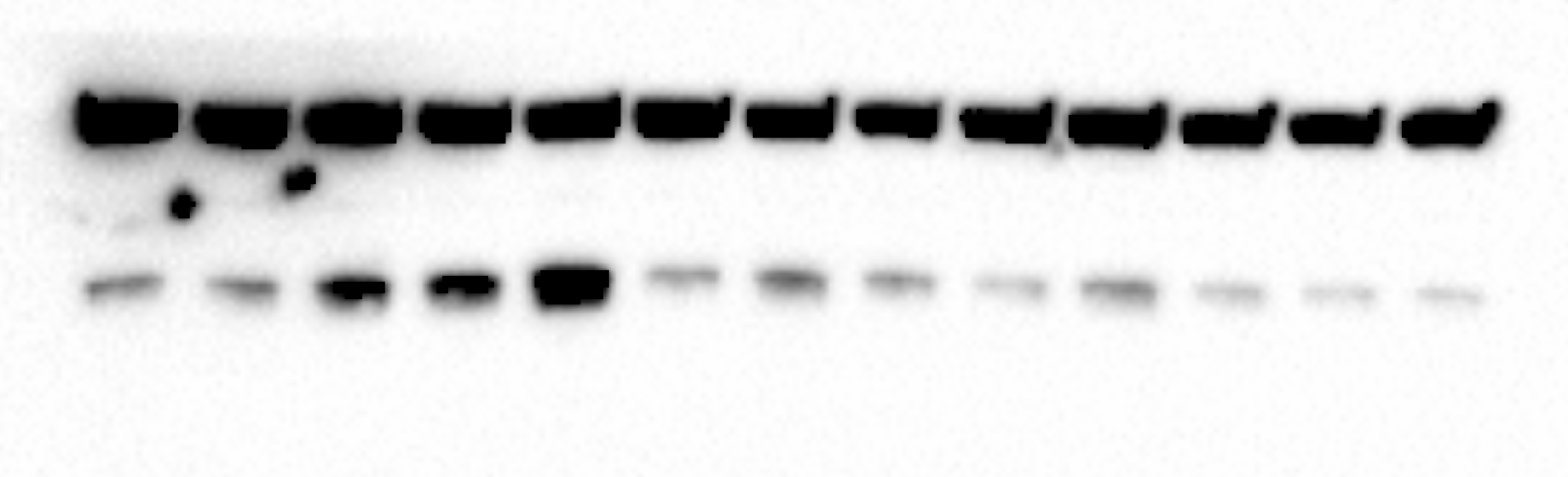

Supplement: Figure 5—figure supplement 3—source data 1. [file elife-89974-fig5-figsupp3-data1.zip › Figure 5-figure supplement 3-source data 1/Figure 5-figure supplement 3A-source data (anti-flag).tif]

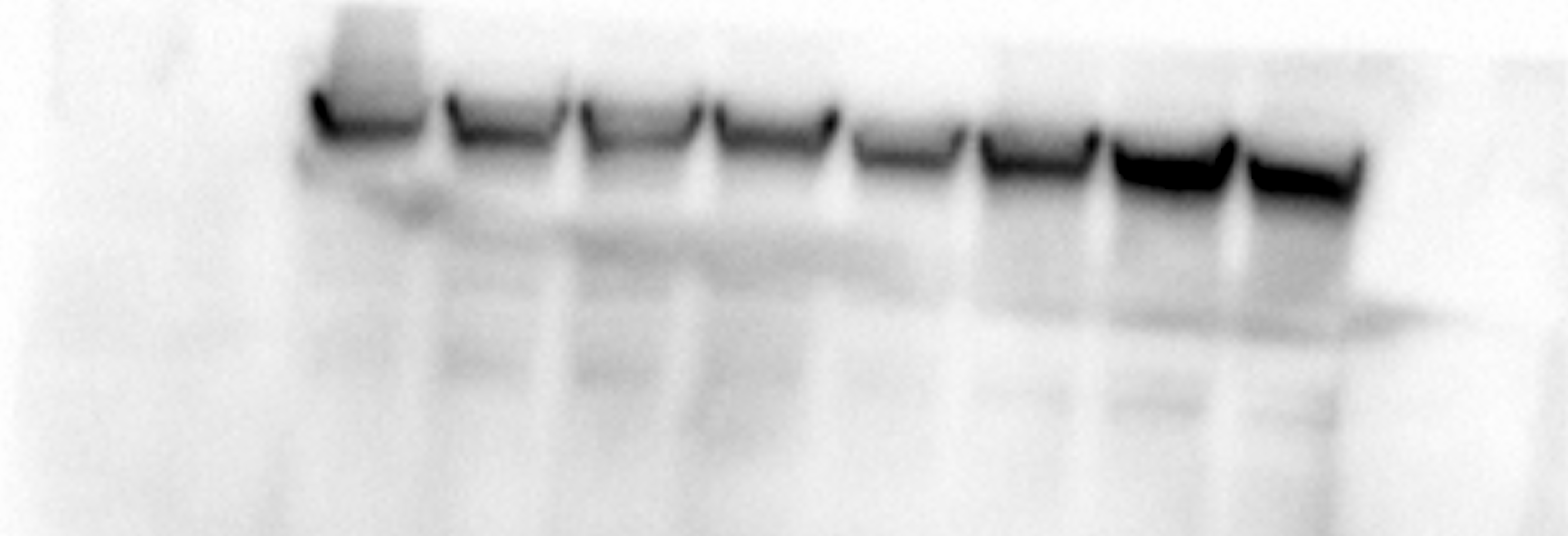

Supplement: Figure 5—figure supplement 3—source data 1. [file elife-89974-fig5-figsupp3-data1.zip › Figure 5-figure supplement 3-source data 1/Figure 5-figure supplement 3A-source data (anti-myc).tif]

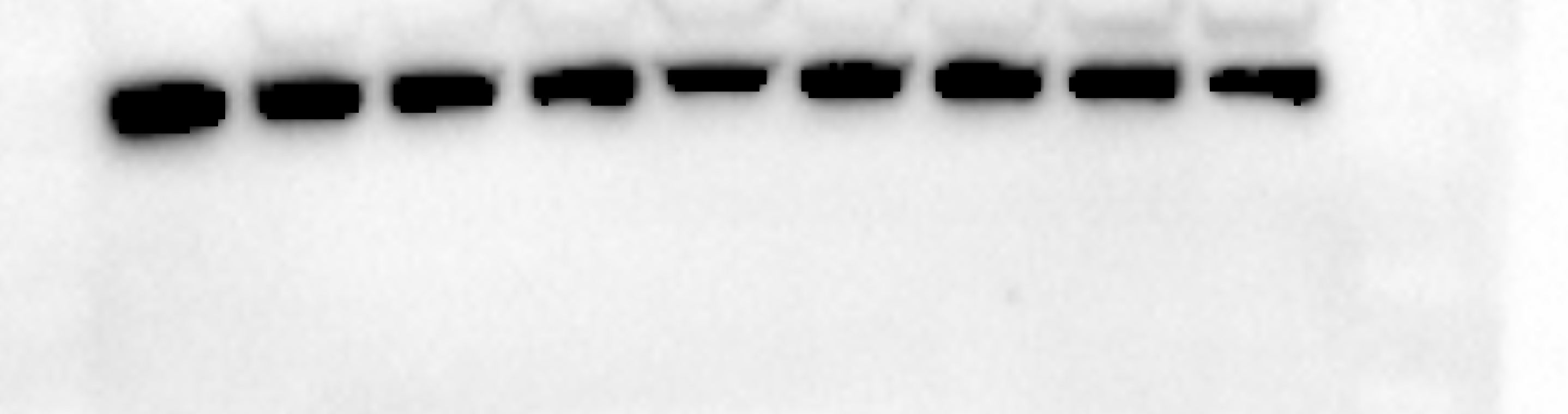

Supplement: Figure 5—figure supplement 3—source data 1. [file elife-89974-fig5-figsupp3-data1.zip › Figure 5-figure supplement 3-source data 1/Figure 5-figure supplement 3B-source data (anti-actin).tif]

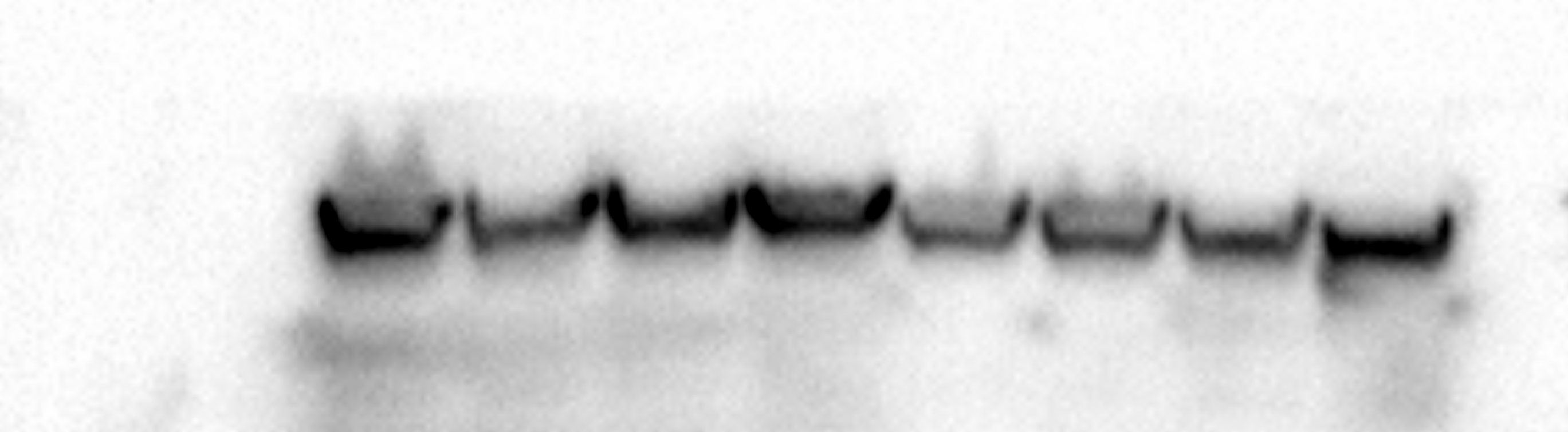

Supplement: Figure 5—figure supplement 3—source data 1. [file elife-89974-fig5-figsupp3-data1.zip › Figure 5-figure supplement 3-source data 1/Figure 5-figure supplement 3B-source data (anti-myc).tif]

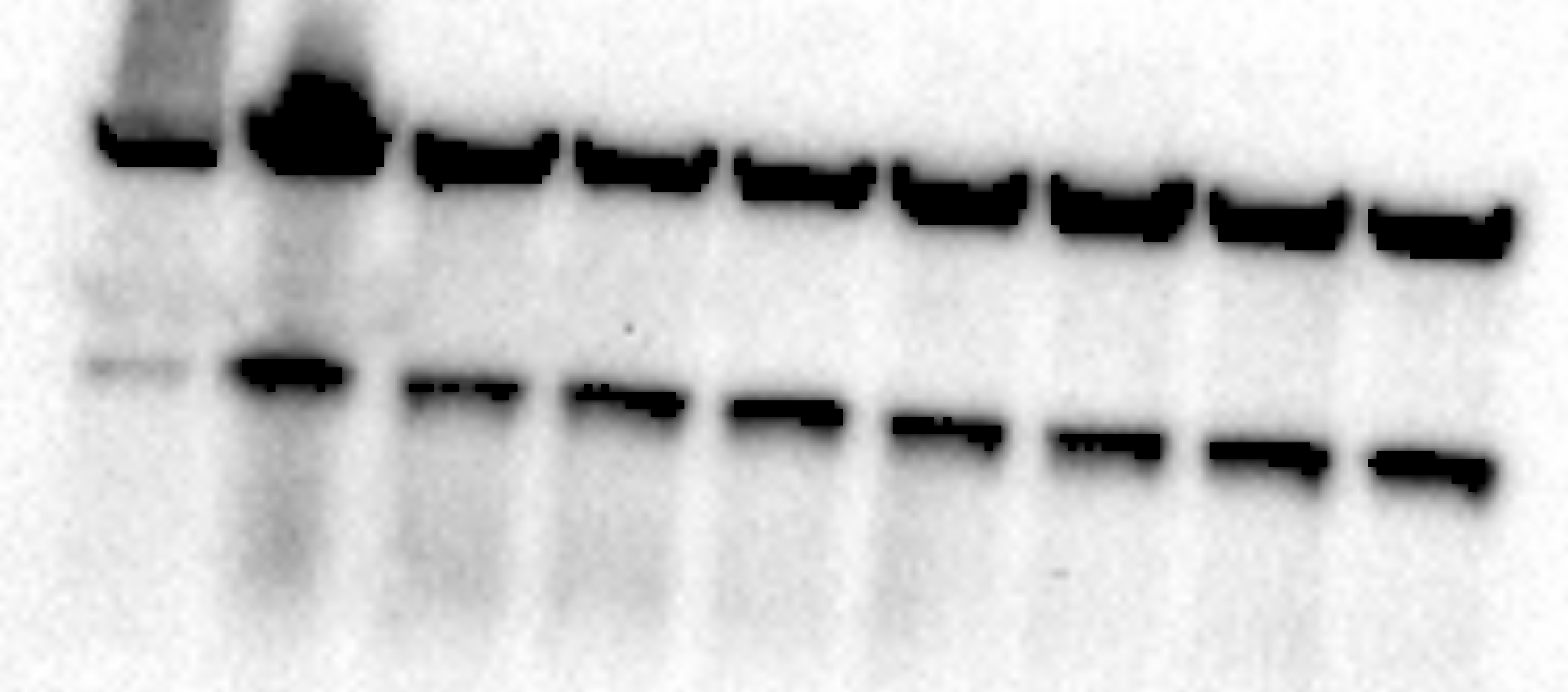

Supplement: Figure 5—figure supplement 3—source data 1. [file elife-89974-fig5-figsupp3-data1.zip › Figure 5-figure supplement 3-source data 1/Figure 5-supplement 3B-source data (anti-flag).tif]

**Figure 5-figure supplement 3A**

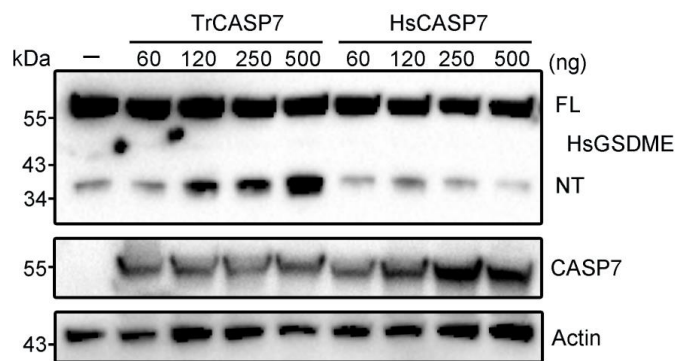

**anti-Flag**

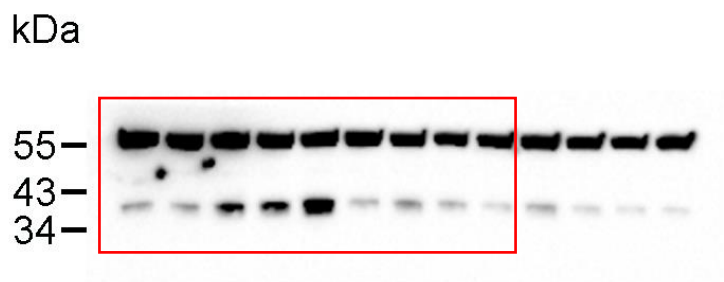

**anti-Myc**

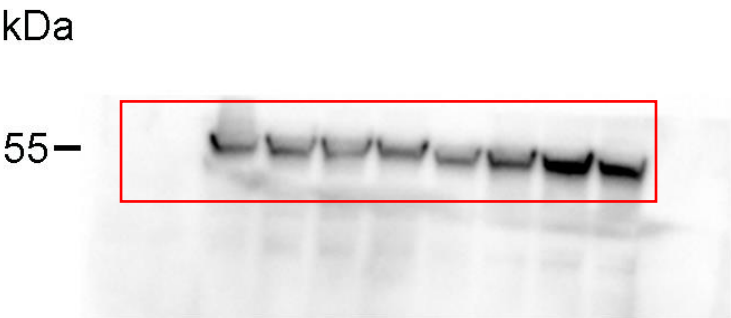

**anti-actin**

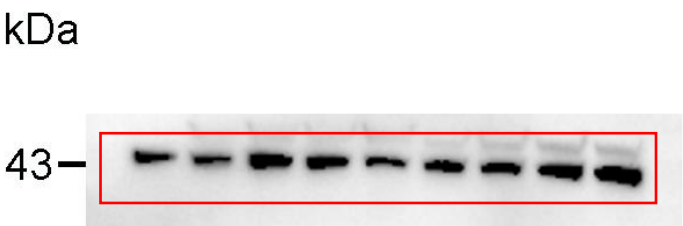

Supplement: Figure 5—figure supplement 3—source data 2. [file elife-89974-fig5-figsupp3-data2.zip › Figure 5-figure supplement 3-source data 2/Figure 5-figure supplement 3A-source data.pdf]

**Figure 5-figure supplement 3B**

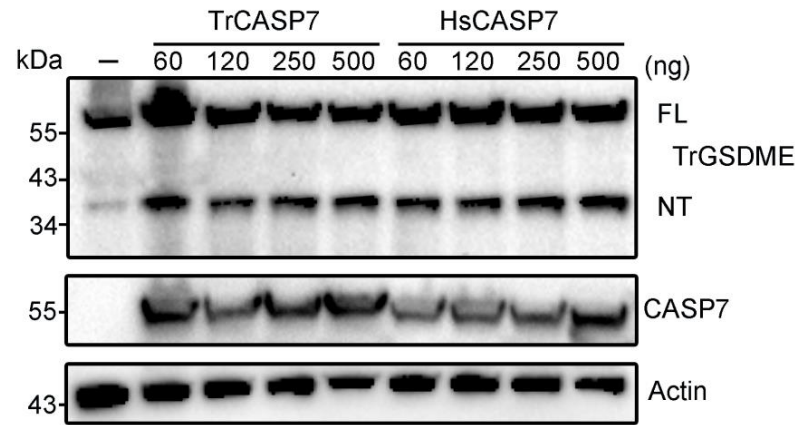

**anti-Flag**

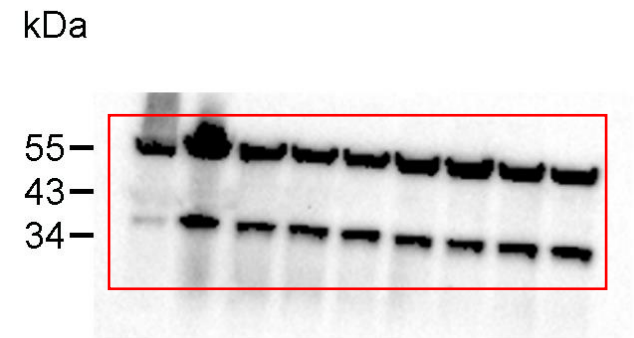

**anti-Myc**

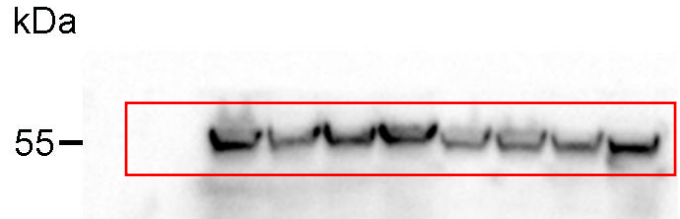

**anti-actin**

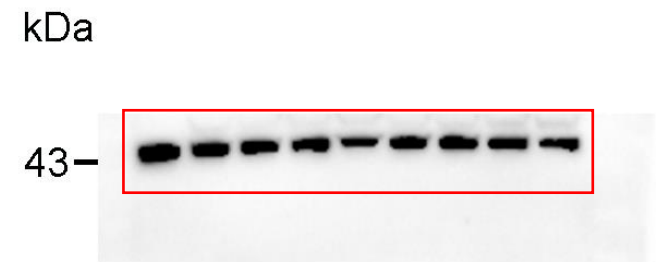

Supplement: Figure 5—figure supplement 3—source data 2. [file elife-89974-fig5-figsupp3-data2.zip › Figure 5-figure supplement 3-source data 2/Figure 5-figure supplement 3B-source data.pdf]

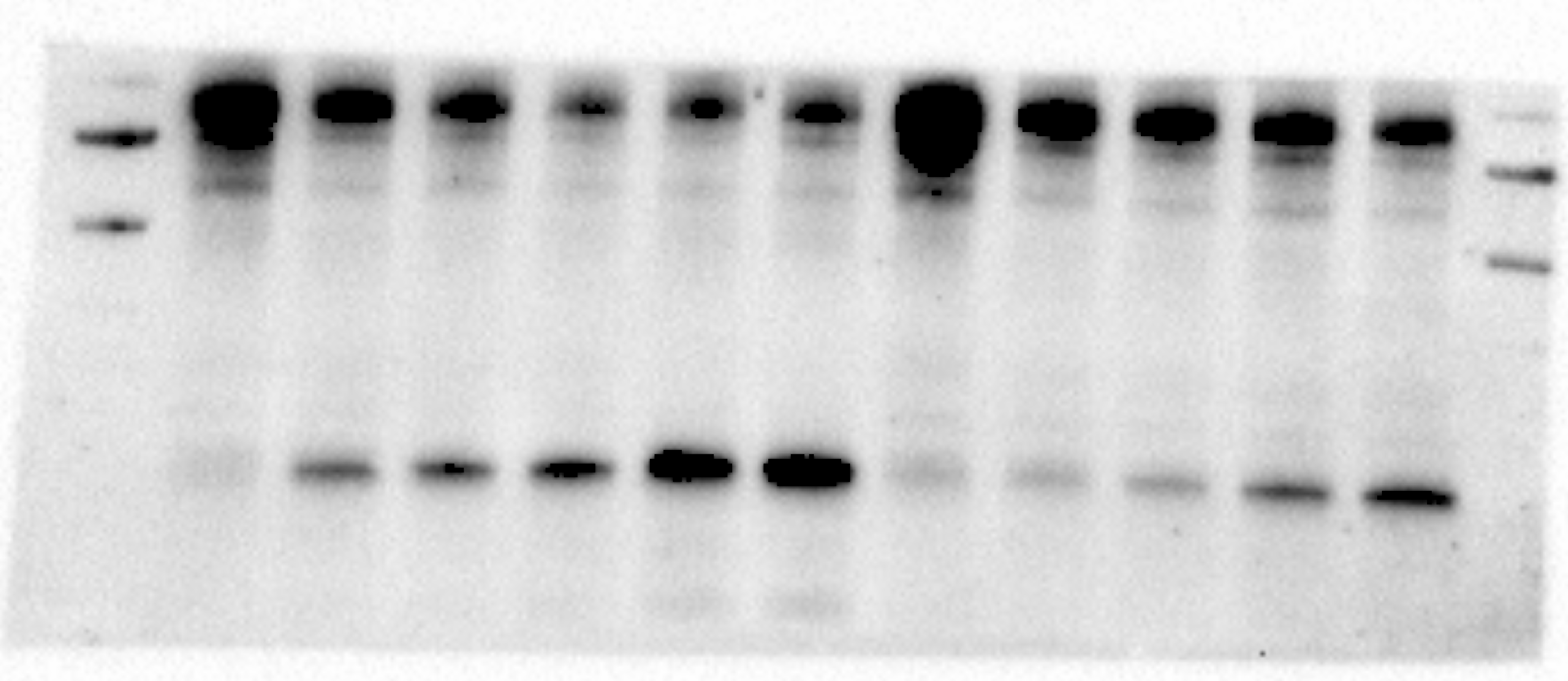

Supplement: Figure 6—source data 1. [file elife-89974-fig6-data1.zip › Figure 6-source data 1/Figure 6C-source data.tif]

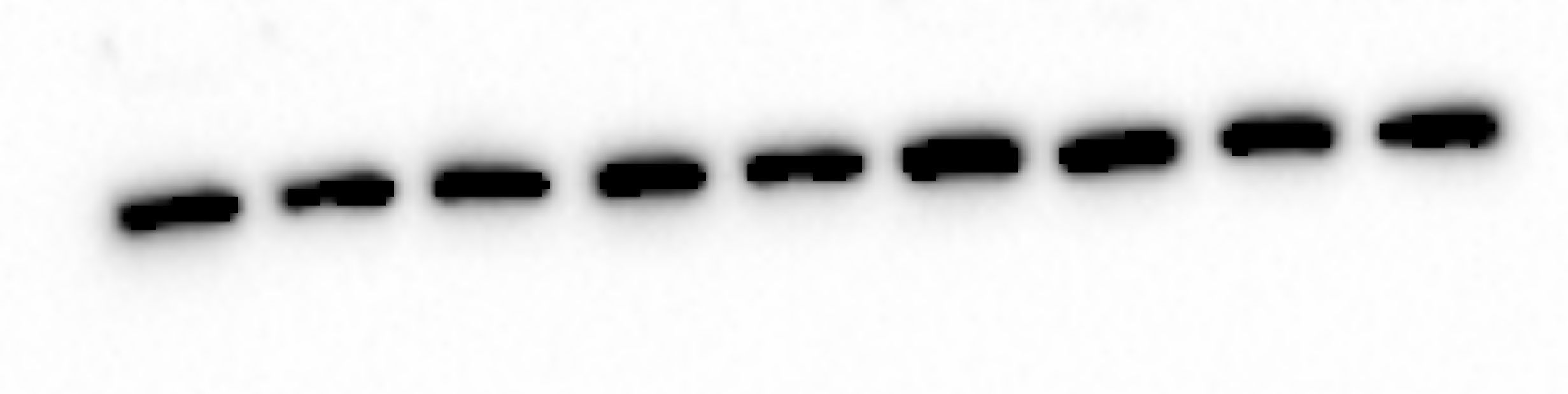

Supplement: Figure 6—source data 1. [file elife-89974-fig6-data1.zip › Figure 6-source data 1/Figure 6D-source data (anti-actin).tif]

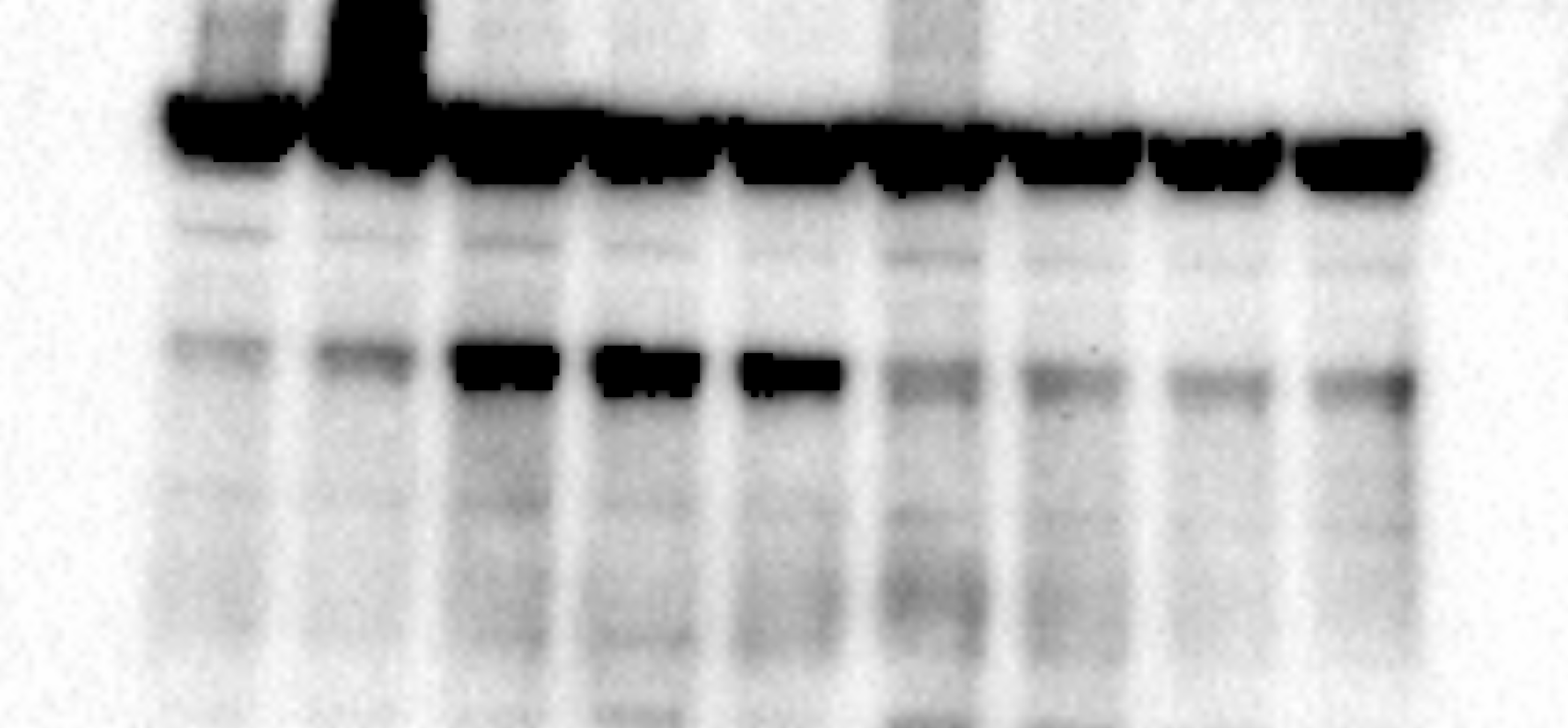

Supplement: Figure 6—source data 1. [file elife-89974-fig6-data1.zip › Figure 6-source data 1/Figure 6D-source data (anti-flag).tif]

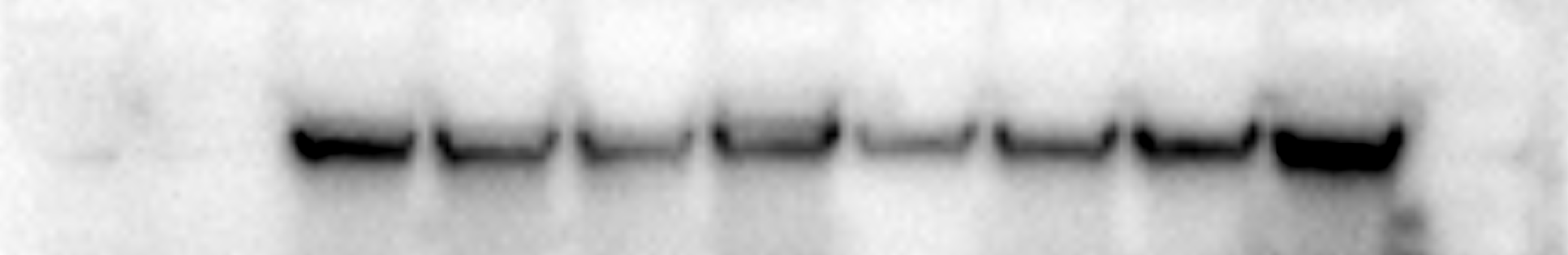

Supplement: Figure 6—source data 1. [file elife-89974-fig6-data1.zip › Figure 6-source data 1/Figure 6D-source data (anti-myc).tif]

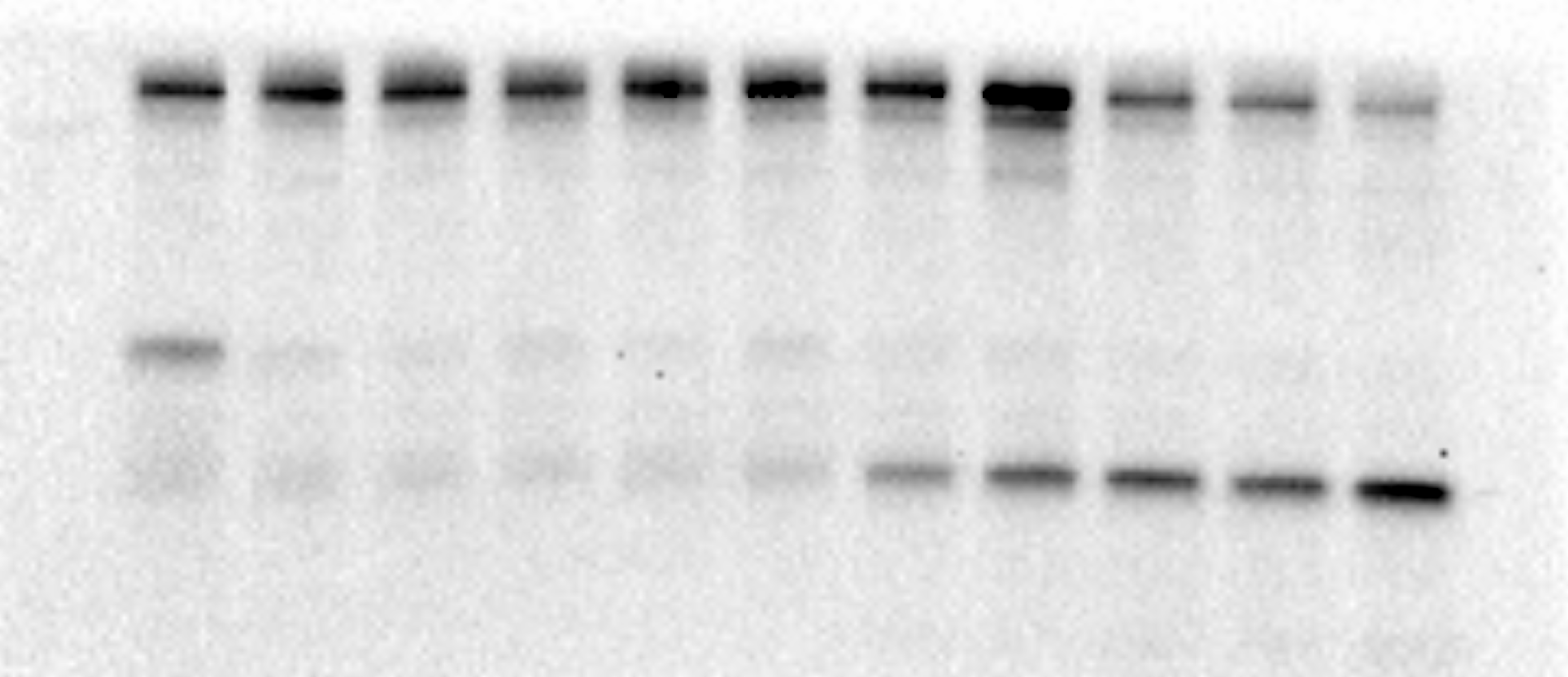

Supplement: Figure 6—source data 1. [file elife-89974-fig6-data1.zip › Figure 6-source data 1/Figure 6G-source data.tif]

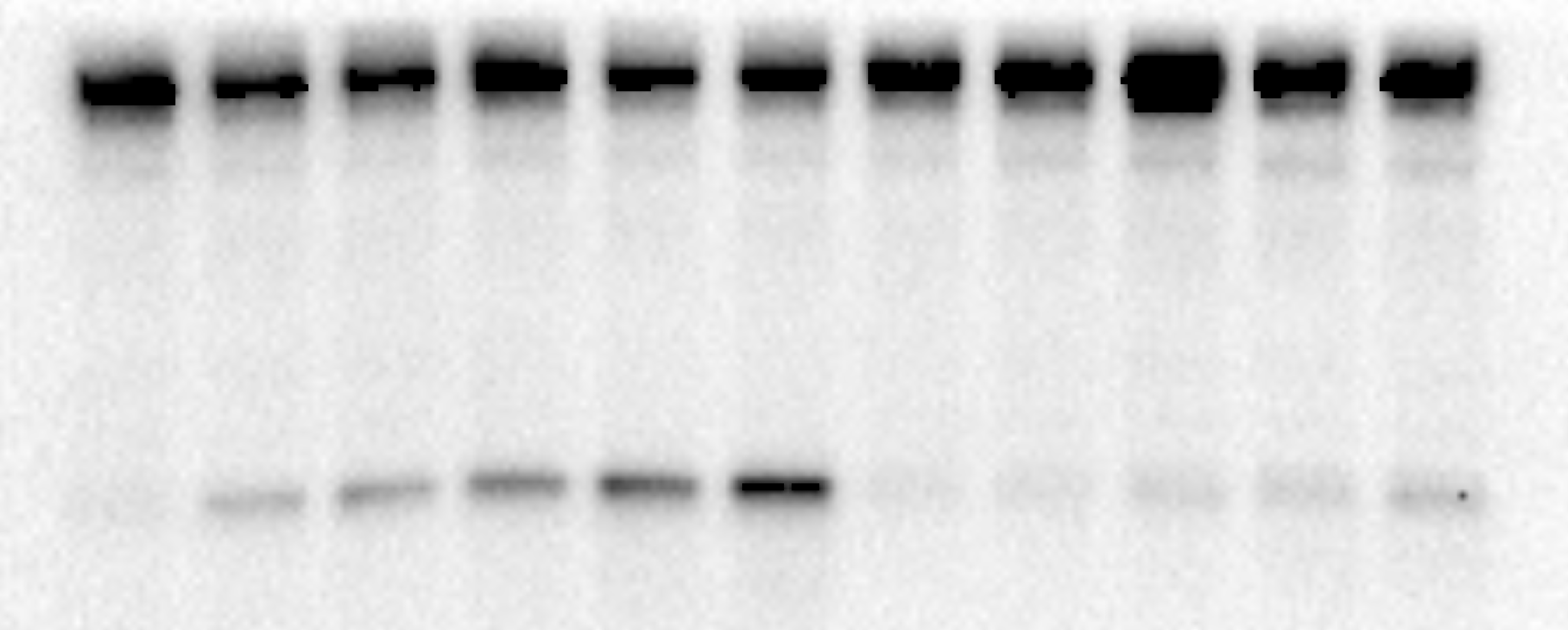

Supplement: Figure 6—source data 1. [file elife-89974-fig6-data1.zip › Figure 6-source data 1/Figure 6H-source data.tif]

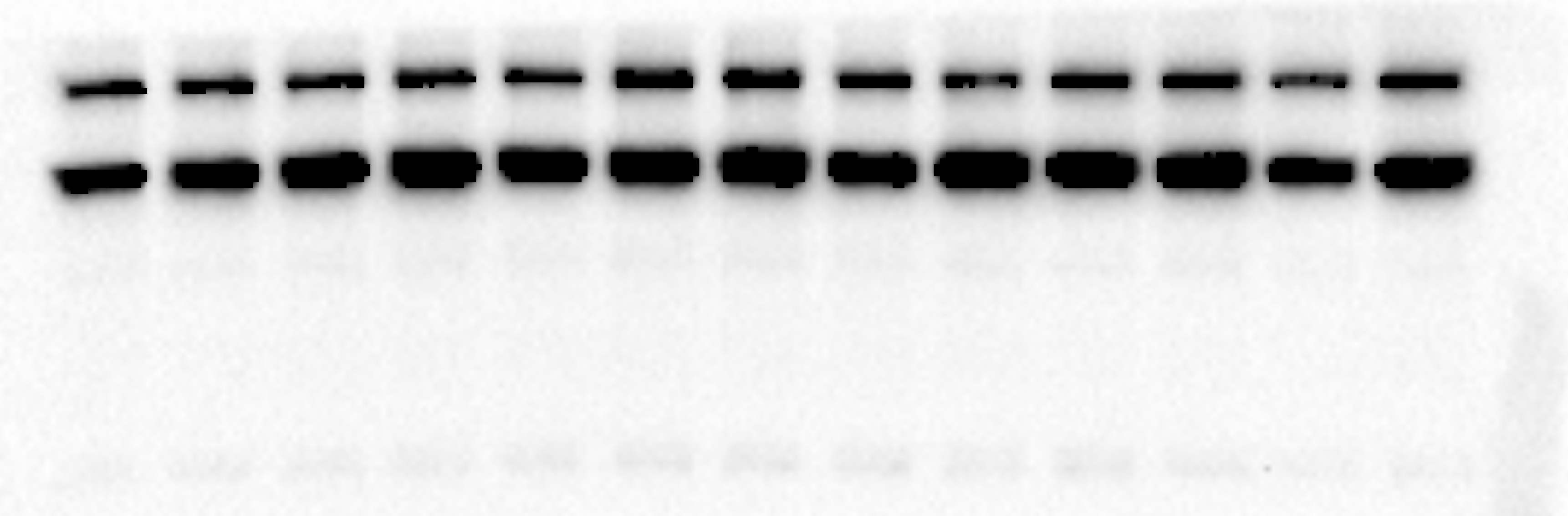

Supplement: Figure 6—source data 1. [file elife-89974-fig6-data1.zip › Figure 6-source data 1/Figure 6I-source data.tif]

**Figure 6C**

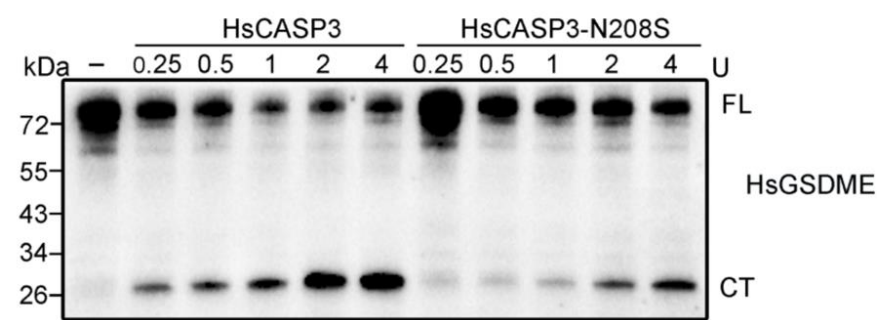

**anti-HsGSDME-CT**

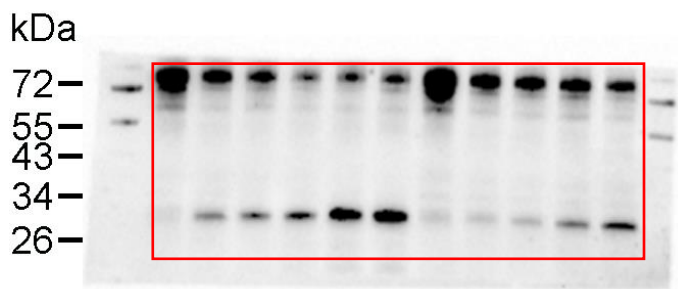

Supplement: Figure 6—source data 2. [file elife-89974-fig6-data2.zip › Figure 6-source data 2/Figure 6C-source data.pdf]

**Figure 6D**

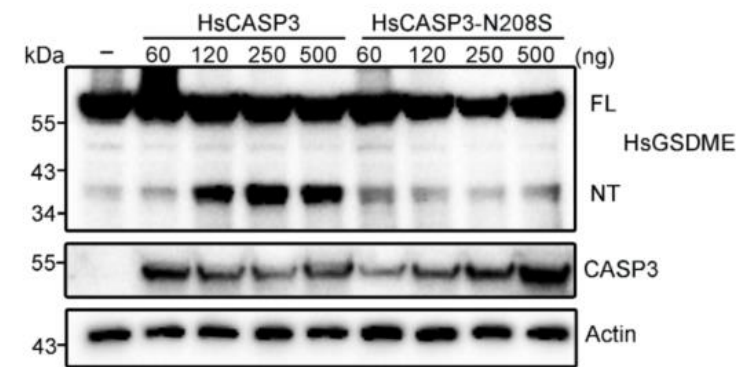

**anti-Flag**

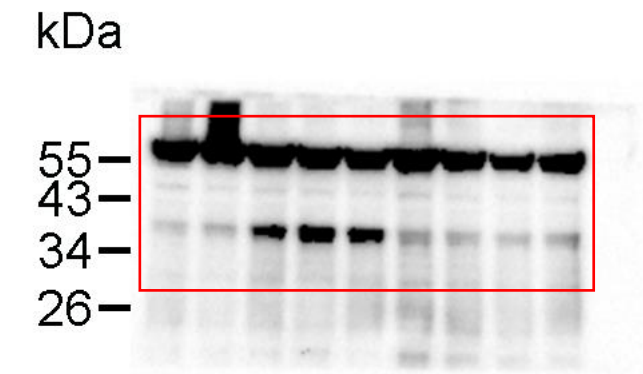

**anti-Myc**

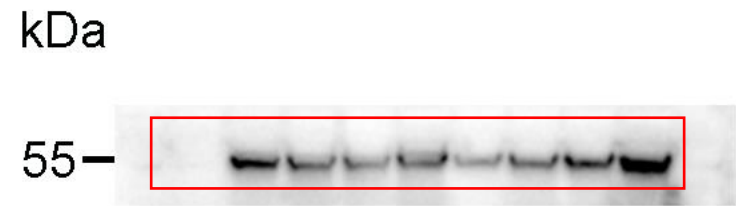

**anti-actin**

kDa

43—

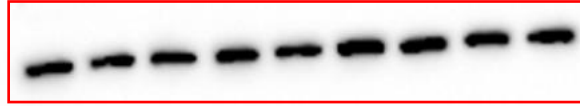

Supplement: Figure 6—source data 2. [file elife-89974-fig6-data2.zip › Figure 6-source data 2/Figure 6D-source data.pdf]

**Figure 6G**

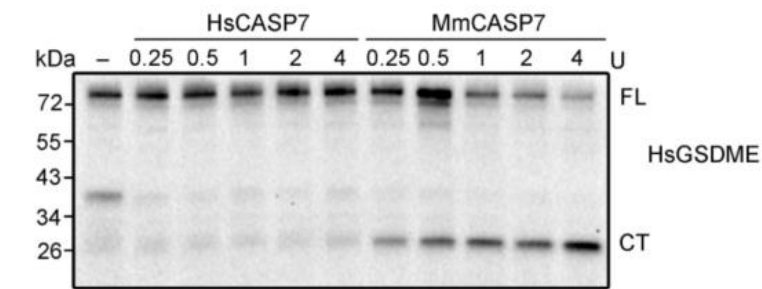

**anti-HsGSDME-CT**

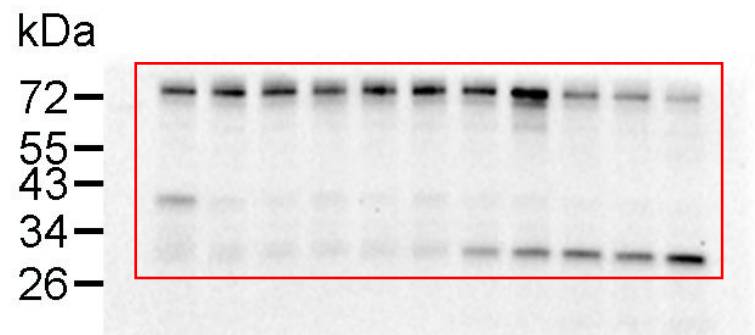

Supplement: Figure 6—source data 2. [file elife-89974-fig6-data2.zip › Figure 6-source data 2/Figure 6G-source data.pdf]

**Figure 6H**

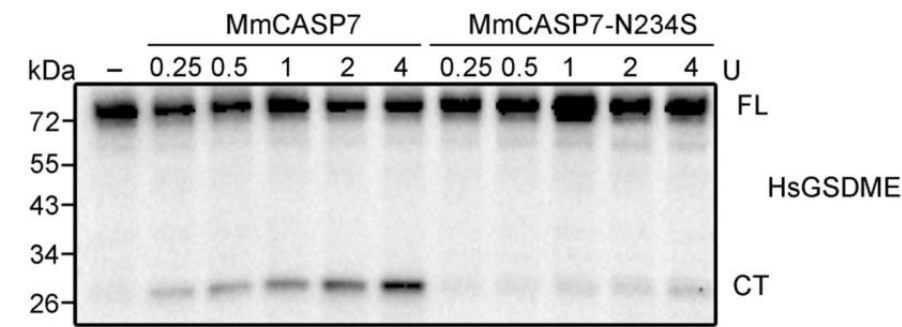

**anti-HsGSDME-CT**

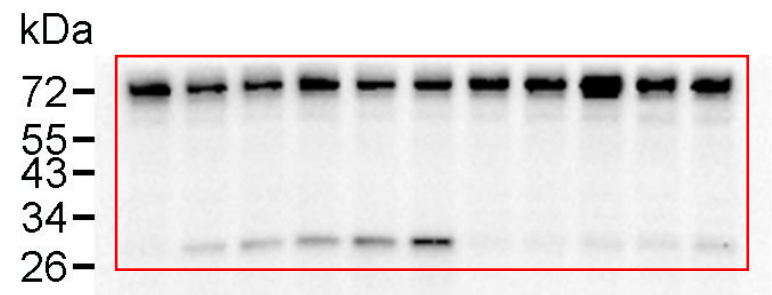

Supplement: Figure 6—source data 2. [file elife-89974-fig6-data2.zip › Figure 6-source data 2/Figure 6H-source data.pdf]

**Figure 6I**

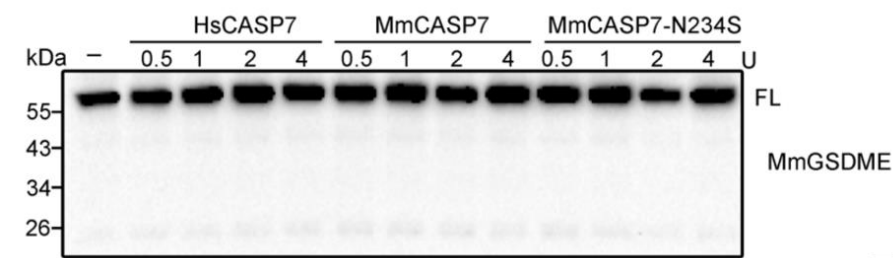

**anti-MmGSDME-CT**

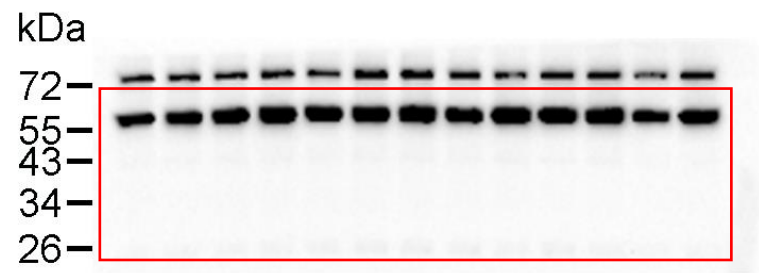

Supplement: Figure 6—source data 2. [file elife-89974-fig6-data2.zip › Figure 6-source data 2/Figure 6I-source data.pdf]

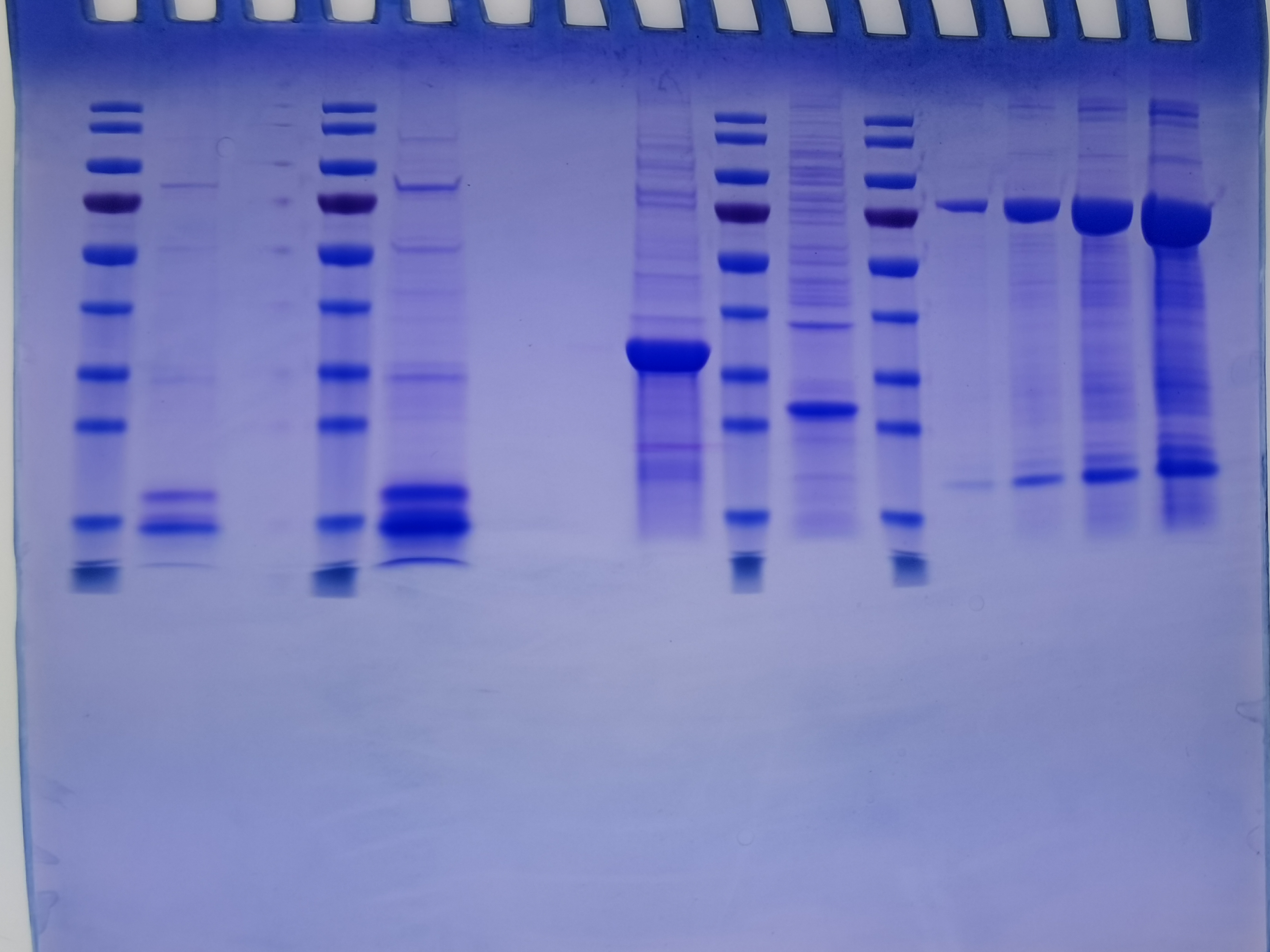

Supplement: Figure 6—figure supplement 2—source data 1. [file elife-89974-fig6-figsupp2-data1.zip › Figure 6-figure supplement 2-source data 1/Figure 6-figure supplement 2A-source data.tif]

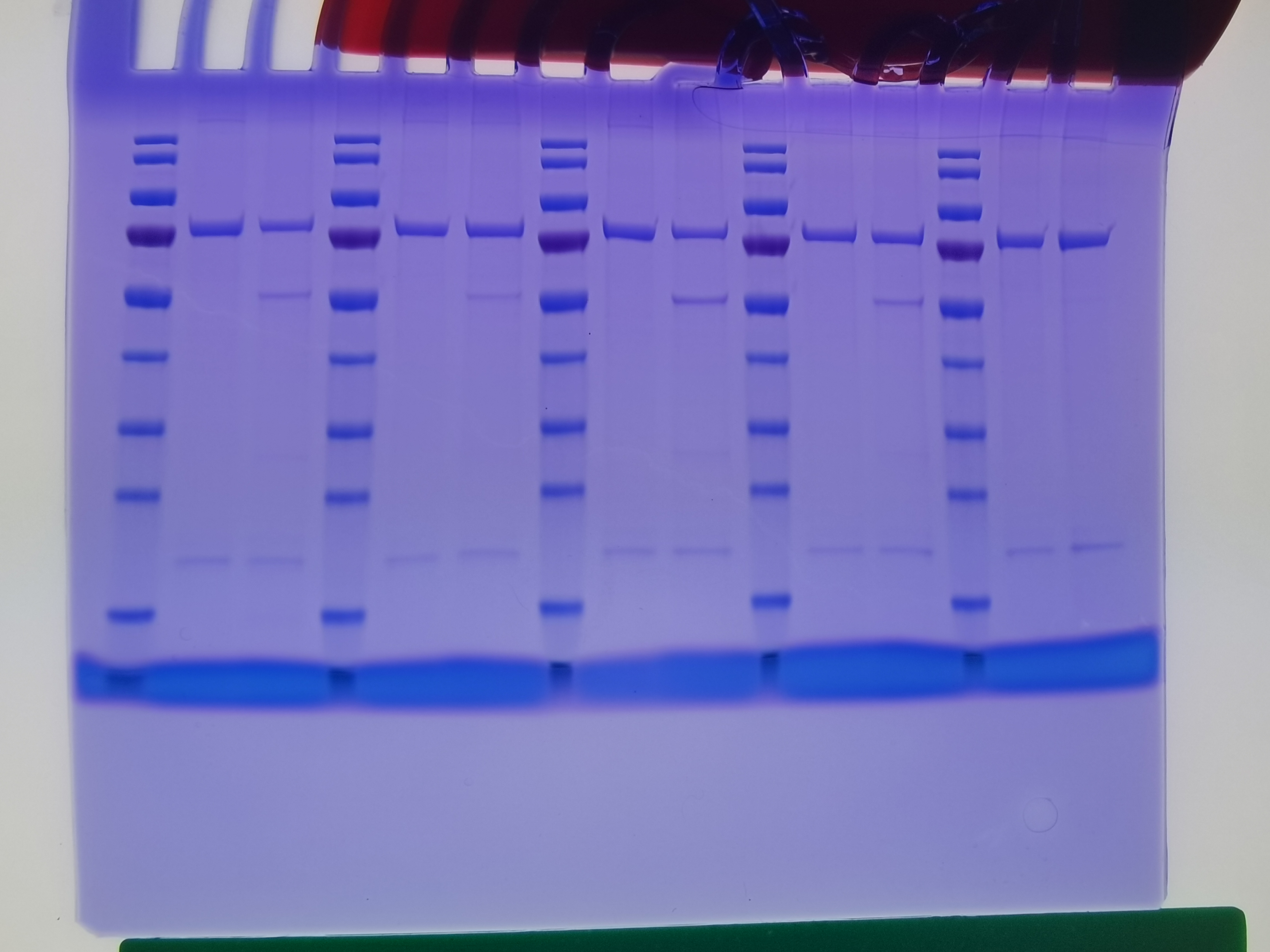

Supplement: Figure 6—figure supplement 2—source data 1. [file elife-89974-fig6-figsupp2-data1.zip › Figure 6-figure supplement 2-source data 1/Figure 6-figure supplement 2B-source data.tif]

Figure 6-figure supplement 2A

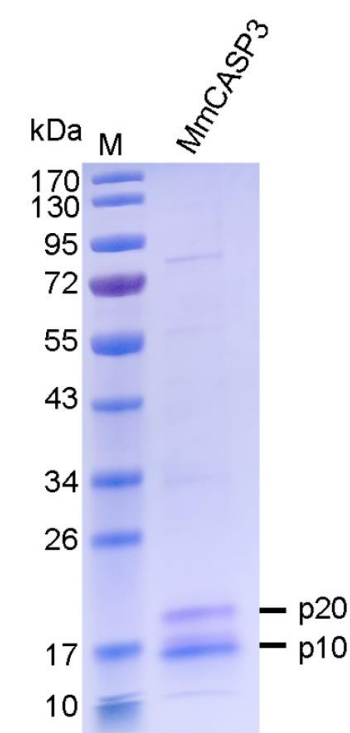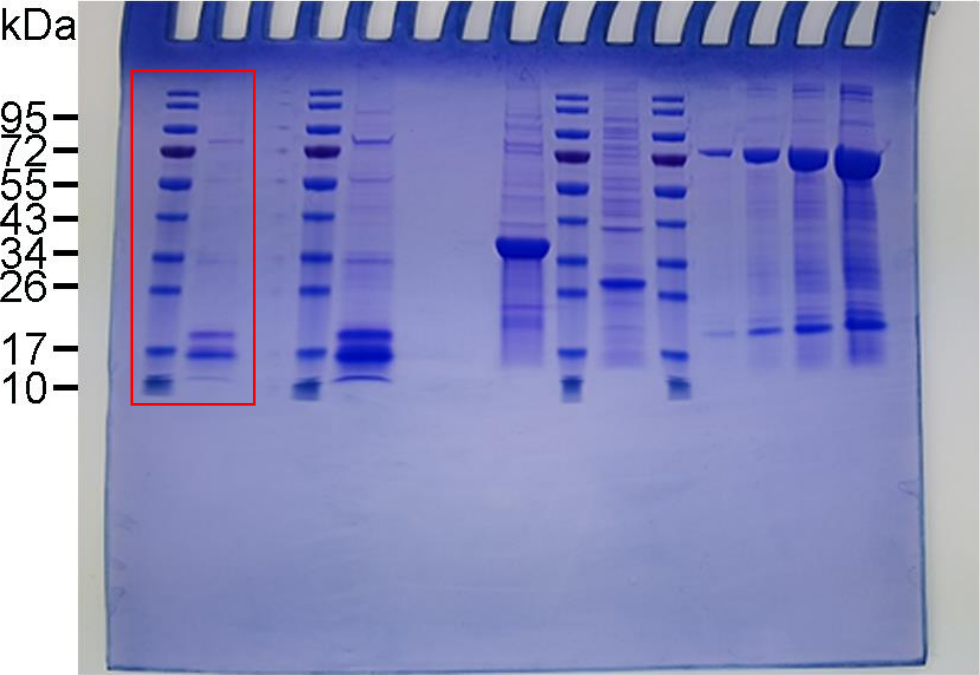

Supplement: Figure 6—figure supplement 2—source data 2. [file elife-89974-fig6-figsupp2-data2.zip › Figure 6-figure supplement 2-source data 2/Figure 6-figure supplement 2A-source data.pdf]

Figure 6-figure supplement 2B

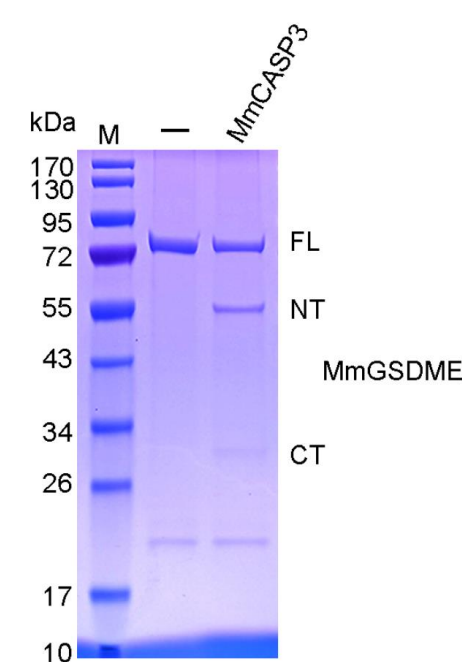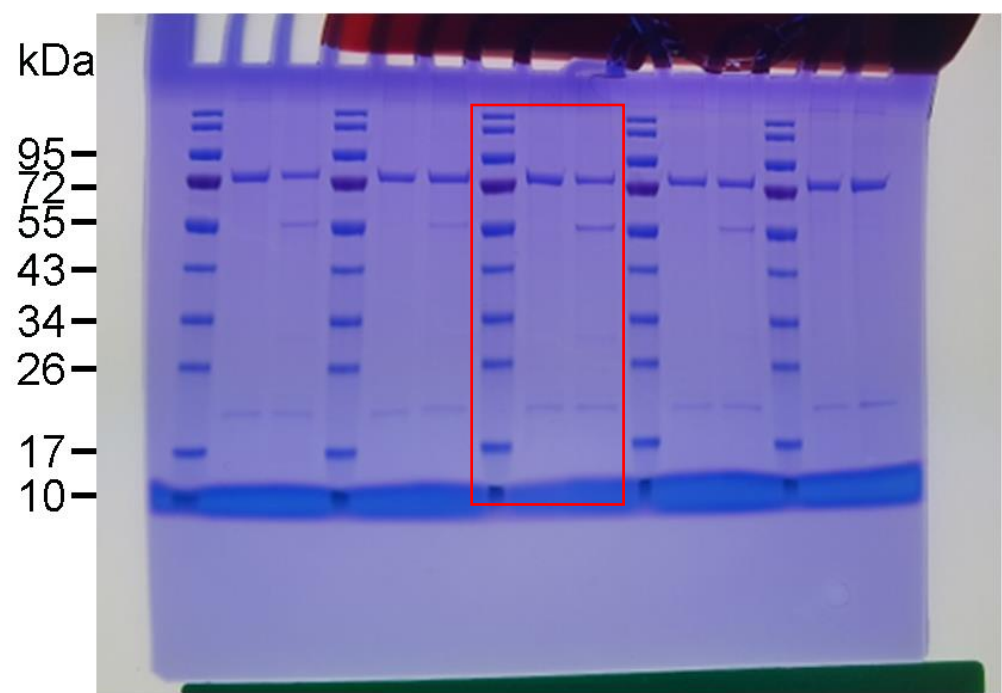

Supplement: Figure 6—figure supplement 2—source data 2. [file elife-89974-fig6-figsupp2-data2.zip › Figure 6-figure supplement 2-source data 2/Figure 6-figure supplement 2B-source data.pdf]
