# Supplementary material for: The molecular mechanism and evolutionary divergence of caspase 3/7-regulated gasdermin E activation
Source: eLife. 2024 Mar 15;12:RP89974. doi: 10.7554/eLife.89974 (PMC10942788; doi:10.7554/eLife.89974)
Supplement: Supplementary file 1. — (a) The CASP3 sequences used for WebLogo analysis in this study. (b) The CASP7 sequences used for WebLogo analysis in this study. (c) The credits for the pictures used in this study. (d) The constructs of the truncate and chimeric proteins. (e) The primers used in this study. [file elife-89974-supp1.docx]

**Supplementary File 1**

Supplementary file 1a. The CASP3 sequences used for WebLogo analysis in this study.

Supplementary file 1b. The CASP7 sequences used for WebLogo analysis in this study.

Supplementary File 1c. The credits for the pictures used in this study.

Supplementary File 1d. The constructs of the truncate and chimeric proteins.

Supplementary File 1e. Primers used in this study.

**Supplementary File 1a. The CASP3 sequences used for WebLogo analysis in this study.**

| Class | | Species | Accession Number | |
| --- | --- | --- | --- | --- |
| Mammalia  Aves  Reptilia  Amphibia  Osteichthyes | *Homo sapiens*  *Mus musculus*  *Sus scrofa*  *Canis lupus familiaris*  *Saimiri boliviensis*  *Equus caballus*  *Mesocricetus auratus*  *Pan troglodytes*  *Ovis aries*  *Capra hircus*  *Pan paniscus*  *Ailuropoda melanoleuca*  *Oryctolagus cuniculus*  *Loxodonta africana*  *Bos taurus*  *Macaca fascicularis*  *Delphinapterus leucas*  *Aotus nancymaae*  *Vulpes vulpes*  *Heterocephalus glaber*  *Bos mutus grunniens*  *Balaenoptera acutorostrata*  *Odobenus rosmarus divergens*  *Tursiops truncatus*  *Saimiri boliviensis*  *Theropithecus gelada*  *Rhinopithecus roxellana*  *Chlorocebus sabaeus*  *Arvicanthis niloticus*  *Castor canadensis*  *Urocitellus parryii*  *Neogale vison*  *Lynx rufus*  *Canis lupus dingo*  *Balaenoptera acutorostrata scammoni*  *Balaenoptera musculus*  *Vicugna pacos*  *Camelus bactrianus*  *Eptesicus fuscus*  *Pteropus alecto*  *Ochotona princeps*  *Equus quagga*  *Talpa occidentalis*  *Manis pentadactyla*  *Gracilinanus agilis*  *Corvus hawaiiensis*  *Taeniopygia guttata*  *Pyrgilauda ruficollis*  *Hirundo rustica*  *Onychostruthus taczanowskii*  *Passer montanus*  *Molothrus ater*  *Oxyura jamaicensis*  *Melopsittacus undulatus*  *Strigops habroptila*  *Aythya fuligula*  *Lonchura striata domestica*  *Geospiza fortis*  *Camarhynchus parvulus*  *Serinus canaria*  *Manacus vitellinus*  *Zonotrichia albicollis*  *Apteryx rowi*  *Numida meleagris*  *Gallus gallus*  *Tyto alba*  *Lagopus leucura*  *Pygoscelis adeliae*  *Calypte anna*  *Aquila chrysaetos chrysaetos*  *Haliaeetus leucocephalus*  *Tyto alba*  *Hirundo rustica*  *Cygnus atratus*  *Lepidothrix coronata*  *Amazona aestiva*  *Chaetura pelagica*  *Antrostomus carolinensis*  *Nipponia nippon*  *Pelecanus crispus*  *Leptosomus discolor*  *Dryobates pubescens*  *Manacus vitellinus*  *Parus major*  *Falco rusticolus*  *Numida meleagris*  *Pelodiscus sinensis*  *Gopherus evgoodei*  *Chelonia mydas*  *Dermochelys coriacea*  *Mauremys reevesii*  *Terrapene carolina triunguis*  *Trachemys scripta elegans*  *Python bivittatus*  *Pogona vitticeps*  *Zootoca vivipara*  *Podarcis muralis*  *Sceloporus undulatus*  *Lacerta agilis*  *Varanus komodoensis*  *Notechis scutatus*  *Pantherophis guttatus*  *Protobothrops mucrosquamatus*  *Eremias argus*  *Sceloporus undulatus*  *Gekko japonicus*  *Lacerta agilis*  *Notechis scutatus*  *Pseudonaja textilis*  *Sphaerodactylus townsendi*  *Thamnophis sirtalis*  *Crotalus tigris*  *Thamnophis elegans*  *Crocodylus porosus*  *Chelonoidis abingdonii*  *Chrysemys picta bellii*  *Mauremys mutica*  *Xenopus laevis*  *Bufo gargarizans*  *Rana temporaria*  *Bufo bufo*  *Geotrypetes seraphini*  *Microcaecilia unicolor*  *Nanorana parkeri*  *Cynops orientalis*  *Xenopus tropicalis*  *Rhinatrema bivittatum*  *Geotrypetes seraphini*  *Paralichthys olivaceus*  *Danio rerio*  *Dicentrarchus labrax*  *Larimichthys crocea*  *Scophthalmus maximus*  *Cynoglossus semilaevis*  *Cyprinus carpio*  *Cyprinodon variegatus*  *Cyprinodon tularosa*  *Takifugu rubripes*  *Salmo salar*  *Oncorhynchus mykiss*  *Monopterus albus*  *Nothobranchius furzeri*  *Miichthys miiuy*  *Anguilla japonica*  *Scleropages Formosus*  *Poecilia reticulata*  *Poecilia formosa*  *Oplegnathus fasciatus*  *Collichthys lucidus*  *Notothenia coriiceps*  *Poeciliopsis prolifica*  *Haplochromis burtoni*  *Cyprinus carpio*  *Cyprinus carpio*  *Electrophorus electricus*  *Amphiprion melanopus*  *Archocentrus centrarchus*  *Bagarius yarrelli*  *Oryzias melastigma*  *Oryzias latipes*  *Siniperca chuatsi*  *Kryptolebias marmoratus*  *Astyanax mexicanus*  *Miichthys miiuy*  *Xiphophorus couchianus*  *Oreochromis niloticus*  *Lutjanus peru*  *Mugil incilis* | | | P42574  P70677  Q95ND5  Q8MKI5  [NP_001266895.1](https://www.ncbi.nlm.nih.gov/protein/NP_001266895.1?report=genbank&log$=prottop&blast_rank=1&RID=D4SGPGXV016)  NP_001157433.1  Q60431  XP_009446811.1  XP_014960045.1  NP_001273018.1  XP_003823509.1  XP_002914062.1  Q8MJC3  XP_023410784.1  Q08DY9  Q2PFV2  XP_022426661.1  XP_012327995.1  XP_025874057.1  [EHB15384.1](https://www.ncbi.nlm.nih.gov/protein/EHB15384.1?report=genbank&log$=prottop&blast_rank=1&RID=D71C71FA016)  [XP_010834058.1](https://www.ncbi.nlm.nih.gov/protein/XP_010834058.1?report=genbank&log$=prottop&blast_rank=1&RID=D71HCWUW013)  [XP_007177921.2](https://www.ncbi.nlm.nih.gov/protein/XP_007177921.2?report=genbank&log$=prottop&blast_rank=1&RID=D71MV91Y013)  [XP_012422221.1](https://www.ncbi.nlm.nih.gov/protein/XP_012422221.1)  [XP_033704658.1](https://www.ncbi.nlm.nih.gov/protein/XP_033704658.1)  AAV74267.1  XP_025242051.1  XP_010376231.2  XP_007998582.1  XP_034376775.1  JAV41953.1  XP_026256383.1  XP_044081570.1  XP_046947013.1  XP_025327613.1  XP_007177921.1  XP_036694636.1  XP_006198038.1  XP_010951604.1  XP_008150114.1  XP_006916432.1  XP_004579072.1  XP_046504640.1  XP_037382298.1  XP_036750218.1  XP_044536929.1  XP_048160132.1  XP_030127125.3  XP_041335801.1  XP_039921773.1  XP_041283907.1  XP_039555833.1  XP_036238378.1  XP_035179722.1  XP_030904860.1  XP_030349011.1  XP_032043173.1  OWK64686.1  XP_005415590.1  XP_030803986.1  XP_030094646.1  XP_008923018.1  XP_005483896.1  XP_025939844.1  XP_021249041.1  NP_990056.1  XP_042663613.1  XP_042726563.1  XP_009328950.1  XP_030305365.1  XP_029861401.1  XP_010568301.1  XP_032854868.1  XP_039921766.1  XP_035413755.1  XP_017664322.1  KQK77628.1  XP_010000752.1  XP_010171751.1  XP_009473093.1  XP_009479867.1  XP_009958677.1  XP_009909179.1  XP_008923018.1  XP_015479949.1  XP_037254769.1  XP_021249038.1  XP_006128559.1  XP_030419000.1  XP_043402007.1  XP_043369528.1  XP_039396469.1  XP_029766549.1  XP_034627282.1  XP_025018785.1  XP_020658385.1  XP_034967628.1  XP_028600040.1  XP_042323800.1  XP_033017209.1  XP_044305785.1  XP_026522951.1  XP_034257179.1  XP_015687286.1  QCQ80689.1  XP_042323800.1  XP_015272894.1  XP_033017209.1  XP_026522951.1  XP_026553662.1  XP_048364960.1  XP_013910221.1  XP_039186723.1  XP_032080611.1  XP_019390228.1  XP_032656285.1  XP_005282030.1  XP_044873973.1  NP_001081225.1  XP_044145804.1  XP_040189928.1  XP_040274658.1  XP_033799250.1  XP_030047368.1  XP_018421176.1  AFN55260.1  NP_001120900.1  XP_029441076.1  XP_033799250.1  XP_019958716.1  AWP39888.1  ABC70997.1  NP_001290322.1  AVW89178.1  XP_016894801.1  XP_018965718.1  XP_015245088.1  XP_038127805.1  NP_001027871.1  NP_001133393.1  BAU69680.1  XP_020451082.1  KAF7225424.1  AHG06618.1  AYC61977.1  XP_018591577.1  XP_008417983.1  XP_007549682.1  AFM09714.1  TKS72762.1  XP_010794822.1  JAO80483.1  XP_005944370.2  AGU12796.1  AGU12795.1  XP_026884624.2  AEA08874.1  XP_030591190.1  TSN95700.1  XP_024138660.1  NP_001098140.1  ADK47519.1  XP_017278585.1  XP_022539261.1  AHG06618.1  XP_027872889.1  ADJ57601.1  QBY35776.1  QDK54780.1 |

**Supplementary File 1b. The CASP7 sequences used for the WebLogo analysis in this study.**

| Class | Species | Accession Number |
| --- | --- | --- |
| Mammalia  Aves  Reptilia  Amphibia  Osteichthyes | *Homo sapiens*  *Mus musculus*  *Mesocricetus auratus*  *Bos taurus*  *Pan troglodytes*  *Macaca fascicularis*  *Oryctolagus cuniculus*  *Ovis aries*  *Capra hircus*  *Ailuropoda melanoleuca*  *Saimiri boliviensis boliviensis*  *Equus caballus*  *Delphinapterus leucas*  *Sus scrofa*  *Pan paniscus*  *Loxodonta africana*  *Canis lupus familiaris*  *Oryctolagus cuniculus*  *Vulpes vulpes*  *Heterocephalus glaber*  *Urocitellus parryii*  *Equus asinus*  *Tachyglossus aculeatus*  *Camelus bactrianus*  *Balaenoptera acutorostrata scammoni*  *Myodes glareolus*  *Dipodomys spectabilis*  *Delphinapterus leucas*  *Eptesicus fuscus*  *Bison bison bison*  *Pteropus alecto*  *Rhinopithecus bieti*  *Balaenoptera musculus*  *Saimiri boliviensis boliviensis*  *Myotis brandtii*  *Ursus americanus*  *Neogale vison*  *Vulpes lagopus*  *Equus quagga*  *Oryctolagus cuniculus*  *Galemys pyrenaicus*  *Manis pentadactyla*  *Gracilinanus agilis*  *Corvus hawaiiensis*  *Taeniopygia guttata*  *Pyrgilauda ruficollis*  *Hirundo rustica*  *Onychostruthus taczanowskii*  *Passer montanus*  *Molothrus ater*  *Oxyura jamaicensis*  *Turdus rufiventris*  *Melopsittacus undulatus*  *Strigops habroptila*  *Aythya fuligula*  *Lonchura striata domestica*  *Geospiza fortis*  *Camarhynchus parvulus*  *Serinus canaria*  *Manacus vitellinus*  *Zonotrichia albicollis*  *Apteryx rowi*  *Numida meleagris*  *Gallus gallus*  *Tyto alba*  *Lagopus leucura*  *Pygoscelis adeliae*  *Corvus brachyrhynchos*  *Calypte anna*  *Apteryx mantelli mantelli*  *Apaloderma vittatum*  *Haliaeetus leucocephalus*  *Tyto alba*  *Lonchura striata domestica*  *Cygnus atratus*  *Cyanistes caeruleus*  *Amazona aestiva*  *Mesitornis unicolor*  *Melopsittacus undulatus*  *Athene cunicularia*  *Camarhynchus parvulus*  *Gallus gallus*  *Chaetura pelagica*  *Ficedula albicollis*  *Serinus canaria*  *Cuculus canorus*  *Corvus cornix cornix*  *Nipponia nippon*  *Pelecanus crispus*  *Balearica regulorum gibbericeps*  *Manacus vitellinus*  *Phalacrocorax carbo*  *Parus major*  *Falco rusticolus*  *Corvus hawaiiensis*  *Pelodiscus sinensis*  *Gopherus evgoodei*  *Chelonia mydas*  *Dermochelys coriacea*  *Mauremys reevesii*  *Terrapene carolina triunguis*  *Trachemys scripta elegans*  *Python bivittatus*  *Pogona vitticeps*  *Zootoca vivipara*  *Podarcis muralis*  *Sceloporus undulatus*  *Anolis carolinensis*  *Varanus komodoensis*  *Lacerta agilis*  *Notechis scutatus*  *Notechis scutatus*  *Pantherophis guttatus*  *Python bivittatus*  *Protobothrops mucrosquamatus*  *Zootoca vivipara*  *Podarcis muralis*  *Sceloporus undulatus*  *Anolis carolinensis*  *Varanus komodoensis*  *Varanus komodoensis*  *Lacerta agilis*  *Notechis scutatus*  *Pantherophis guttatus*  *Protobothrops mucrosquamatus*  *Pseudonaja textilis*  *Sphaerodactylus townsendi*  *Crotalus tigris*  *Thamnophis elegans*  *Chelonoidis abingdonii*  *Mauremys mutica*  *Chrysemys picta bellii*  *Trachemys scripta elegans*  *Crocodylus porosus*  *Xenopus laevis*  *Lithobates catesbeianus*  *Bufo gargarizans*  *Rana temporaria*  *Bufo bufo*  *Geotrypetes seraphini*  *Microcaecilia unicolor*  *Nanorana parkeri*  *Cynops orientalis*  *Xenopus tropicalis*  *Xenopus tropicalis*  *Rhinatrema bivittatum*  *Paralichthys olivaceus*  *Danio rerio*  *Dicentrarchus labrax*  *Larimichthys crocea*  *Scophthalmus maximus*  *Cynoglossus semilaevis*  *Oncorhynchus kisutch*  *Cyprinus carpio*  *Cyprinodon variegatus*  *Fundulus heteroclitus*  *Cyprinodon tularosa*  *Takifugu rubripes*  *Salmo salar*  *Oncorhynchus mykiss*  *Ictalurus punctatus*  *Monopterus albus*  *Nothobranchius furzeri*  *Alosa alosa*  *Alosa sapidissima*  *Anabarilius grahami*  *Salvelinus alpinus*  *Salvelinus alpinus*  *Gadus morhua*  *Labrus bergylta*  *Toxotes jaculatrix*  *Collichthys lucidus*  *Notothenia coriiceps*  *Melanotaenia boesemani*  *Clarias magur*  *Anabas testudineus*  *Chelmon rostratus*  *Anguilla anguilla*  *Trematomus bernacchii*  *Pimephales promelas*  *Bagarius yarrelli*  *Seriola dumerili*  *Xiphophorus hellerii*  *Sebastes umbrosus*  *Parambassis ranga*  *Oryzias melastigma*  *Megalops cyprinoides*  *Salarias fasciatus*  *Salvelinus namaycush* | P55210  P97864  P55214  XP_002698555.1  XP_003825650.1  XP_011822901.1  XP_002718761.1  XP_042094975.1  XP_005698554.1  [XP_034519188.1](https://www.ncbi.nlm.nih.gov/protein/XP_034519188.1?report=genbank&log$=prottop&blast_rank=1&RID=D23B7GXH016)  [XP_034519188.1](https://www.ncbi.nlm.nih.gov/protein/XP_034519188.1?report=genbank&log$=prottop&blast_rank=1&RID=D23CUADR016)  [XP_014588811.1](https://www.ncbi.nlm.nih.gov/protein/XP_014588811.1?report=genbank&log$=prottop&blast_rank=1&RID=D23ENPZ1016)  [XP_022424428.1](https://www.ncbi.nlm.nih.gov/protein/XP_022424428.1?report=genbank&log$=prottop&blast_rank=1&RID=D23H4PTM013)  [XP_020928977.1](https://www.ncbi.nlm.nih.gov/protein/XP_020928977.1?report=genbank&log$=prottop&blast_rank=1&RID=D23V08DC016)  [XP_003825650.1](https://www.ncbi.nlm.nih.gov/protein/XP_003825650.1?report=genbank&log$=prottop&blast_rank=1&RID=D23X9W00013)  [XP_010587277.1](https://www.ncbi.nlm.nih.gov/protein/XP_010587277.1?report=genbank&log$=prottop&blast_rank=3&RID=D242WCE1016)  [XP_005637795.1](https://www.ncbi.nlm.nih.gov/protein/XP_005637795.1?report=genbank&log$=prottop&blast_rank=2&RID=D247044R013)  [XP_002718761.1](https://www.ncbi.nlm.nih.gov/protein/XP_002718761.1?report=genbank&log$=prottop&blast_rank=1&RID=D249T0AU016)  [XP_025849552.1](https://www.ncbi.nlm.nih.gov/protein/XP_025849552.1?report=genbank&log$=prottop&blast_rank=1&RID=D24EE87H016)  [XP_004838949.1](https://www.ncbi.nlm.nih.gov/protein/XP_004838949.1?report=genbank&log$=prottop&blast_rank=2&RID=D24HF1BG013)  XP_026244955.1  XP_014723324.1  XP_038614071.1  XP_010968130.1  XP_007173176.1  XP_048279394.1  XP_042545561.1  XP_022424428.1  XP_028005288.1  XP_010857610.1  XP_006920912.1  XP_017747992.1  XP_036685506.1  XP_010331177.1  XP_005874576.1  XP_045659135.1  XP_044096500.1  XP_041601933.1  XP_046510739.1  XP_002718761.1  [KAG8517409.1](https://www.ncbi.nlm.nih.gov/protein/KAG8517409.1?report=genbank&log$=prottop&blast_rank=1&RID=D24RFGZJ013)  XP_036754817.1  XP_044521530.1  XP_048166914.1  XP_030132303.3  XP_041317496.1  XP_039927077.1  XP_041271849.1  XP_039565350.1  XP_036241577.1  XP_035186324.1  KAF4791228.1  XP_005154576.1  XP_030344254.1  XP_032047208.1  XP_021381698.1  XP_005416425.1  XP_030807062.1  XP_009085424.1  XP_029814357.1  XP_005481147.1  XP_025925619.1  XP_021255526.1  XP_040530979.1  XP_032844696.2  XP_042719653.1  XP_009324216.1  XP_008632648.1  XP_030309482.1  XP_013797196.1  XP_009876249.1  XP_010563034.1  XP_032844693.2  OWK56987.1  XP_035403725.1  XP_023785639.1  KQK80332.1  XP_010180183.1  XP_005154576.1  XP_026707509.1  XP_030807062.1  XP_421764.3  XP_010006222.1  XP_005048826.1  XP_009085424.1  XP_009568871.1  XP_039410202.1  XP_009474328.1  XP_009483464.1  XP_010295946.1  XP_017924402.1  XP_009508983.1  XP_015489222.1  XP_037256573.1  XP_048166914.1  XP_006135034.1  XP_030424754.1  XP_037760318.1  XP_038265833.1  XP_039402417.1  XP_026506928.1  XP_034632909.1  XP_007441954.1  XP_020635566.1  XP_034988803.1  XP_028586060.1  XP_042313006.1  XP_008112942.1  KAF7253514.1  XP_033005524.1  XP_026526250.1  XP_026526259.1  XP_034296056.1  XP_007441954.1  XP_015676047.1  XP_034988803.1  XP_028586060.1  XP_042313006.1  XP_003223489.1  KAF7253514.1  XP_044295172.1  XP_033005524.1  XP_026526250.1  XP_034296056.1  XP_015676047.1  XP_026560589.1  XP_048360798.1  XP_039208641.1  XP_032081709.1  XP_032631043.1  XP_044881213.1  XP_042711209.1  XP_034632910.1  XP_019411759.1  BAA94748.1  ACO51875.1  XP_044152860.1  XP_040217514.1  XP_040291577.1  XP_033799427.1  XP_030058570.1  XP_018419981.1  AFN55259.1  NP_001016299.1  CAJ82745.1  XP_029466303.1  XP_019965790.1  AWP39893.1  CBN81450.1  XP_010740374.1  XP_047183439.1  XP_024912944.1  XP_020329675.1  XP_042591656.1  XP_015245976.1  XP_012710613.2  XP_029690759.1  XP_029690759.1  XP_014012537.2  QWC93456.1  XP_017338665.1  XP_020460887.1  KAF7204369.1  XP_048088911.1  XP_041939757.1  ROI81868.1  XP_023842909.1  XP_023861943.1  XP_030195895.1  XP_020506858.1  XP_040923277.1  TKS89629.1  XP_010765636.1  XP_041830322.1  KAF5896512.1  XP_026207209.1  XP_041817955.1  XP_035260198.1 XP_033975792.1  KAG1954935.1  TSK14754.1  XP_022598445.1  XP_032429481.1  XP_037611938.1  XP_028285718.1  KAF6724972.1  XP_036387401.1  XP_029954337.1  XP_038866282.1 |

**Supplementary File 1c. The credits for the pictures used in this study.**

| Icon | Figure Origin |
| --- | --- |
| Monotremata  Didelphimorphia  Diprotodontia  Afrosoricida  Macroscelidea  Pilosa  Primates  Rodentia  Lagomorpha  Eulipotyphla  Perissodactyla  Artiodactyla  Carnivora  Pholidota  Chiroptera  *Homo sapiens*  Avian  Reptilia  Amphibia  Actinopterygii | Becky-Barnes  Daniel-Stadtmauer  Gavin-Prideaux  Mo-Hassan  uncredited  Xavier-A-Jenkins  T-Michael-Keesey  Jiro-Wada  Margot-Michaud  Becky-Barnes  Mercedes-Yrayzoz-vectorized-by-T-Michael-Keesey  DFoidl-modified-by-T-Michael-Keesey  Chlo-Schmidt  Steven-Traver  Margot-Michaud  NASA  Ferran Sayol  Gabriela Palomo-Munoz  Yusan Yang  Milton Tan |

**Supplementary File 1d.** **The constructs of the truncate and chimeric proteins.**

| Truncate/chimeric protein | Amino acid sequence |
| --- | --- |
| TrGSDME-NT  TrGSDME-CT  HsNT-TrCT  TrNT-HsCT  Hsp20-Trp10  Trp20-Hsp10 | 1-258 aa of TrGSDME  259-474 aa of TrGSDME  1-270 aa of HsGSDME + 259-274 aa of TrGSMDE  1-258 aa of TrGSDME + 271-496 aa of HsGSDME  1-198 aa of HsCASP7 + 207-313 aa of TrCASP7  1-206 aa of TrCASP7 + 199-303 aa of HsCASP7 |

**Supplementary File 1e.** **Primers used in this study.**

| Primer | Sequence (5’-3’) |
| --- | --- |
| Clone primers  TrCASP3 forward  TrCASP3 reverse  TrCASP7 forward  TrCASP7 reverse  TrGSDME forward  TrGSDME reverse  Recombiantion primers  TrCASP3 forward  TrCASP3 reverse  TrCASP7 forward  TrCASP7 reverse  TrGSDME forward  TrGSDME reverse  TrGSDME-D255R forward  TrGSDME-D255R reverse  TrGSDME-D258A forward  TrGSDME-D258A reverse  HsNT-TrCT forward  HsNT-TrCT reverse  TrNT-HsCT forward  TrNT-HsCT reverse  Hsp20-Trp10 forward  Hsp20-Trp10 reverse  Trp20-Hsp10 forward  Trp20-Hsp10 reverse  MmGSDME-N234S forward  MmGSDME-N234S reverse  HsGSDME-Δ261-266 forward  HsGSDME-Δ261-266 reverse  HsGSDME-Δ281-296 forward  HsGSDME-Δ281-296 reverse  Overexpression preimers  TrGSDME-FL forward  TrGSDME-FL reverse  TrGSDME-NT forward  TrGSDME-NT reverse  TrGSDME-CT forward  TrGSDME-CT reverse  TrCASP7 forward  TrCASP7 reverse  TrCASP3 forward  TrCASP3 reverse  HsGSDME forward  HsGSDME reverse  HsCASP7 forward  HsCASP7 reverse  HsCASP3 forward  HsCASP3 reverse  MmCASP7 forward  MmCASP7 reverse | ATGTCGGCCAACGGACC  GGAAAAATACATCTCTTTGGTCAGC  ATGCAGATGGCTGGAGAACC  GTTAAAGTACAGTTCTTTTGTCAGCATCG  ATGTTTTCCAAGGCCACGG  ATCGATAAAATCCGTTTCAGACTTTG  TAAGAAGGAGATATACATATGATGT  CGGCCAACGGACC  GTGGTGGTGGTGGTGCTCGAGGGA  AAAATACATCTCTTTGGTCAGC  TAAGAAGGAGATATACATATGATGCA  GATGGCTGGAGAACC  GTGGTGGTGGTGGTGCTCGAGGTTAA  AGTACAGTTCTTTTGTCAGCATCG  TAAGAAGGAGATATACATATGATGTTT  TCCAAGGCCACGG  GTGGTGGTGGTGGTGCTCGAGATCGA  TAAAATCCGTTTCAGACTTTG  TGGGAATCCCCGAGAGCGGTGGATGG  TCGTTGTCT  AGACAACGACCATCCACCGCTCTCGG  GGATTCCCA  AGACAACGACCATCCACCGCTCTCGG  GGATTCCCA  AACGACCATCCACCGCTCTCGGGGATT  CCCA  TAAGAAGGAGATATACATATGATGTT  TGCCAAAGCAACCAGG  GTGGTGGTGGTGGTGCTCGAGATCG  ATAAAATCCGTTTCAGACTTTG  TAAGAAGGAGATATACATATGATGTT  TTCCAAGGCCACGG  GTGGTGGTGGTGGTGCTCGAGTGAA  TGTTCTCTGCCTAAAGC  TAAGAAGGAGATATACATATGATGG  CAGATGATCAGGGCTG  GTGGTGGTGGTGGTGCTCGAGGTTA  AAGTACAGTTCTTTTGTCAGCATC  TAAGAAGGAGATATACATATGATGG  CTGGAGAACCCACTGAG  GTGGTGGTGGTGGTGCTCGAGTTGA  CTGAAGTAGAGTTCCTTGGTGA  GTTATTACTCATGGAGGAGCCCAGG  GAAAG  CTTTCCCTGGGCTCCTCCATGAGTA  ATAAC  CCAGGATGGACATCCATTTGCGGAG  CTGC  CAAATGGATGTCCATCCTGGGAAGAT  ATCCCAT  CCTGGTCTTT GACATGCCAGATGCTG  CGCA  CTGGCATGTCAAAGACCAGGGGGTCC  AGGTAGA  TCAGATCTCGAGCTCAAGCTTATGTTT  TCCAAGGCCACGG  CATGGTGGCGACCGGTGGATCATCGA  TAAAATCCGTTTCAGACTTTG  TCAGATCTCGAGCTCAAGCTTATGTTT  TCCAAGGCCACGG  CATGGTGGCGACCGGTGGATCGTCTAC  AGCATCAGGAGACTCC  TCAGATCTCGAGCTCAAGCTTATGGGCC  GGTGCCTGGA  CATGGTGGCGACCGGTGGATCATCGA  TAAAATCCGTTTCAGACTTTG  TTCTGAAGAGGACTTGAATTCAATGG  CTGGAGAACCCACTGAG  ACGACTCACTATAGTTCTAGATCAGT  TAAAGTACAGTTCTTTTGTCAGC  TTCTGAAGAGGACTTGAATTCAAATG  TCGGCCAACGGACC  ACGACTCACTATAGTTCTAGAGGAA  AAATACATCTCTTTGGTCAGC  CAAGCTTGCGGCCGCGAATTCAATGT  TTGCCAAAGCAACCAGG  ACGACTCACTATAGTTCTAGATCATG  AATGTTCTCTGCCTAAAGC  TTCTGAAGAGGACTTGAATTCAATGG  CAGATGATCAGGGCTG  ACGACTCACTATAGTTCTAGACTATTG  ACTGAAGTAGAGTTCCTTGGTG  TTCTGAAGAGGACTTGAATTCAATGG  AGAACACTGAAAACTCAGTGG  ACGACTCACTATAGTTCTAGATTAGTG  ATAAAAATAGAGTTCTTTTGTGAGC  TTCTGAAGAGGACTTGAATTCAATGA  CCGATGATCAGGACTGTGC  ACGACTCACTATAGTTCTAGATCAAC  GGCTGAAGTACAGCTCTTTGG |
